# Deletion of a distal *IRF4* element prevents inflammation-induced reprogramming of human regulatory T cell fate

**DOI:** 10.1038/s41590-026-02655-8

**Published:** 2026-09-21

**Authors:** Patrick Ho, Alexander Vu, Joey Leung, Wendy Rosenthal, Vasilis Ntranos, Jeffrey A. Bluestone, Qizhi Tang

**Affiliations:** 1 Diabetes Center, University of California, San Francisco, San Francisco, CA USA; 2 Department of Surgery, University of California, San Francisco, San Francisco, CA USA; 3 Gladstone-UCSF Institute of Genomic Immunology, San Francisco, CA USA; 4 Present Address: Medizinische Klinik und Poliklinik II, Universitätsklinikum Würzburg, Würzburg, Germany

**Keywords:** Immunosuppression, Autoimmunity, Peripheral tolerance

## Abstract

Regulatory T (T_reg_) cells in mice can lose lineage identity and acquire proinflammatory functions, but whether human T_reg_ cells are similarly susceptible to cytokine-driven destabilization remains unclear. Here we established an in vitro model of human T_reg_ cell destabilization defined by silencing of the lineage-specifying transcription factor FOXP3, loss of suppressive function and acquisition of proinflammatory activity. Single-cell chromatin accessibility and transcriptomic profiling revealed a genome-wide increase in accessibility at AP-1-binding sites, including a putative regulatory element distal to *IRF4*. Increased accessibility at this element correlated with increased IRF4 expression during T_reg_ cell destabilization, and its excision conferred resistance to inflammatory cytokine-induced reprogramming. Conversely, forced expression of IRF4 together with BATF promoted T_reg_ cell destabilization. These data identify a distal IRF4 regulatory element as a critical node enabling heightened AP-1-IRF4 cooperative activity to drive T_reg_ cell destabilization, with implications for the design of more stable and effective T_reg_ cell-based therapies.

## Abstract (web summary)

Tang and colleagues identify a distal IRF4 chromatin regulatory region that governs human T_reg_ cell stability.

## Main

Regulatory T (T_reg_) cells are a specialized subset of CD4^+^ T cells characterized by constitutive FOXP3 expression and an ability to suppress the activities of other immune cells. T_reg_ cells play a dominant role in peripheral immune tolerance through a myriad of mechanisms that dampen antigen presentation, compete for T cell growth factors, convert metabolites, promote tissue repair and induce neighboring cells to develop tolerogenic activities^1^. The consequences of T_reg_ cell dysfunction are exemplified by *FOXP3* mutations that prevent T_reg_ cell differentiation, leading to systemic immune dysregulation in mice and humans^2^. Boosting or infusing T_reg_ cells has shown remarkable efficacy in many preclinical models of transplantation and autoimmune diseases, which has inspired clinical translation of T_reg_ cell-enhancing strategies to promote immune tolerance^3^. However, lineage-tracing studies have shown that mouse T_reg_ cells can undergo epigenetic reprogramming within chronically inflamed environments^4,5^. The resulting ex-Foxp3 cells show reduced suppressive activity and acquisition of proinflammatory and cytotoxic functions. Some evidence suggests that human T_reg_ cells can also lose lineage identity in chronic inflammation^6–9^, but the molecular mechanisms underlying T_reg_ cell destabilization are not well understood.

T_reg_ cell identity depends on a unique epigenetic landscape marked by hundreds of differentially methylated regions that guide T_reg_ cell-specific transcriptional programs^10^. For example, the T_reg_ cell-exclusive DNA demethylation at the *FOXP3* conserved noncoding sequence (CNS)2 allows an embedded enhancer to drive constitutive FOXP3 expression. This aptly termed T_reg_-specific demethylated region (TSDR) is widely considered the most reliable indicator of T_reg_ cell lineage commitment^11^. Although the stepwise imprinting of T_reg_ cell epigenetic signatures during thymic T_reg_ cell development has been mapped, maintenance of the T_reg_ cell epigenome in the periphery remains poorly understood. In particular, several transcription factors (TFs) have been reported to reinforce or undermine T_reg_ cell identity^12^, and the likely interplay between dysregulated gene regulatory networks and epigenetic rewiring in T_reg_ cell fate decisions requires more thorough investigation. Here we present an in vitro model of inflammatory cytokine-driven human T_reg_ cell instability, which enabled a molecular dissection of human T_reg_ cell lineage maintenance and the development of cell engineering strategies to preserve T_reg_ cell identity and function.

## Results

### In vitro model of human T_reg_ cell instability

**Fig. 1:**
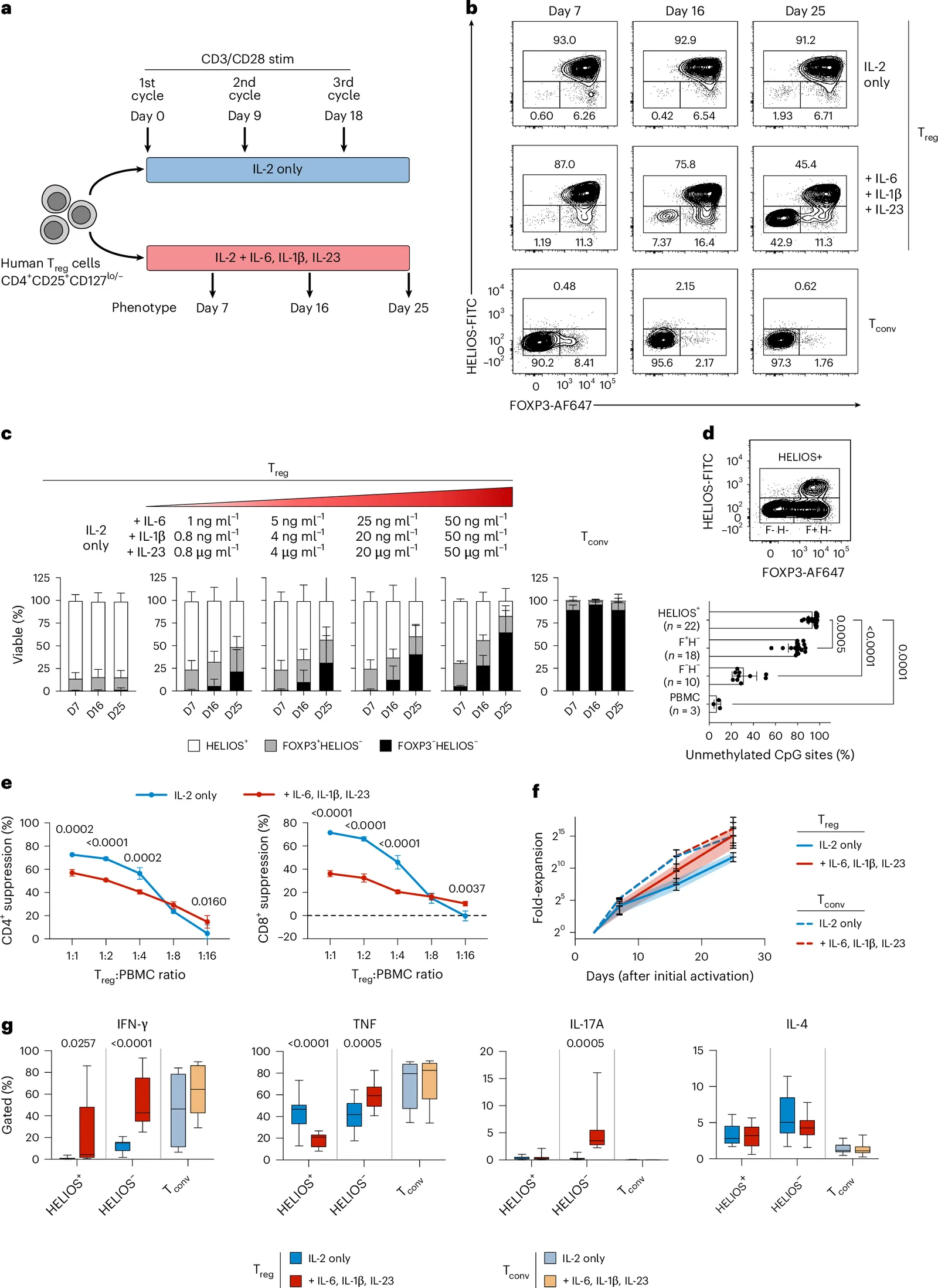
Primary human T_reg_ cells undergo progressive destabilization during repeated in vitro CD3/CD28 stimulation under sustained inflammatory cytokine exposure. **a**, Primary human CD4^+^CD25^+^CD127^lo/−^ T_reg_ cells were stimulated with anti-CD3/CD28 every 9 days in the presence or absence of IL-6, IL-1β and IL-23. The expression of FOXP3 and HELIOS was assessed at day 7 of each stimulation cycle via flow cytometry. **b**, Representative FOXP3 and HELIOS flow cytometry profiles at days 7, 16 and 25. Labels indicate the percentage of cells in the gated subpopulations for HELIOS^+^ (top), FOXP3^+^HELIOS^−^ (bottom right) and FOXP3^−^HELIOS^−^ (bottom left). Profiles of CD4^+^ T_conv_ cells are shown for comparison. **c**, T_reg_ cells were cultured as in **a** and challenged with the triple combination of IL-6, IL-1β and IL-23 across a 50-fold concentration range. Average distribution of cells within each gated subpopulation from **b** at days 7, 16 and 25 is summarized (mean ± s.d., *n* = 8 unrelated normal donors except the highest dose had five donors). **d**, Percentage of unmethylated CpG sites at the *FOXP3* CNS2 (TSDR) from each of the FACS-purified HELIOS^+^, FOXP3^+^HELIOS^−^ (F^+^H^−^) or FOXP3^−^HELIOS^−^ (F^−^H^−^) subpopulations, as determined by pyrosequencing of bisulfite-converted genomic DNA. Results shown are mean ± s.d.; the numbers of biological replicates are listed. Statistical significance was calculated via Kruskal–Wallis test with Dunn’s test. Only adjusted *P* values <0.05 are shown. **e**, After 25 days of in vitro culture, T_reg_ cells were co-cultured with PBMCs at the indicated T_reg_:PBMC ratios. In vitro suppression of responder CD4^+^ (left) and CD8^+^ (right) T cell proliferation relative to proliferation of the respective responder population in PBMC-only samples. Results shown are mean ± s.d. (*n* = 3 technical replicates). Statistical significance was calculated via two-way ANOVA and Sidak’s test. Only adjusted *P* values <0.05 are shown. **f**, Cumulative fold-expansion of T_reg_ and T_conv_ cells over 25 days of in vitro culture in the presence or absence of IL-6, IL-1β and IL-23. Results are shown as mean (lines) ± s.d. (shaded regions) (*n* = 8 unrelated normal donors). **g**, At day 25 after culturing in IL-2 only or with the addition of IL-6, IL-1β and IL-23 (25, 20 and 20 ng ml^−1^, respectively), cells were stimulated for 5 h with PMA and ionomycin and the expression of proinflammatory cytokines was assessed via intracellular staining and flow cytometry. Percentages of cells expressing IFN-γ, TNF, IL-17A and IL-4 among HELIOS^+^ cells, HELIOS^−^ T_reg_ cells and T_conv_ cells are summarized. Boxes represent the interquartile ranges, lines inside the boxes represent medians and the whiskers represent ranges from minimum to maximum (*n* = 16 unrelated normal donors). Statistical significance was calculated via repeated-measure two-way ANOVA with the Geisser–Greenhouse correction and Sidak’s test for comparisons within the same cell subsets. Only adjusted *P* values <0.05 for T_reg_ cell subsets are shown. Source data

**Extended Data Fig. 1:**
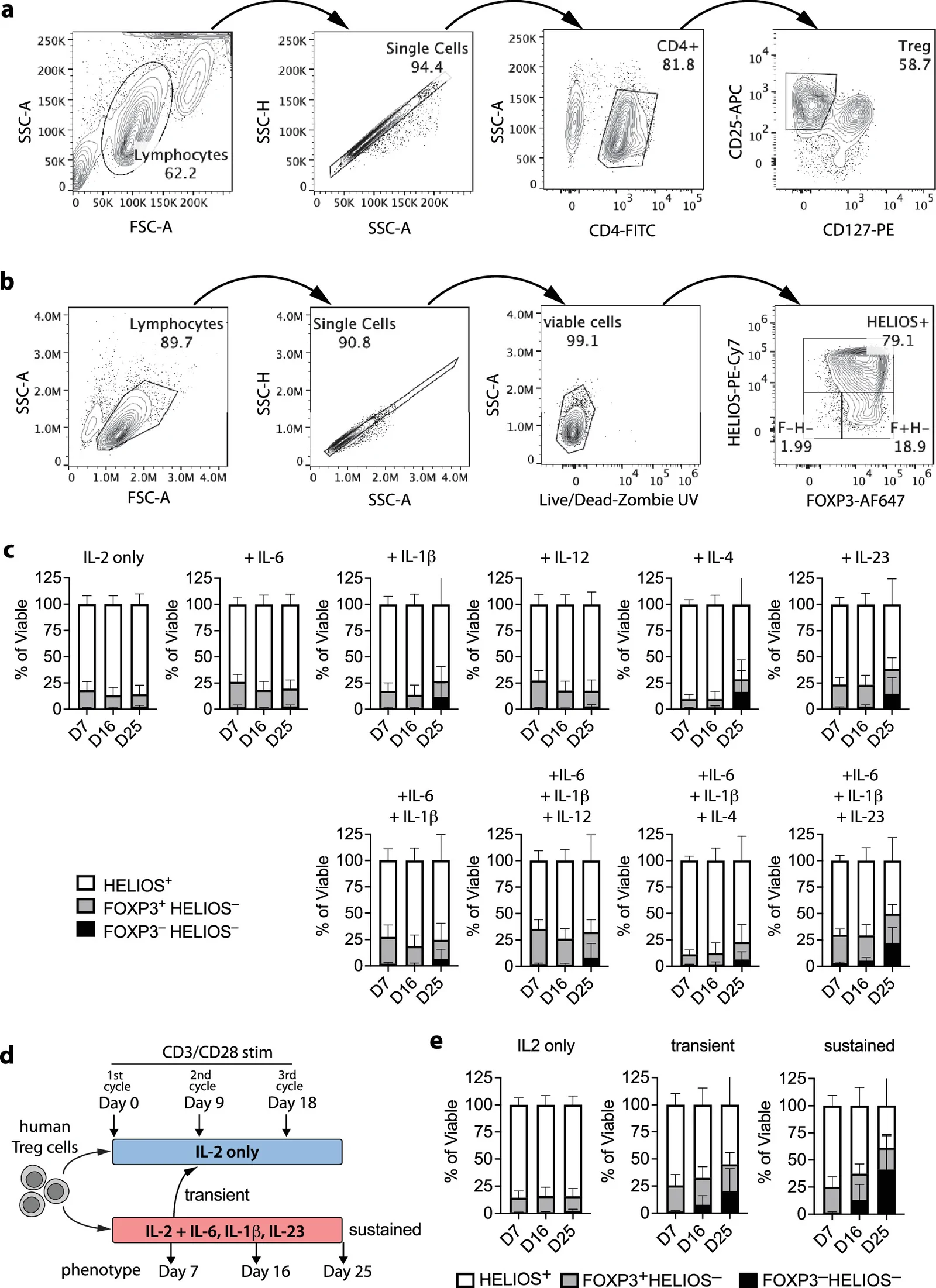
In vitro model of human Treg instability. (**a**) An example of FACS gating strategy for CD4^+^CD25^+^CD127^−/low^ Tregs from CD25 pre-enriched human PBMC. (**b**) An example of gating strategy for analytical flow cytometry of expanded human Treg purity and destabilization. (**c**) Human Tregs were expanded either with IL-2 alone or IL-2 plus the indicated cytokine(s). Average distribution of cells within the gated HELIOS^+^, FOXP3^+^HELIOS^−^, and FOXP3^−^HELIOS^−^ sub-populations at days 7, 16, and 25 of culture is summarized. Results shown are mean ± SD (n = 5 normal human donors, except IL-1β D25 had 3 samples and IL-6, IL-1β, and IL-4 D25 had 4 samples). (**d**) Human Tregs were expanded in IL-2 only or challenged with the addition of IL-6, IL-1β, and IL-23. The inflammatory cytokine cocktail was supplied either transiently for 7 days (transient) or for the duration of the 25-day culture (sustained). For the transiently challenged samples, cells were washed and subsequently expanded in IL-2 only conditions for the remainder of the culture period. (**e**) Average distribution of cells amongst HELIOS^+^, FOXP3^+^HELIOS^−^, and FOXP3^−^HELIOS^−^ populations, as determined via flow cytometry at days 7, 16, and 25 of culture is summarized. Results shown are mean ± SD (n = 8 unrelated normal donors). Source data

To investigate whether human T_reg_ cells are susceptible to losing lineage identity, CD4^+^CD25^+^CD127^lo/−^ fluorescence-activated cell sorting (FACS)-isolated primary human T_reg_ cells (Extended Data Fig. 1a) were subjected to repeated cycles of in vitro CD3/CD28 stimulation in the presence or absence of proinflammatory cytokines (Fig. 1a). As a preliminary gauge of T_reg_ cell identity, we focused on FOXP3 and HELIOS, a key pair of TFs with well-established, complementary roles in regulating T_reg_ cell fitness and suppressive function^13,14^ (Extended Data Fig. 1b). As human conventional CD4^+^ T (T_conv_) cells transiently express FOXP3 following T cell receptor (TCR) activation, expression profiles were evaluated 7 days into each stimulation cycle, after FOXP3 levels had mostly waned in non-T_reg_ cells^15^.

Previous studies on the impact of inflammatory cytokines on human T_reg_ cell lineage stability have implicated a negative role of IL-12^14,16^, whereas the effects of IL-6 have been more controversial^17–20^. We thus started with unbiased comparisons of Th1-, Th2- and Th17-driving cytokines, singly and in combinations (Extended Data Fig. 1c). We found that the Th17-promoting combination of IL-6, IL-1β and IL-23 was the most consistent at inducing FOXP3 and HELIOS silencing (Extended Data Fig. 1c). The extent of T_reg_ cell destabilization by this triple cytokine combination was dependent on both the concentrations and duration of cytokine exposure. Under prolonged exposure, primary human T_reg_ cells progressively downregulated both FOXP3 and HELIOS (Fig. 1b). The fraction of FOXP3^−^HELIOS^−^ cells scaled with the concentration of IL-6, IL-1β and IL-23 across a 50-fold range (Fig. 1c), within the range reported in inflamed tissues in humans (Supplementary Table 1). Moreover, transient exposure to the inflammatory cytokines for 7 days during the first cycle of stimulation was sufficient to drive the loss of FOXP3 and HELIOS in subsequent cycles, albeit in a smaller proportion of cells than under conditions of persistent cytokine exposure (Extended Data Fig. 1d,e). This suggested that the appearance of FOXP3^−^HELIOS^−^ cells was not due to direct repression of the expression of these genes by the cytokine cocktail but rather a more permanent change in cell identity. As T_conv_ cells rapidly out-proliferate T_reg_ cells in vitro, an increase in FOXP3^−^HELIOS^−^ cells could also indicate imperfect T_reg_ cell purity during the initial isolation. However, the very low percentages of FOXP3^−^HELIOS^−^ cells in parallel cultures without inflammatory cytokines signified high initial cell purity (Fig. 1b,c) and supported an inflammatory cytokine-dependent effect.

**Extended Data Fig. 2:**
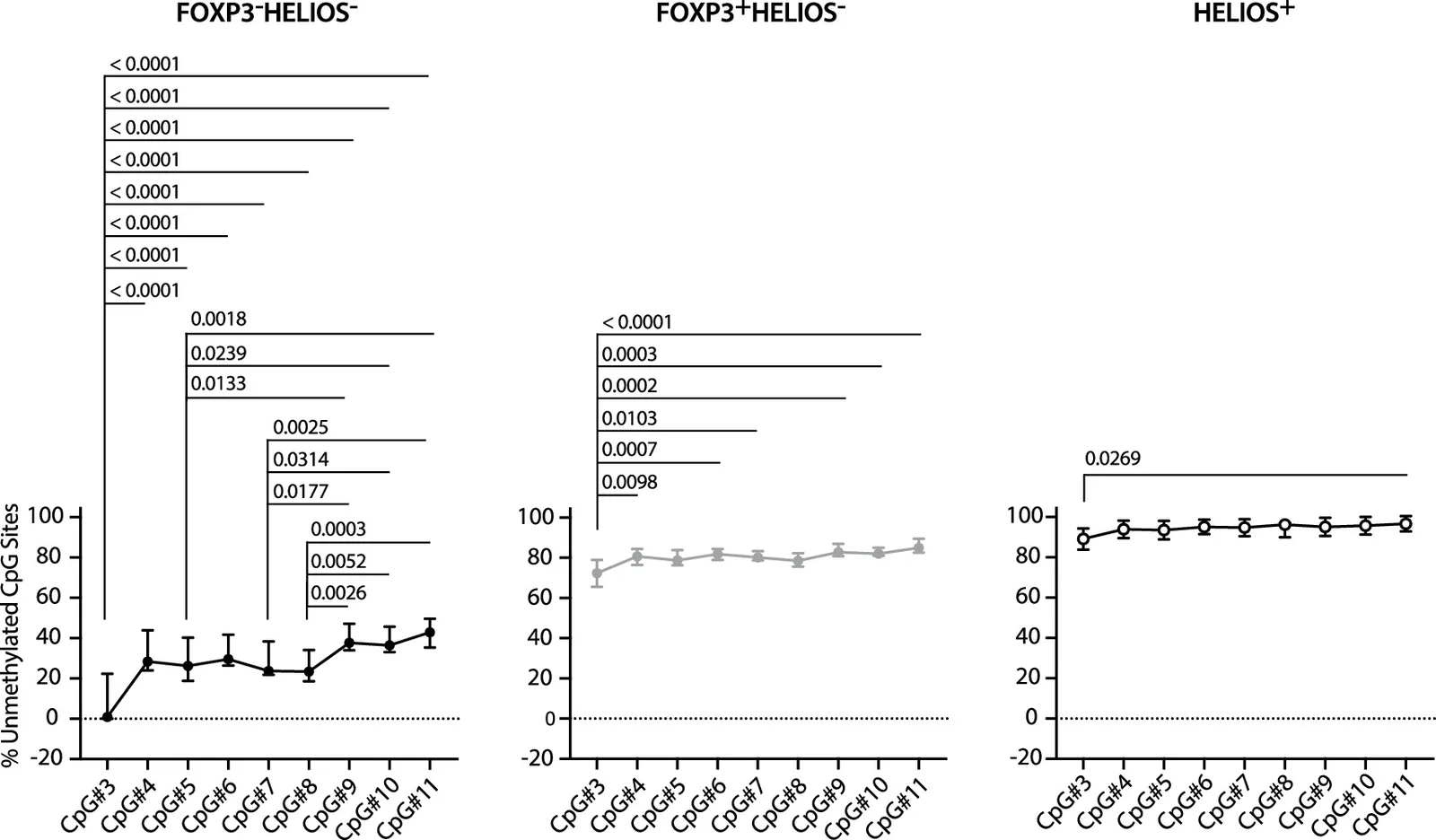
DNA re-methylation of CpG sites in FOXP3^−^HELIOS^−^ cells occurred at different rates across the TSDR. Percentage of unmethylated CpGs sites at each assayed CpG within the *FOXP3* CNS2 (TSDR) from each of the FACS-purified FOXP3^−^HELIOS^−^ (left), FOXP3^+^HELIOS^−^ (middle), or HELIOS^+^ (right) sub-populations, as determined by pyrosequencing of bisulfite-converted genomic DNA. Results shown are mean ± SD (n = 22 normal human donors for HELIOS^+^, n = 18 for F^+^H^−^, and n = 10 for F^−^H^−^). Statistical significance was calculated via two-way ANOVA and Tukey’s test. The variations among the 3 sub-populations accounted for 87.37% of the total variation (p < 0.0001) and the variation among the different CpG sites accounted for 3.647% of the total variation (p < 0.0001). Significant adjusted P values (< 0.05) for the different CpG sites within each cell subset are shown. Source data

To determine if the loss of FOXP3 and HELIOS expression reflected an epigenetically delineated change in T_reg_ cell fate, the HELIOS^+^, FOXP3^+^HELIOS^−^ and FOXP3^−^HELIOS^−^ populations were FACS purified and evaluated for DNA demethylation at the TSDR (Fig. 1d). This evolutionarily conserved *FOXP3* enhancer is uniquely demethylated in T_reg_ cells; it is fully methylated in T_conv_ cells^21,22^. The TSDR was predominantly uniformly demethylated in HELIOS^+^ cells, consistent with a highly stable, committed T_reg_ cell population. Meanwhile, FOXP3^−^HELIOS^−^ cells exhibited substantial but incomplete TSDR remethylation. To determine whether the partial methylation reflected a distinct transitional cellular state or a mixed population of demethylated T_reg_ cells and methylated non-T_reg_ cells, we examined individual CpG sites within TSDR and observed varying degrees of remethylation at different sites (Extended Data Fig. 2). This suggested that the FOXP3^−^HELIOS^−^ cells were undergoing cell state transition and provided additional evidence that this population did not expand from TSDR-methylated T_conv_ cells. Together, these data showed that patterns of HELIOS^+^ or FOXP3^−^HELIOS^−^ expression could serve as an accurate proxy for committed or destabilized T_reg_ cell identity, respectively.

**Extended Data Fig. 3:**
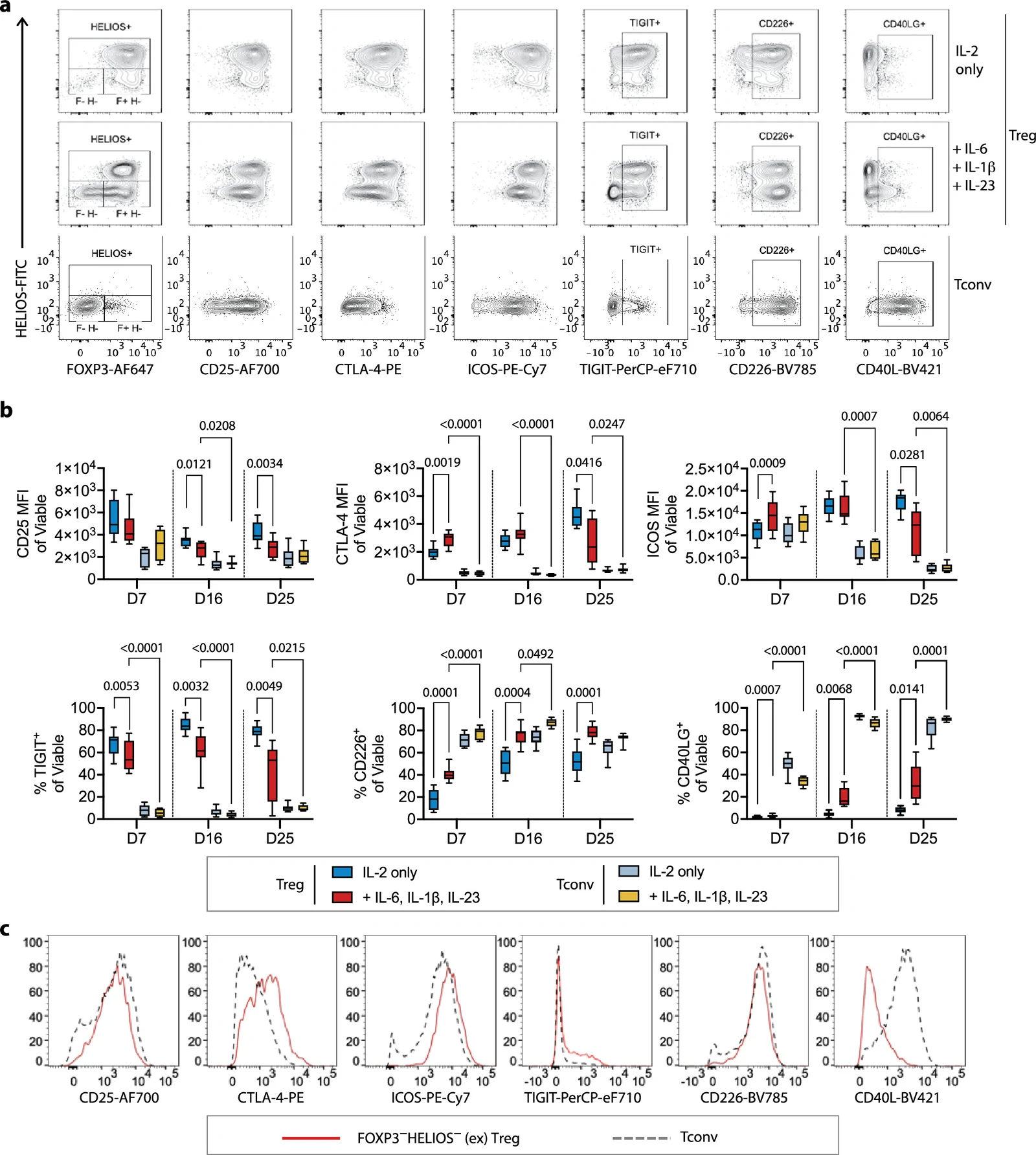
Inflammatory cytokine exposure induces dysregulated expression of Treg phenotypic markers. (**a**) Tregs were stimulated with anti-CD3/CD28 through repeated 9-day cycles either in IL-2 only or with the addition of IL-6, IL-1β, and IL-23. Representative expression profiles and gating for phenotypic markers CD25, CTLA-4, ICOS, TIGIT, CD226, and CD40LG at Day 25 are shown. (**b**) Geometric mean of fluorescence intensity (MFI; CD25, CTLA-4, ICOS) or percent positive (TIGIT, CD226, CD40LG) of phenotypic markers is summarized at days 7, 16, and 25 for each condition. Boxes represent the interquartile ranges, lines inside the boxes represent medians, and the whiskers represent ranges from minimum to maximum (n = 8 normal human donors). Statistical significance was calculated via repeated-measure two-way ANOVA with the Geisser-Greenhouse correction and Dunnette’s test for comparisons within cytokine-exposed Treg cells. Only adjusted P values less than 0.05 between cytokine-exposed Treg cells and IL-2 only Treg cells and between cytokine-exposed Treg cells and cytokine-exposed Tconv cells and are shown. (**c**) Histograms for phenotypic markers shown in (a), from the gated FOXP3^−^HELIOS^−^ population within the Treg (solid red line) and Tconv (dashed black line) samples cultured in the presence of IL-2 and IL-6, IL-1β, and IL-23. Source data

**Extended Data Fig. 4:**
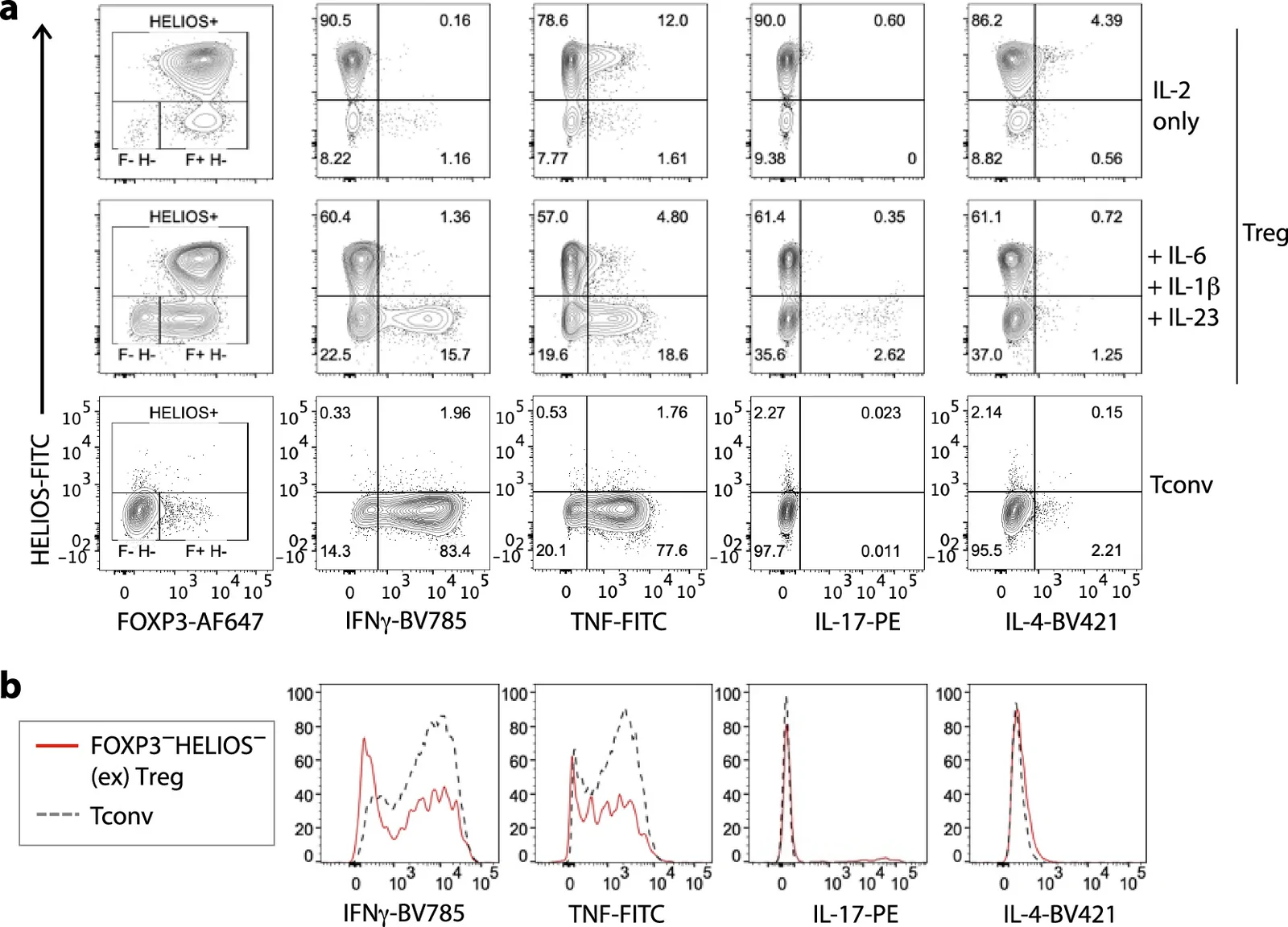
Inflammatory cytokine exposure induces production of pro-inflammatory cytokines in (ex)Treg cells. (**a**) Treg cells were stimulated with anti-CD3/CD28 through repeated 9-day cycles either in IL-2 only or in the added presence of IL-6, IL-1β, and IL-23. After 25 days of *in vitro* culture, cells were stimulated for 5 h with PMA and ionomycin prior to intracellular flow staining for pro-inflammatory cytokine expression. Representative intracellular flow staining profiles for the expression of FOXP3, HELIOS, and IFNγ, TNF, IL-17A, and IL-4. (**b**) Histograms for the intracellular cytokine staining shown in (a), from the gated FOXP3^−^HELIOS^−^ population within the Treg (solid red line) and Tconv (dashed black line) samples cultured in the presence of IL-2 and IL-6, IL-1β, and IL-23.

We next sought to determine whether the function of T_reg_ cells was affected by cytokine exposure. T_reg_ cells treated with IL-6, IL-1β and IL-23 progressively downregulated canonical T_reg_ cell markers CD25, CTLA-4, ICOS and TIGIT and upregulated CD226 and CD40LG (Extended Data Fig. 3a,b), markers that have been implicated in T_reg_ cell dysfunction^23,24^. The FOXP3^−^HELIOS^−^ population exhibited a phenotypic profile distinct from that of T_conv_ cells (Extended Data Fig. 3c), further supporting a change in cell state rather than the outgrowth of T_conv_ cell impurities from the initial isolation. Cytokine-exposed T_reg_ cells displayed reduced in vitro suppressive activity (Fig. 1e), increased cell expansion (Fig. 1f) and acquisition of IFN-γ, TNF and IL-17A production (Fig. 1g and Extended Data Fig. 4a,b), highlighting the potential of these T_reg_ cells to acquire proinflammatory functions. Collectively, these data demonstrate that human T_reg_ cells can lose lineage identity and become dysfunctional during repeated in vitro TCR stimulation in the presence of inflammatory cytokines.

### Molecular hallmarks of T_reg_ cell destabilization

**Fig. 2:**
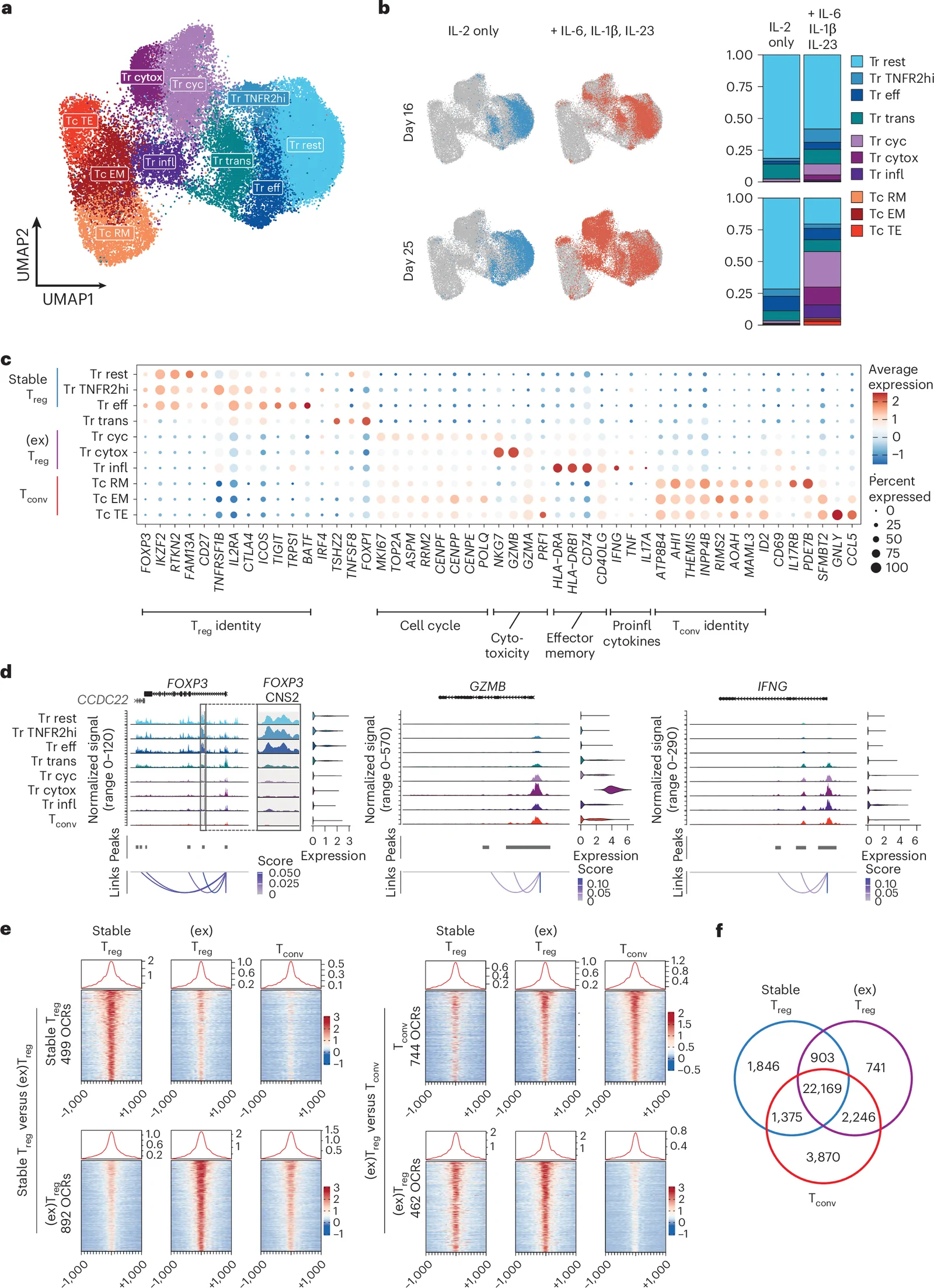
Single-cell multi-omic profiles of gene expression and chromatin accessibility reveal molecular hallmarks of T_reg_ cell lineage decommitment. **a**, Single-cell multi-omic profiles were generated from T_reg_ and T_conv_ cells isolated from three unrelated normal human donors. The cells were stimulated and cultured in the presence or absence of IL-6, IL-1β and IL-23 as shown in Fig. 1a. Cells were collected on days 16 and 25 of culture for single-cell multi-omic analysis. Unsupervised clustering on a WNN UMAP resulted in the identification of ten cell clusters shown in distinct colors. **b**, Cluster distributions for day 16 and day 25 samples from T_reg_ cells expanded in IL-2 only or IL-2 plus IL-6, IL-1β and IL-23. **c**, Transcriptional signatures of cell clusters identified in **a** are shown, with additional annotations to group phenotypically suppressive (stable) T_reg_, destabilized (ex)T_reg_ and T_conv_ cells. Proinfl., proinflammatory; RM, resident memory; EM, effector memory; TE, effector T cell. **d**, Pseudobulk chromatin accessibility tracks and corresponding gene expression violin plots are shown for each cluster at the *FOXP3* (CNS2 highlighted), *IFNG* and *GZMB* loci. ATAC-seq-defined OCRs (peaks) and correlations between peaks and transcript expression (links) are shown for each locus. **e**, Heat maps of chromatin accessibility for stable T_reg_, (ex)T_reg_ and T_conv_ cells at OCRs identified as differentially accessible between the indicated cell states. **f**, Venn diagram of shared and unique OCRs in stable T_reg_, (ex)T_reg_ and T_conv_ cells. Source data

Equipped with a robust model of human T_reg_ cell destabilization, we next set out to elucidate molecular hallmarks and key regulators of T_reg_ cell instability. Multi-omic single-cell transcriptional and chromatin accessibility profiles were generated from the nuclei of primary human T_reg_ cells actively undergoing destabilization following 16 and 25 days of IL-6, IL-1β and IL-23 exposure. Donor-matched T_reg_ cells from IL-2-only cultures and T_conv_ cells were also profiled to serve as references. Ten clusters were identified on a weighted nearest-neighbor (WNN) Uniform Manifold Approximation and Projection (UMAP) that accounted for both sequenced modalities (Fig. 2a). While there was little change in the cluster distribution between day 16 and day 25 for samples maintained in IL-2 alone, the IL-6, IL-1β and IL-23 exposure drove a subset of cells toward a progressively T_conv_ cell-like state (Fig. 2b). Inspection of cluster-defining transcriptional features revealed that the resting T_reg_ (Tr rest), effector T_reg_ (Tr eff) and TNFR2-hi T_reg_ (Tr TNFR2hi) cell clusters shared high expression of stable T_reg_ cell markers, including *FOXP3, IKZF2* (encoding *HELIOS*), *CD27, CD25, CTLA4, ICOS* and *TIGIT* (Fig. 2c). Conversely, the cycling T_reg_ (Tr cyc), cytotoxic T_reg_ (Tr cytox) and inflammatory T_reg_ (Tr infl) cell clusters all exhibited transcriptional profiles consistent with a loss of T_reg_ cell lineage identity, with downregulation of *FOXP3* and *HELIOS* and upregulation of proinflammatory cytokine and/or cytotoxicity genes, such as *IFNG*, *TNF* and *IL17A* or *NKG7*, *GZMB* and *GZMA*, respectively (Fig. 2c). We will refer to cells in these collective clusters as destabilized or putative (ex)T_reg_ cells hereafter.

Differences in gene expression often correlated with altered chromatin accessibility at the encoding locus. For example, downregulation of FOXP3 transcription in the destabilized T_reg_ cell clusters matched a loss of chromatin accessibility at the *FOXP3* CNS2 (Fig. 2d). Conversely, increased chromatin accessibility at *IFNG* and *GZMB* loci was linked to transcriptional upregulation in the destabilized T_reg_ cell clusters (Fig. 2d). These specific examples of epigenetic reprogramming mirrored a broader pattern of distinct open chromatin regions (OCRs) in destabilized T_reg_ cells, with a pronounced reduction in chromatin accessibility at stable T_reg_ cell OCRs as well as acquisition of OCRs normally inaccessible in stable T_reg_ cells (Fig. 2e). Many of these changes overlapped with differences in chromatin accessibility that distinguish T_conv_ cells from T_reg_ cells, but a subset of OCRs appeared unique to destabilized T_reg_ cells (Fig. 2f), suggesting that destabilized T_reg_ cells are still epigenetically distinct from T_conv_ cells. Together with the absence of cells in the (ex)T_reg_ and T_conv_ cell clusters that originated from IL-2-only T_reg_ cell samples, these findings further highlight that the emergence of FOXP3^−^HELIOS^−^ cells was highly unlikely to be due to outgrowth of a contaminating T_conv_ cell population.

### Chromatin alteration at the IRF4 locus in (ex)T_reg_ cells

**Fig. 3:**
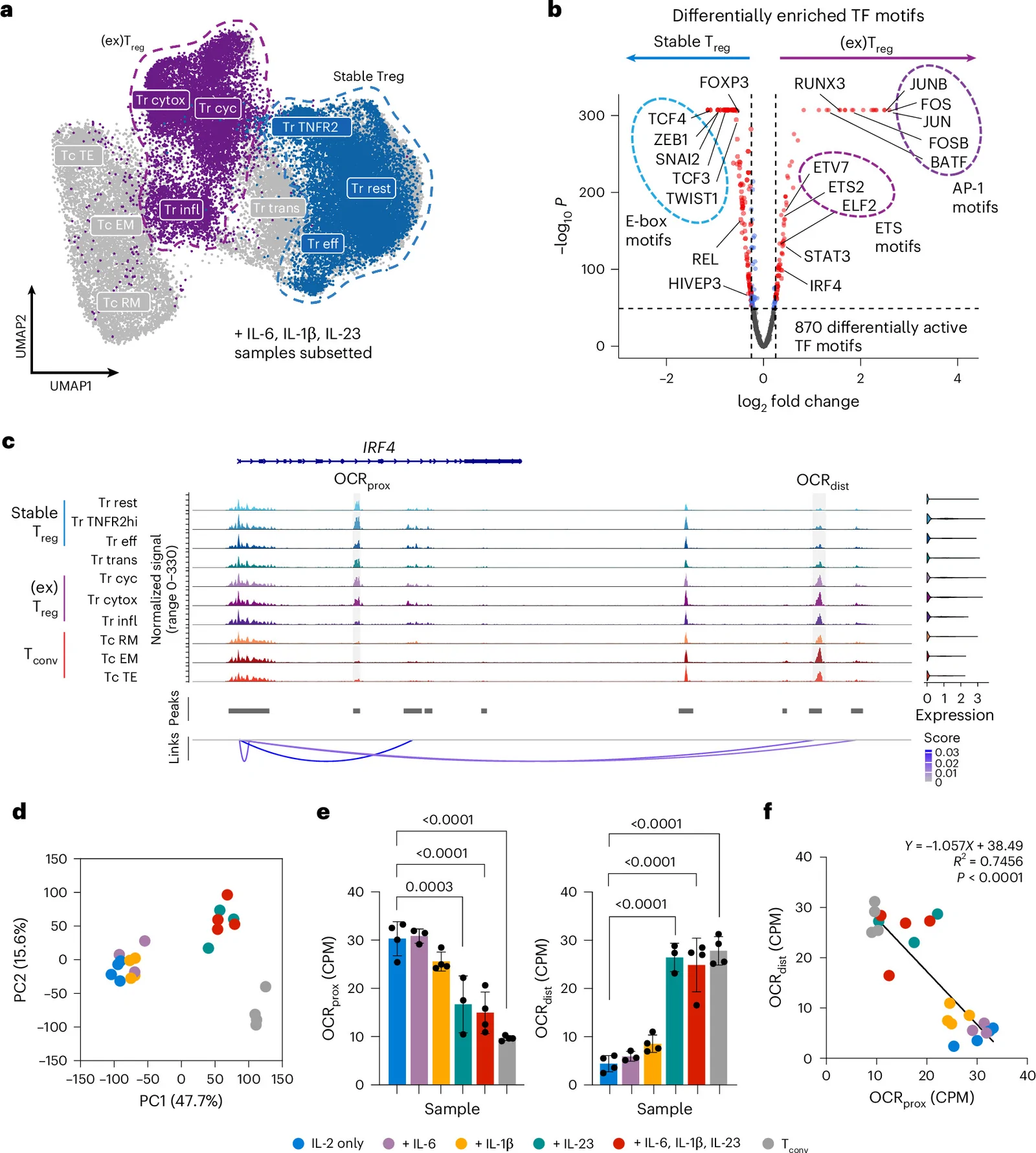
T_reg_ cells undergo a reciprocal switch in chromatin accessibility at the *IRF4* locus. **a**, TF motif enrichment analysis was performed on stable T_reg_ and (ex)T_reg_ cell clusters from IL-6, IL-1β and IL-23-treated samples isolated from three unrelated normal human donors to identify putative drivers of T_reg_ cell destabilization. **b**, Volcano plot of differentially enriched TF motifs in stable T_reg_ cells and destabilized T_reg_ cells, with the E-box, AP-1 and ETS TF families highlighted. Benjamini–Hochberg correction was used for adjusted *P* value calculation. **c**, Pseudobulk chromatin accessibility tracks and corresponding gene expression violin plots for the *IRF4* locus. The peak linkages (blue lines, bottom) indicate regions that coordinately influence gene expression. The OCR_prox_ and OCR_dist_ are highlighted in the shaded regions. **d**, Bulk ATAC-seq was performed on T_reg_ cells treated with IL-2 only or IL-2 and one or more cytokines as indicated after three rounds of TCR stimulation; the PCA plot incorporating all ATAC peaks is displayed. **e**, CPM at the ATAC peaks for OCR_dist_ and OCR_prox_ (*n* = 4 unrelated normal donors, except the IL-23 condition had three donors). Results shown are mean ± s.d., and significance was calculated via one-way ANOVA with Dunnett’s test for the IL-2-only condition. Only adjusted *P* values <0.05 are shown. **f**, Pearson correlation and simple linear regression model of OCR_dist_ and OCR_prox_ accessibility among all samples. Source data

To identify drivers of these distinct T_reg_ cell fates, we separated cells from the IL-6, IL-1β and IL-23-exposed samples into stable and (ex)T_reg_ cell states (Fig. 3a) and analyzed their OCRs for differential TF motif enrichment. Destabilized T_reg_ cells showed a relative depletion of FOXP3 and E-box elements targeted by bHLH TFs and an enrichment of AP-1, RUNX, ETS and IRF4 motifs (Fig. 3b). Intriguingly, members of the AP-1 and ETS TF families can heterodimerize with IRF4 to drive distinct transcriptional programs^25^. The enrichment of AP-1, ETS and IRF4 motifs in (ex)T_reg_ cells suggested that these TFs might cooperate to drive cell-state transition.

**Extended Data Fig. 5:**
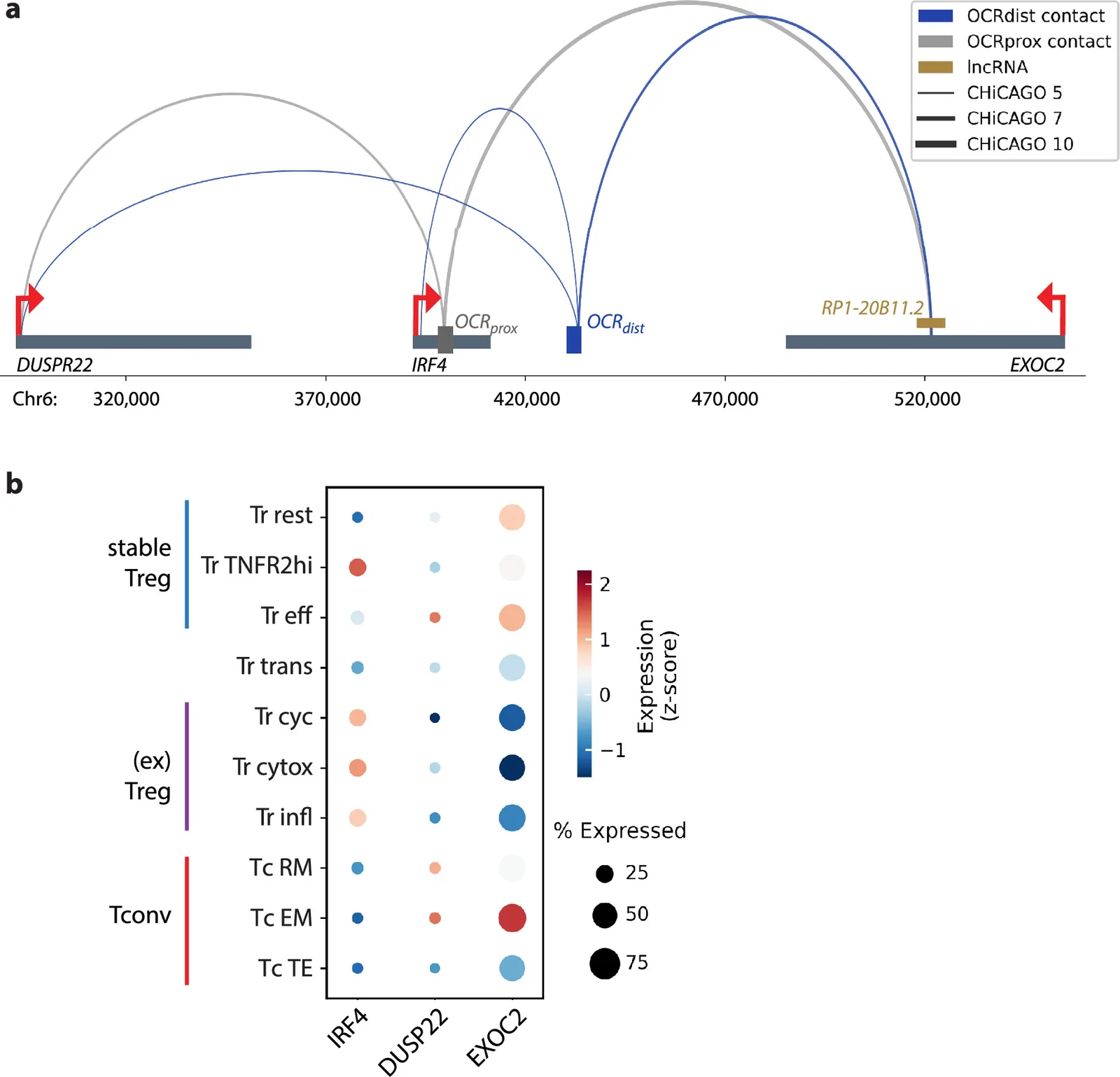
OCR_dist_ is linked to IRF4 at the chromatin level. (**a**) Analysis of promoter capture Hi-C data from ref. ^28^ in the vicinity of IRF4. Linkage significance was measured by CHiCAGO score, peak height and thickness represent higher CHICAGO score. (**b**) Expression of IRF4 and neighboring genes DUSP22 and EXOC2 in the clusters defined in Fig. 2 in the scRNAseq dataset (corresponding to Fig. 2c). Source data

Inspection of the *IRF4* locus revealed a gradual loss of chromatin accessibility at a proximal OCR (OCR_prox_) located within intron 6 (Fig. 3c). In T_reg_ cells, IRF4 expression has been reported to be FOXP3 dependent^26^, and OCR_prox_ contains a FOXP3 binding site that has been previously identified in FOXP3 chromatin immunoprecipitation followed by sequencing (ChIP-seq) experiments with both mouse and human T_reg_ cells^27^. The reduced OCR_prox_ chromatin accessibility was accompanied by a reciprocal increase in chromatin accessibility at a distal OCR (OCR_dist_) located +40 kb downstream of the IRF4 transcriptional start site (Fig. 3c). Peak-gene linkage analysis revealed that chromatin accessibility at OCR_dist_ was positively correlated with IRF4 transcript counts in our single-cell multiome dataset, hinting at potential enhancer activity and a putative enhancer switch as T_reg_ cells transitioned from a stable to destabilized state. Analysis of published promoter-capture Hi-C data in activated T cells^28^ also revealed a strong physical association between OCR_dist_ and the *IRF4* promoter, a weaker association with the promoter of *DUSP22* and no association with *EXOC2* (Extended Data Fig. 5a). *IRF4*, but not *EXOC2* or *DUSP22*, expression was positively correlated with OCR_dist_ accessibility, further supporting a regulatory role of OCR_dist_ in IRF4 expression (Extended Data Fig. 5b).

To determine the relative contribution of individual cytokines to the unraveling of the T_reg_ cell epigenome, we purified T_reg_ cells from a separate cohort of donors and exposed them to IL-6, IL-1β and IL-23 individually or in combination during three rounds of in vitro stimulation. T_conv_ and T_reg_ cells cultured in IL-2 only without proinflammatory cytokines were included as controls. Cells were collected on day 25 for bulk assay for transposase-accessible chromatin using sequencing (ATAC-seq) analyses. Principal component analysis (PCA) based on genome-wide ATAC peaks revealed that T_reg_ cells exposed to the combination of IL-6, IL-1β and IL-23 clustered closely with those exposed to IL-23 only, whereas T_reg_ cells exposed to IL-1β or IL-6 clustered with T_reg_ cells cultured in IL-2 only. Both of these clusters were separated from the T_conv_ cell cluster (Fig. 3d). This result demonstrated that IL-23 accounted for the majority of chromatin accessibility changes in the combination cytokine condition. T_reg_ cells exposed to the combination of cytokines or IL-23 only showed closure of *IRF4* OCR_prox_ and opening of *IRF4* OCR_dist_, while IL-1β and IL-6 had a minimal effect (Fig. 3e). An inverse correlation between accessibility at OCR_prox_ and OCR_dist_ among all treatment conditions was again observed (Fig. 3f). These results suggested that IL-23 had the most destabilization effect on human T_reg_ cells and the chromatin accessibility changes at *IRF4* locus reflected a genome-wide reprogramming.

### IRF4 OCR_dist_ is necessary for T_reg_ destabilization

**Fig. 4:**
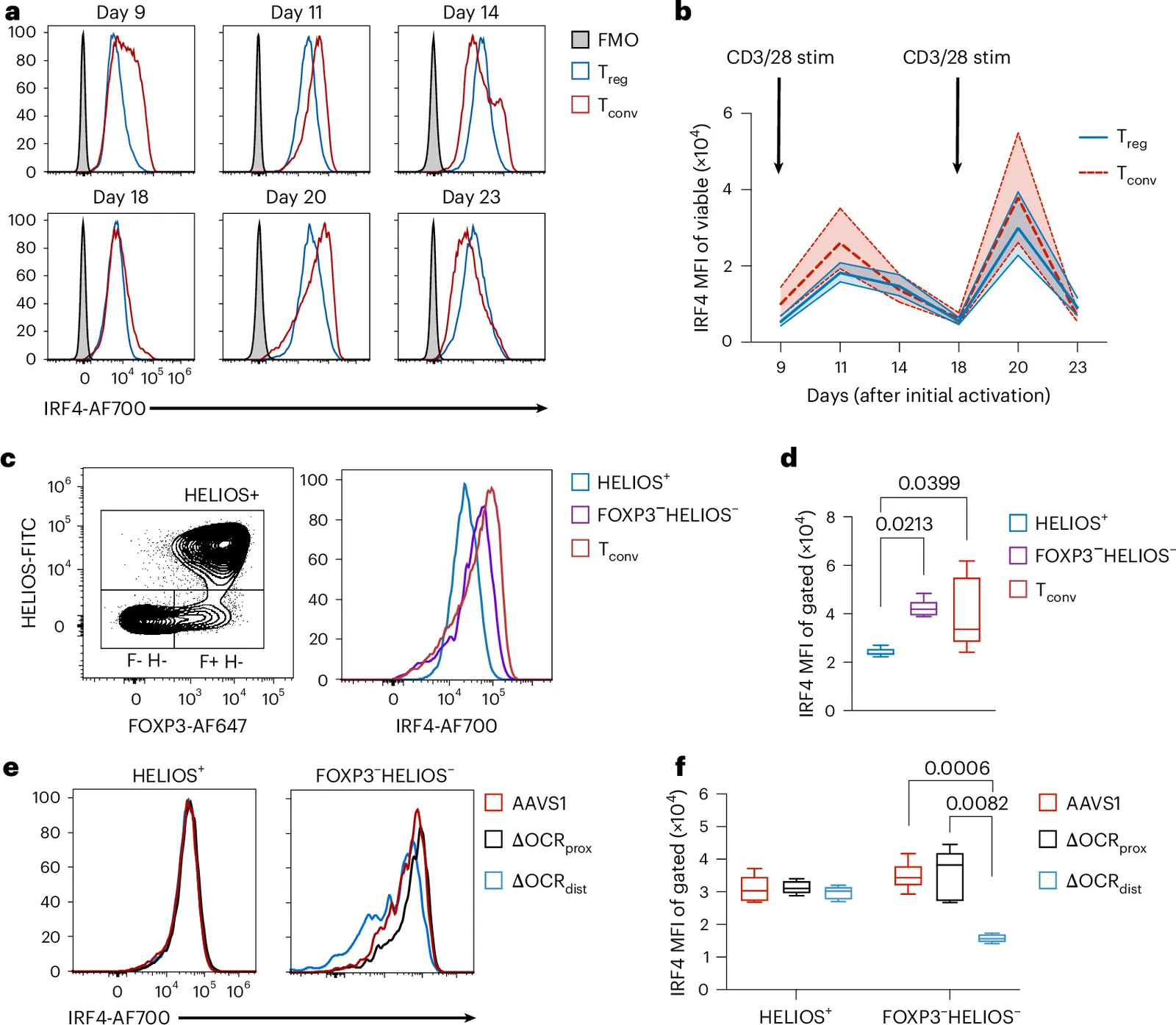
OCR_dist_ selectively promotes TCR-triggered IRF4 upregulation in destabilized FOXP3^−^HELIOS^−^ T_reg_ cells. **a**, Primary human T_reg_ and T_conv_ cells were expanded in the presence of IL-6, IL-1β and IL-23 and restimulated with anti-CD3/CD28 every 9 days. Flow cytometry histograms for IRF4 expression in each cell type at 0, 2 and 5 days of each restimulation cycle. **b**, Geometric mean fluorescence intensity (MFI) for IRF4 expression in T_reg_ and T_conv_ cells over time (*n* = 5 normal human donors) with geometric mean and geometric s.d. indicated by the lines and shaded regions, respectively. **c**, Representative HELIOS versus FOXP3 profile and histogram of IRF4 expression for HELIOS^+^ (stable) T_reg_ cells, FOXP3^−^HELIOS^−^ (destabilized) T_reg_ cells and T_conv_ cells at day 20 of culture with IL-6, IL-1β and IL-23. **d**, Geometric MFI of IRF4 expression (*n* = 5 normal human donors) for the samples in **c** is summarized. Boxes represent the interquartile ranges, lines inside the boxes represent medians and the whiskers represent ranges from minimum to maximum. Statistical significance was calculated via one-way ANOVA with Tukey’s test. **e**, CRISPR–Cas9 RNP complexes were delivered via electroporation to excise OCR_prox_ and OCR_dist_ before inflammatory cytokine exposure with IL-6, IL-1β and IL-23. Representative histograms for IRF4 expression in HELIOS^+^ (stable) and FOXP3^−^HELIOS^−^ (destabilized) subpopulations from AAVS1-edited, ΔOCR_prox_ and ΔOCR_dist_ T_reg_ cells at day 20 of in vitro culture. **f**, Geometric MFI of IRF4 expression (*n* = 6 normal human donors, except ΔOCR_prox_ had 5 donors) for the samples in **e** is summarized. Boxes represent the interquartile ranges, lines inside the boxes represent medians and the whiskers represent ranges from minimum to maximum. Significance was calculated via mixed-effects analysis with the Geisser–Greenhouse correction and Tukey’s test. Only adjusted *P* values <0.05 are shown. Source data

**Extended Data Fig. 6:**
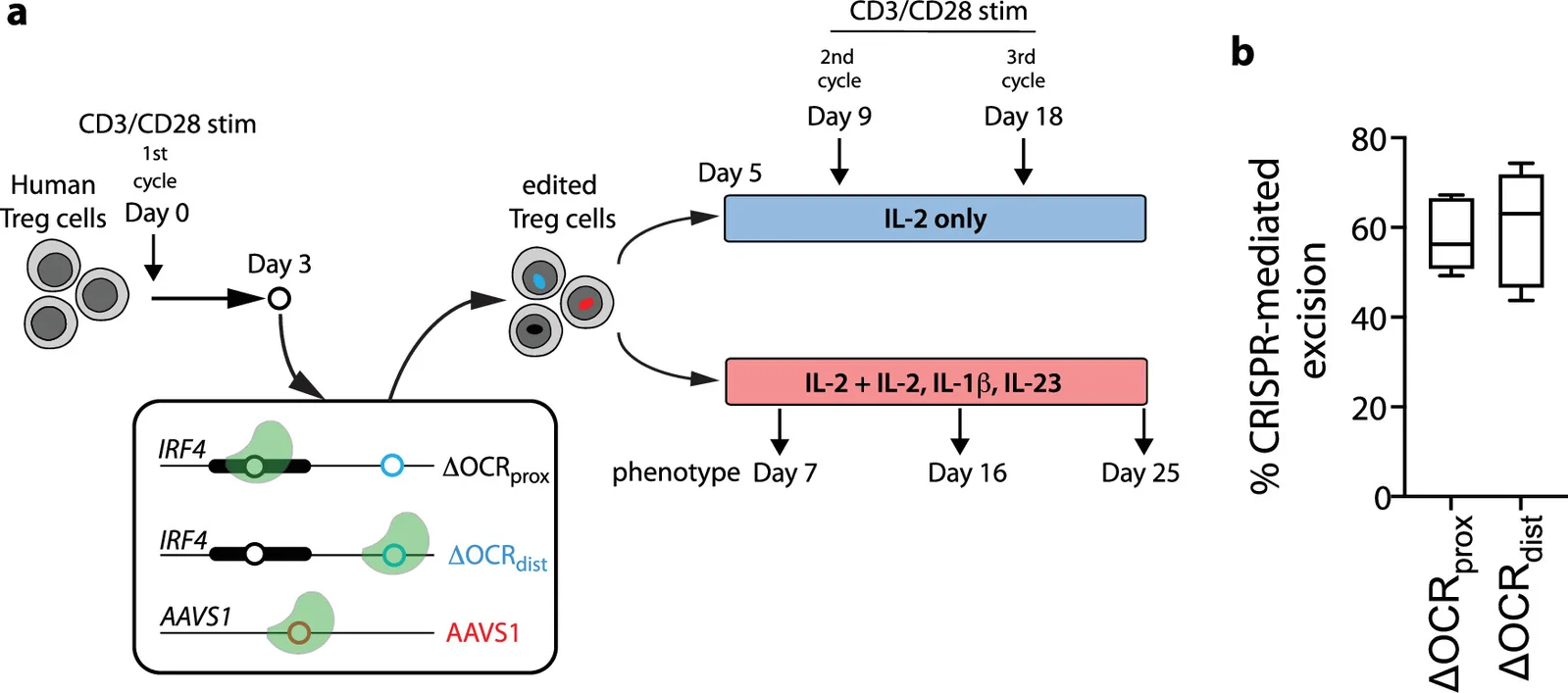
CRISPR/Cas9-mediated excision of OCR_prox_ and OCR_dist_ in primary human Tregs. (**a**) Paired CRISPR sgRNAs were designed to flank each target OCR. After initial activation in IL-2 only conditions, Tregs were treated with CRISPR/Cas9 RNPs to target AAVS1 (as a mock editing control) or to excise either OCR_prox_ or OCR_dist_. The edited cells were then split into cultures with IL-2 alone or IL-2 plus IL-6, IL-1β, and IL-23, and re-stimulated with anti-CD3/CD28 on days 9 and 18. (**b**) Each CRISPR-targeted OCR was PCR amplified from genomic DNA with primers flanking the sgRNA cut sites. The deletion efficiency for each OCR was determined as the fraction of PCR amplicons with lengths corresponding to the loss of the target region between the cut sites specified by the sgRNA pair. Boxes represent the interquartile ranges, lines inside the boxes represent medians, and the whiskers represent ranges from minimum to maximum (n = 5 normal human donors for ΔOCR_prox_ and n = 6 for ΔOCR_dist_). Source data

To determine if the chromatin changes at the *IRF4* locus impacted IRF4 expression, we first tracked IRF4 expression kinetics over the course of IL-6, IL-1β and IL-23-induced instability. IRF4 upregulation was transient in both T_reg_ and T_conv_ cells, peaking on day 2 and declining by day 5 of each anti-CD3/CD28 stimulation cycle (Fig. 4a,b). Peak IRF4 expression was higher in T_conv_ cells than in T_reg_ cells. Moreover, FOXP3^−^HELIOS^−^ cells exhibited higher peak IRF4 expression than HELIOS^+^ T_reg_ cells, indicating dysregulation of IRF4 expression in (ex)T_reg_ cells (Fig. 4c,d). To investigate the impact of OCR_prox_ and OCR_dist_ on IRF4 expression, paired single-guide RNAs (sgRNAs) flanking each OCR were designed to enable CRISPR–Cas9-mediated excision in T_reg_ cells before inducing destabilization (Extended Data Fig. 6a,b). As a CRISPR-editing control, the AAVS1 safe harbor locus was also targeted. Neither deletion of OCR_prox_ nor OCR_dist_ significantly altered IRF4 expression in HELIOS^+^ cells (Fig. 4e,f). However, IRF4 induction was significantly reduced in FOXP3^−^HELIOS^−^ cells following OCR_dist_ removal (Fig. 4e,f), suggesting that OCR_dist_ selectively supported elevated IRF4 expression in (ex)T_reg_ cells.

**Fig. 5:**
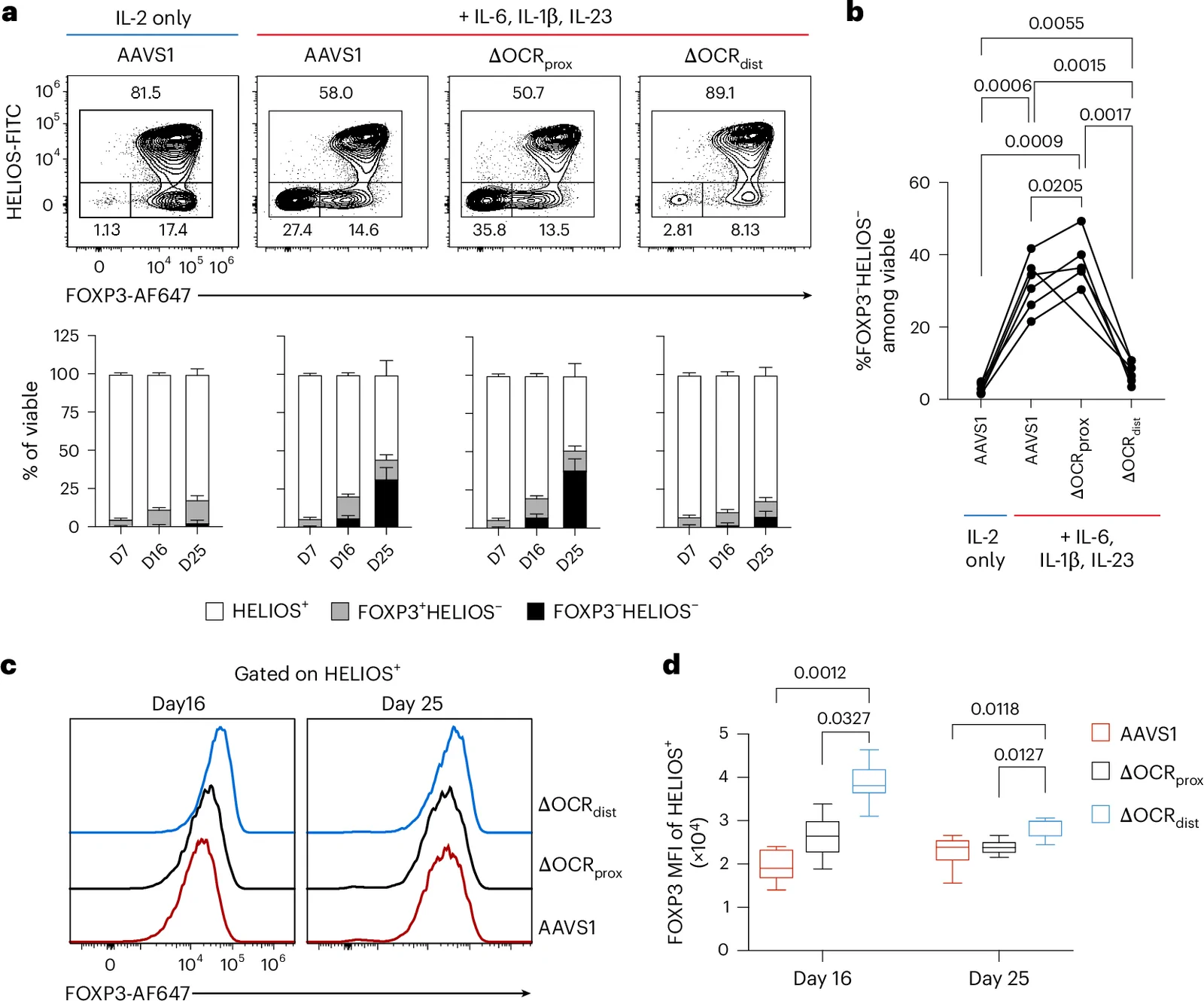
Noncoding *IRF4* regulatory elements modulate T_reg_ cell stability during inflammatory cytokine exposure. **a**, Primary human T_reg_ cells were electroporated with CRISPR–Cas9 RNPs designed to target AAVS1 or to excise either OCR_prox_ or OCR_dist_. T_reg_ cells were subsequently challenged with repeated anti-CD3/CD28 stimulation and sustained IL-6, IL-1β and IL-23 exposure to induce destabilization. Representative expression profiles for FOXP3 and HELIOS (top) and the summarized distribution among HELIOS^+^, FOXP3^+^HELIOS^−^ and FOXP3^−^HELIOS^−^ populations (bottom) at day 25 are shown as mean ± s.d. (*n* = 6 normal human donors, except ΔOCR_prox_ had 5 donors). **b**, Donor-matched comparison of the fraction of viable cells that were FOXP3^−^HELIOS^−^ cells by day 25 is summarized. **c**, Representative histograms of FOXP3 expression among the HELIOS^+^ (stable) population from AAVS1-edited, ΔOCR_prox_ and ΔOCR_dist_ T_reg_ cells at days 16 and 25 of culture with IL-6, IL-1β and IL-23 exposure are shown. **d**, Geometric MFI of FOXP3 expression in HELIOS^+^ T_reg_ cells for the samples in **c** is summarized. Boxes represent the interquartile ranges, lines inside the boxes represent medians and the whiskers represent ranges from minimum to maximum (*n* = 6 normal human donors, except ΔOCR_prox_ had 5 donors). For **b** and **d**, significance was calculated via mixed-effects analysis with the Geisser–Greenhouse correction and Tukey’s test. Only adjusted *P* values <0.05 are shown. Source data

**Extended Data Fig. 7:**
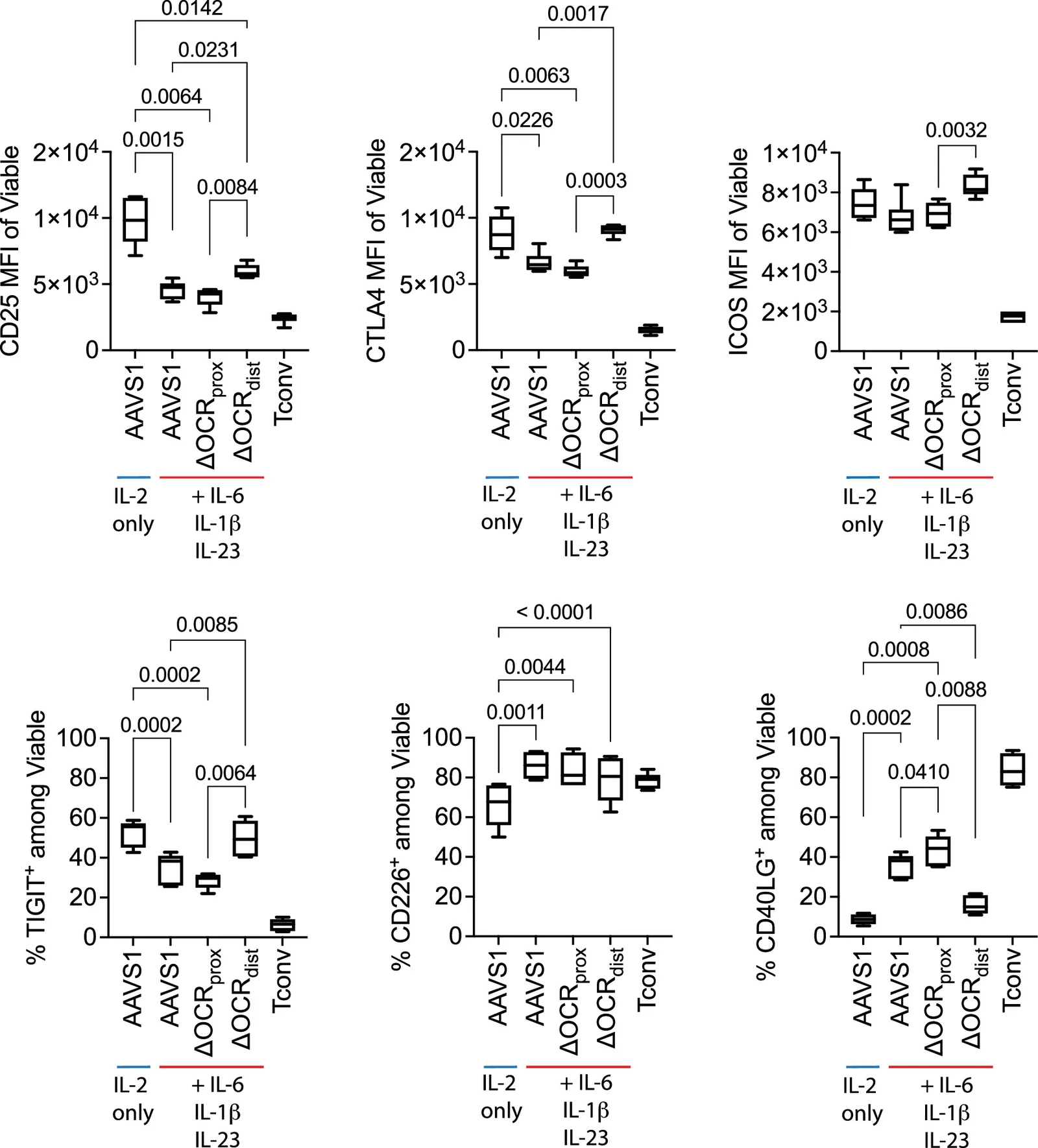
ΔOCR_dist_ Tregs retain a suppressive Treg phenotype following even after prolonged inflammatory cytokine exposure. Primary human Tregs were edited with CRIPSR/Cas9 RNPs and subsequently challenged with repeated anti-CD3/CD28 stimulation in the presence or absence of IL-6, IL-1β, and IL-23 to induce destabilization. Average geometric mean of fluorescence intensity (CD25, CTLA-4, ICOS) or percent positive (TIGIT, CD226, CD40LG) of common Treg phenotypic markers at day 25. Data are summarized. Boxes represent the interquartile ranges, lines inside the boxes represent medians, and the whiskers represent ranges from minimum to maximum (n = 6 normal human donors, except for ΔOCR_prox_ that had 5 donors). Statistical significance was calculated via mixed-effects analysis with the Geisser-Greenhouse correction and Tukey’s test. Only adjusted P values less than 0.05 are shown. Source data

Despite not having a significant effect on IRF4 expression, excision of OCR_prox_ mildly exacerbated IL-6, IL-1β and IL-23-induced T_reg_ cell destabilization (Fig. 5a), leading to a 30% increase in FOXP3^−^HELIOS^−^ cells by day 25 relative to donor-matched AAVS1-edited controls (Fig. 5b). This suggests that OCR_prox_ has a protective role in maintaining T_reg_ cell identity, although this regulation appears to occur through an undeciphered, indirect mechanism rather than by acting as a direct *IRF4* enhancer. Meanwhile, ΔOCR_dist_ T_reg_ cells retained a remarkably high degree of lineage stability (Fig. 5a), with an 85% reduction in the proportion of FOXP3^−^HELIOS^−^ cells over the course of prolonged inflammatory cytokine exposure when compared with donor-matched AAVS1-edited controls (Fig. 5b). ΔOCR_dist_ T_reg_ cells had higher expression of T_reg_ cell-associated markers CD25, CTLA-4, ICOS and TIGIT and decreased expression of CD40L (Extended Data Fig. 7). Comparison of ΔOCR_dist_ T_reg_ cells to AAVS1-edited counterparts under destabilizing conditions revealed that OCR_dist_ excision resulted in elevated FOXP3 expression in stable HELIOS^+^ T_reg_ cells (Fig. 5c,d), suggesting the amount of IRF4 in T_reg_ cells may control T_reg_ cell stability by modulating FOXP3 expression.

### IRF4 and AP-1 cooperate to drive T_reg_ cell destabilization

**Fig. 6:**
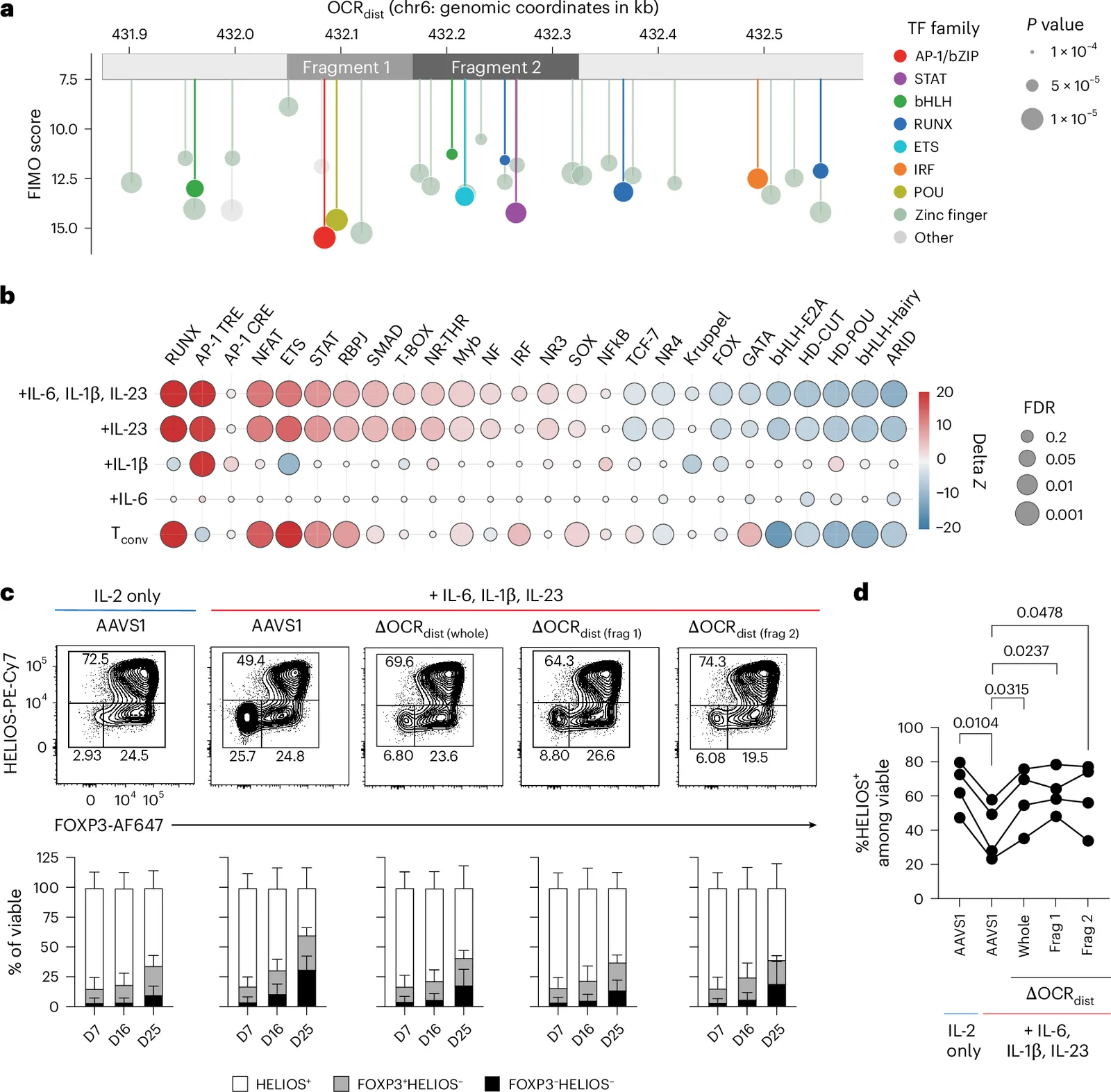
T_reg_ cells treated with inflammatory cytokines exhibit altered TF binding site accessibility, including sites found inside *IRF4* OCR_dist_. **a**, The OCR_dist_ region was scanned for TF binding sites found in the JASPAR 2026 database using FIMO (Benjamini–Hochberg-adjusted *P* value <1 × 10^−4^). The 90 identified sites were classified by family and merged into 33 family-level binding sites, which are displayed. The FIMO score is indicated by stem length, and the *P* value is indicated by bubble size. **b**, T_reg_ cells were treated with IL-2 plus the indicated cytokines for three rounds of stimulation, and bulk ATAC-seq was performed. TF family motifs were curated from JASPAR 2026, and per-sample chromVAR *z*-scores were computed for the union peak set of each TF family. Significance was calculated using limma with Benjamini–Hochberg multiple testing correction. All comparisons are relative to the IL-2-only condition. **c**, Primary human T_reg_ cells (*n* = 4 normal human donors) were electroporated with CRISPR–Cas9 RNPs designed to target AAVS1 or to excise OCR_dist_ (either whole, fragment 1 or fragment 2). T_reg_ cells were subsequently challenged with repeated anti-CD3/CD28 stimulation and sustained IL-6, IL-1β and IL-23 exposure to induce destabilization. Representative expression profiles for FOXP3 and HELIOS (top) and the summarized average distribution among HELIOS^+^, FOXP3^+^HELIOS^−^ and FOXP3^−^HELIOS^−^ populations (bottom) at day 25 are shown as mean ± s.d. **d**, Donor-matched comparison of the fraction of viable cells that remained HELIOS^+^ by day 25 is summarized as mean ± s.d. Statistical significance was calculated via repeated-measure one-way ANOVA with Geisser–Greenhouse correction and Tukey’s test. Only adjusted *P* values <0.05 are shown. Source data

**Extended Data Fig. 8:**
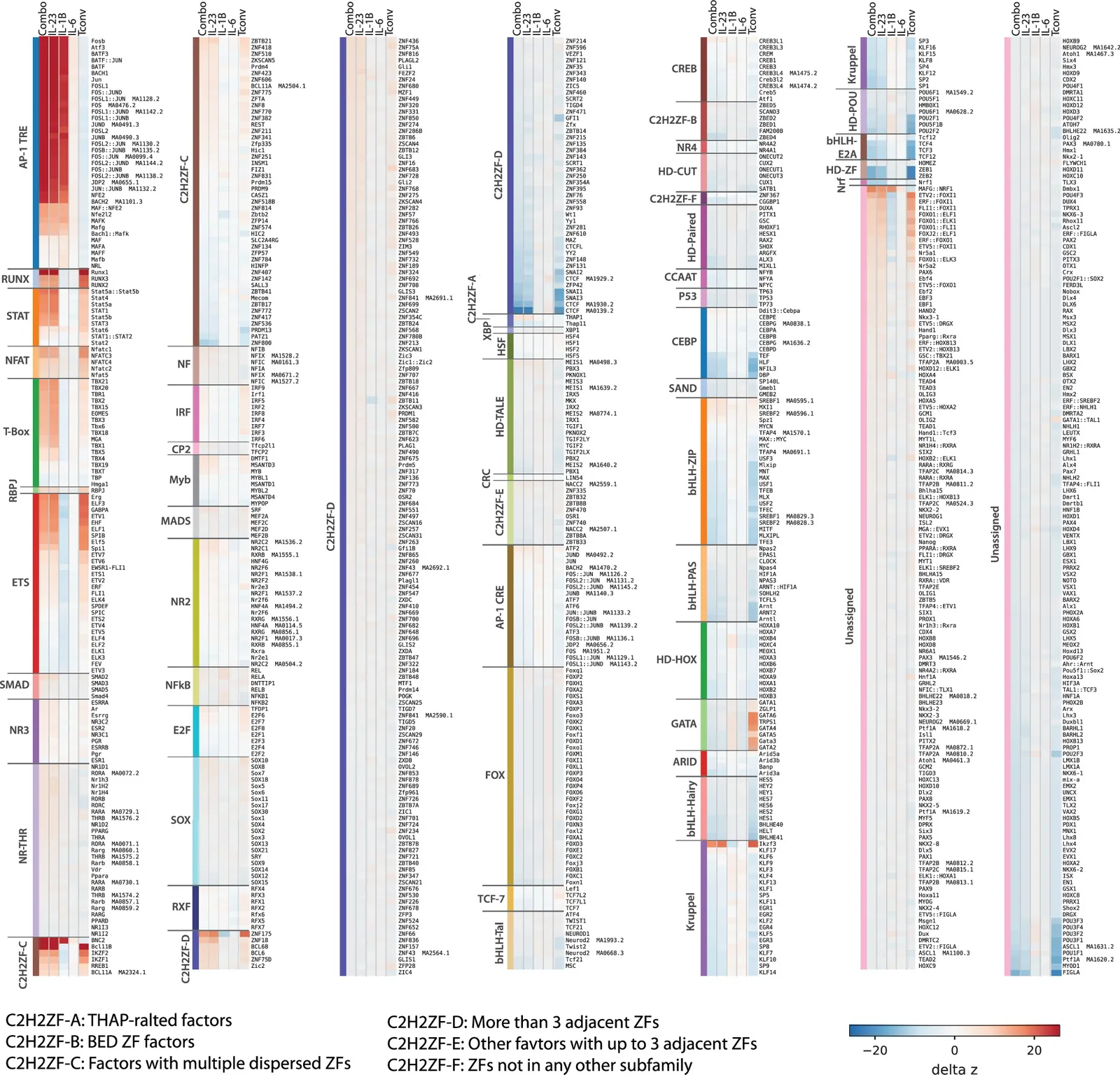
Motif-level TF binding site accessibility changes in Tregs after cytokine treatment. Tregs were treated with IL-2 plus the indicated cytokines for 3 rounds of stimulation, and bulk ATAC-seq was performed. TF family motifs were curated from JASPAR 2026, and per-sample chromVAR z-scores were computed for each TF motif. Significance was calculated using limma with Benjamini-Hochberg multiple testing correction. All comparisons are relative to the IL-2-only condition (corresponding to Fig. 6b). Source data

OCR_dist_ contained 33 TF motifs (Fig. 6a) targeted by AP-1, ETS, STAT, RUNX3, IRF and members of several zinc-finger family TFs. The predicted AP-1, ETS and STAT binding sites were near the center of the ATAC-seq peak (~280 base pairs (bp), ~360 bp and ~400 bp from the N terminus, respectively), which suggested that occupancy by these TFs might dictate OCR_dist_ chromatin accessibility. Analysis of genome-wide TF motif accessibility revealed that the canonical AP-1 motif, TPA response element (TRE), was the most significantly enriched in T_reg_ cells exposed to inflammatory cytokines compared with stable T_reg_ cells cultured with IL-2 alone but was not enriched in T_conv_ cells (Fig. 6b). By contrast, RUNX, STAT and ETS motifs were enriched in accessible chromatin in both T_conv_ cells and cytokine-exposed T_reg_ cells, suggesting that the opening of these TF sites may be a late event during T_reg_ cell destabilization or a consequence rather than an initiator of T_reg_ cell lineage loss. IL-23 alone altered TF site accessibly similarly to that seen in combination cytokine-treated T_reg_ cells. By contrast, IL-1β alone significantly increased AP-1 accessibility but significantly decreased accessibility of ETS sites, whereas IL-6 alone minimally changed accessibility to any TF sites profiled (Fig. 6b and Extended Data Fig. 8). Notably, GATA TF sites remained inaccessible in cytokine-treated T_reg_ cells, distinct from T_conv_ cells, providing further evidence of destabilization rather than expansion of contaminant T_conv_ cells.

These results suggested that the central region in *IRF4* ORC_dist_ containing the AP-1, ETS and STAT motifs might be important for driving T_reg_ cell destabilization. We then designed additional sgRNAs to excise fragment 1 containing the AP-1 motif or fragment 2 containing ETS and STAT motifs (Fig. 6a) and subjected the edited T_reg_ cells to repeated stimulation in the presence IL-6, IL-1β and IL-23. Deleting either of these fragments individually in T_reg_ cells conferred protection to a similar extent than the deletion of the entire OCR_dist_ (Fig. 6c,d), suggesting that both AP-1 and ETS or STAT binding sites in OCR_dist_ are necessary for cytokine-induced T_reg_ cell destabilization.

**Fig. 7:**
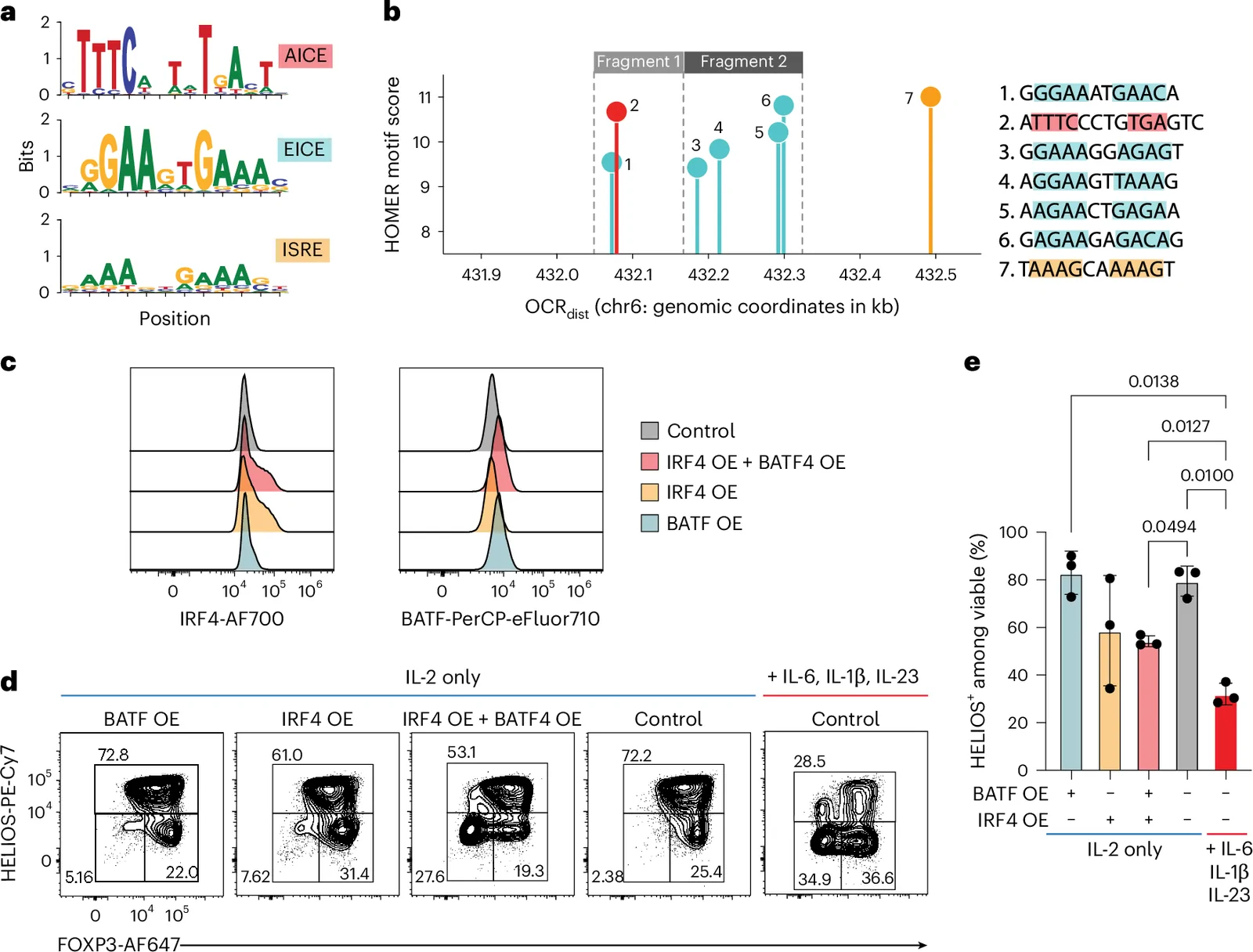
Core TFs with binding sites found in OCR_dist_ drive cytokine-induced T_reg_ cell destabilization. **a**, Motifs for AICE, EICE and ISRE. **b**, Summary of AICE, EICE and ISRE elements found within IRF4 OCR_dist_ using HOMER. **c**–**e**, Primary human T_reg_ cells (*n* = 3 normal human donors) were treated with lentivirus containing CRISPRa machinery and an sgRNA targeting either BATF, IRF4, a combination of these two sgRNAs or a nontargeting control sgRNA. OE, overexpression. Transduced cells were sorted and T_reg_ cells were subsequently challenged with repeated anti-CD3/CD28 stimulation with IL-2 only (except for positive control treated with IL-6, IL-1β and IL-23). Representative histograms with geometric MFI for BATF, IRF4 on day 24 (**c**) and representative plots with gated proportions for FOXP3/HELIOS on day 38 (**d**) are shown. Donor-matched comparison of the fraction of viable cells that remained HELIOS+ by day 38 is summarized as mean ± s.d. (*n* = 3 normal human donors) (**e**). Statistical significance was calculated via repeated-measure one-way ANOVA with the Geisser–Greenhouse correction and Tukey’s test. Only adjusted *P* values <0.05 are shown. Source data

Our data thus far showed that proinflammatory cytokines increased accessibility to AP-1, ETS and STAT binding sites and altered chromatin landscape in T_reg_ cells. Moreover, *IRF4* OCR_dist_ accessibility, particularly the region containing AP-1, ETS and STAT binding sites, was necessary for T_reg_ cell destabilization, probably through increasing IRF4 expression. This raised the question of whether IRF4 was responsible for reprograming the T_reg_ cell fate. Notably, IRF family-level accessibility was not significantly altered by cytokine exposure (Fig. 6b). While IRF4 can function as a homodimer by binding to interferon-stimulated response elements (ISRE), it can also be recruited to AP-1–IRF composite elements (AICEs) and ETS–IRF composite elements (EICEs) (Fig. 7a). We analyzed OCR_dist_ and identified one AICE, one ISRE and five EICE sites (Fig. 7b), suggesting that IRF4 may enhance its own expression via partnership with AP-1 and ETS TFs at OCR_dist_. This constitutes a feedforward mechanism to reinforce OCR_dist_ accessibility and sustain high IRF4 expression, implying that the opening of *IRF4* OCR_dist_ might be a point of no return in T_reg_ cell lineage decommitment.

To test our hypothesis that increased abundance of AP-1 and IRF4 in the presence of cytokines drove T_reg_ cell destabilization, we used a CRISPRa system to overexpress IRF4 and the AP-1 family member BATF alone or in combination and assessed the impact on T_reg_ cell stability during repeated stimulation without inflammatory cytokines. We chose BATF because it is known to partner with IRF4 to form a high-affinity binding complex at AICE sites^29,30^. T_reg_ cells dually transduced with lentiviral vectors containing CRISPRa and sgRNA targeting IRF4 and/or BATF were FACS purified and validated for overexpression by flow cytometry (Fig. 7c). At the end of the fourth stimulation cycle, the proportion of HELIOS^+^ cells was unchanged by BATF overexpression alone, variably decreased by IRF4 overexpression and consistently and significantly decreased by dual overexpression of IRF4 and BATF (Fig. 7d,e). Thus, the effect of cytokine-driven T_reg_ cell destabilization could be partially recapitulated via overexpression of both IRF4 and BATF, supporting a model of IRF4 and AP-1 partnership as a potent driver of T_reg_ cell destabilization.

### Deletion of IRF4 OCR_dist_ preserves T_reg_ cell function

**Fig. 8:**
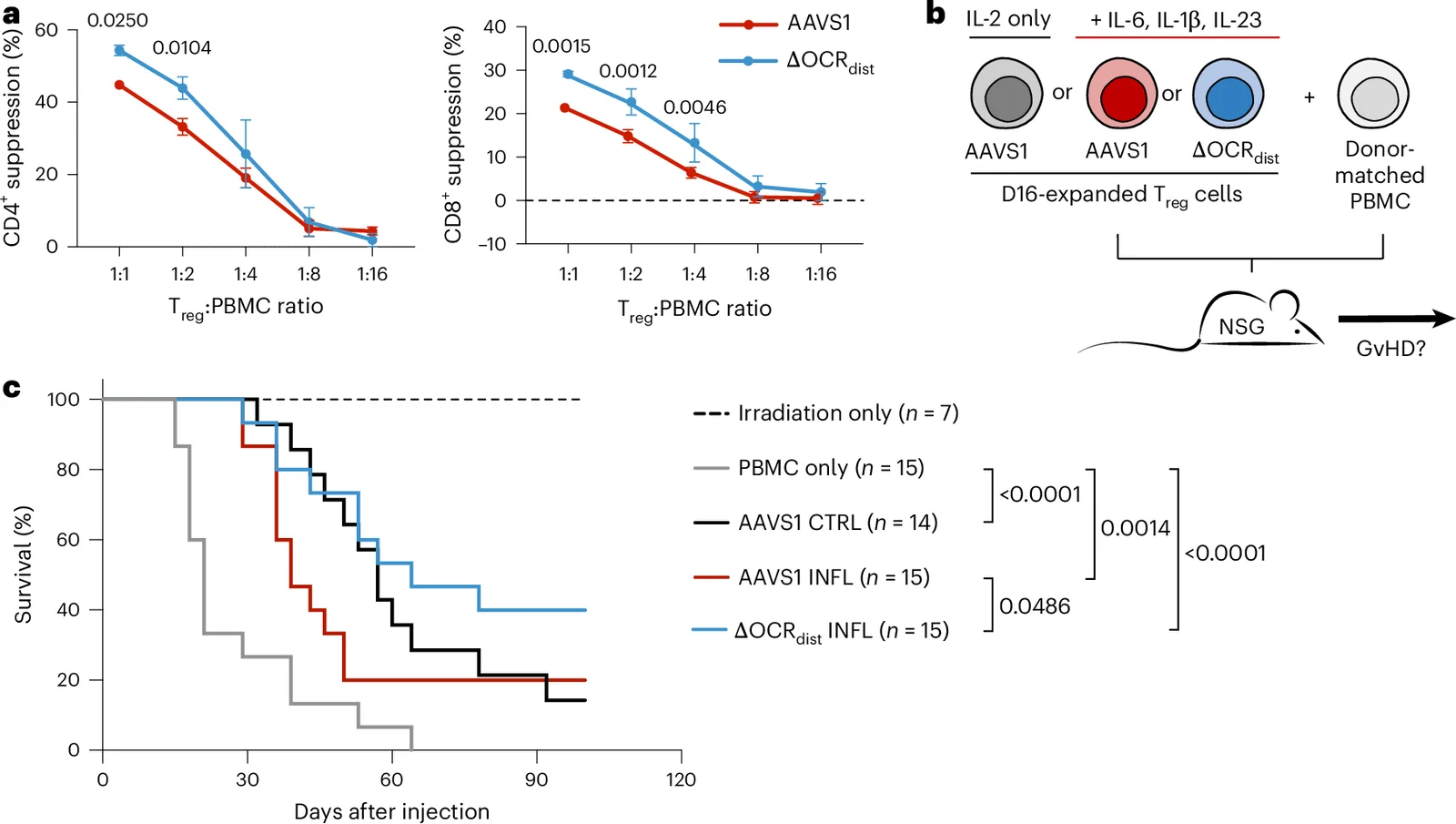
ΔOCR_dist_ T_reg_ cells exhibit greater in vitro suppressive activity and mediate greater protection from xenogeneic GvHD. **a**, AAVS1 and ΔOCR_dist_ CRISPR-edited primary human T_reg_ cells were challenged with repeated anti-CD3/CD28 stimulation cycles under IL-6, IL-1β and IL-23 exposure to induce destabilization. At day 25, the resulting cells were co-cultured with PBMCs at the specified T_reg_:PBMC ratios. Percent in vitro suppression of responder CD4^+^ (left) and CD8^+^ (right) T cell proliferation relative to the respective responder populations in PBMC-only (no T_reg_) controls are shown as mean ± s.d. (*n* = 3 technical replicates). Significance was calculated via ordinary two-way ANOVA and Sidak’s test. Only adjusted *P* values <0.05 are shown. **b**, AAVS1 and ΔOCR_dist_ CRISPR-edited primary human T_reg_ cells (*n* = 2 normal donors) were expanded in the presence or absence of IL-6, IL-1β and IL-23 for 16 days to sensitize the T_reg_ cells to inflammatory cytokine-induced instability. After 16 days of in vitro expansion, T_reg_ cells and donor-matched PBMCs were co-injected into pre-irradiated NSG mice at 1:1 T_reg_:PBMC ratio. Mice weighed twice weekly and monitored for signs of GvHD. **c**, Survival curve for irradiation only (no cells injected, *n* = 7 mice), PBMC only (no T_reg_ cells, *n* = 15 mice, 7 or 8 per PBMC donor), AAVS1-edited expanded in IL-2-only conditions (AAVS1 CTRL, *n* = 14 mice, 6 or 8 per donor) and inflammatory cytokine-challenged AAVS1-edited (AAVS1 INFL, *n* = 15 mice, 7 or 8 per PBMC donor) and ΔOCR_dist_ (ΔOCR_dist_ INFL, *n* = 15 mice, 7 or 8 per PBMC donor) groups. Data are pooled from an experiment using two normal human donors. Statistical significance was determined via Gehan–Brewslow–Wilcoxon test. Only *P* values <0.05 are shown. Source data

The marked preservation of T_reg_ cell identity by OCR_dist_ deletion has the potential to enhance both the clinical safety and efficacy of engineered T_reg_ cell therapies. Unlike AAVS1-edited T_reg_ cells, which downregulated CD25, CTLA-4, ICOS and TIGIT over the course of sustained IL-6, IL-1β and IL-23 exposure, ΔOCR_dist_ T_reg_ cells maintained a suppressive phenotype (Extended Data Fig. 7). Accordingly, ΔOCR_dist_ T_reg_ cells were more effective than AAVS1-edited T_reg_ cells at suppressing the proliferation of responder CD4^+^ and CD8^+^ T cells in a co-culture assay (Fig. 8a). To determine whether the superior in vitro suppressive activity of ΔOCR_dist_ T_reg_ cells would translate to enhanced in vivo function, we adapted an established model for T_reg_ cell-mediated protection of xenogeneic graft-versus-host disease (GvHD), in which ex vivo-expanded T_reg_ cells and donor-matched peripheral blood mononuclear cells (PBMCs) are co-injected into NOD.Cg-Prkdc^scid^ Il2rg^tm1Wjl^/SzJ (NSG) mice.

As the human myeloid cells primarily responsible for producing IL-6, IL-1β and IL-23 do not engraft efficiently in NSG mice, we presensitized AAVS1-edited and ΔOCR_dist_ T_reg_ cells with 16 days of in vitro inflammatory cytokine exposure before cell injection (Fig. 8b). This pretreatment was designed to force T_reg_ cells to the threshold of destabilization, thereby enabling a more accurate assessment of the functional impairment induced by T_reg_ cell instability. To gauge the activity of non-sensitized T_reg_ cells, a group of AAVS1-edited T_reg_ cells was also expanded in the absence of cytokines (Fig. 8b). All three groups of T_reg_ cells significantly prolonged mouse survival compared with the PBMC-only group, but the function of AAVS1-edited T_reg_ cells prechallenged with IL-6, IL-1β and IL-23 was impaired relative to untreated T_reg_ cells (Fig. 8c). ΔOCR_dist_ T_reg_ cells, despite having undergone inflammatory cytokine exposure before injection, were able to prolong survival equivalently to or slightly better than AAVS1-edited T_reg_ cells expanded in the absence of cytokines. Collectively, these results demonstrate that engineered ΔOCR_dist_ T_reg_ cells acquire greater resistance to inflammatory cytokine-induced destabilization as well as increased suppressive potency in vitro and in vivo, presenting a strategy for generating safer and more effective adoptive T_reg_ cell therapies.

## Discussion

In this study, we developed an in vitro model of cytokine-induced human T_reg_ cell destabilization. Under normal in vitro expansion conditions with IL-2 alone, CD4^+^CD25^+^CD127^lo/−^ FACS-purified primary human T_reg_ cells maintained lineage identity during repeated stimulations. Destabilization was reliably induced, and the severity was influenced by the cycles of TCR stimulation as well as the dose and duration of inflammatory cytokine exposure. These results highlight the remarkable lineage stability of human T_reg_ cells as well as their vulnerability to inflammatory cytokine-induced reprogramming. This in vitro model of human T_reg_ cell instability is yet to be tested in vivo. Tracking T_reg_ cell TCR clonotypes^7^ and epigenome over time in autoimmune versus normal controls may shed light on whether chronic inflammation contributes to human T_reg_ cell lineage instability in vivo. The molecular signatures of human T_reg_ cell lineage decommitment identified in this study may aid future investigations.

Our investigation into the molecular programs that contribute to T_reg_ cell lineage decommitment revealed a striking dichotomy in the apparent use of two noncoding regions at the *IRF4* locus. Neither OCR_prox_ nor OCR_dist_ have been previously reported to regulate IRF4 expression or T_reg_ cell identity. IRF4 expression in human T_reg_ cells was lower when compared with T_conv_ cells after TCR activation, and this difference reflected differential access to the *IRF4* OCR_dist_ element. While (ex)T_reg_ cells increased IRF4 expression and accessibility to *IRF4* OCR_dist_, deletion of *IRF4* OCR_dist_ preserved T_reg_ cell lineage identity, suggesting that T_reg_ cell fate may be regulated by the amount of IRF4 under the control of *IRF4* OCR_dist_. Our data suggest IRF4 itself might act on OCR_dist_, forming a feedforward loop to sustain high expression of IRF4, thereby irreversibly driving T_reg_ cells to an alternative (ex)T_reg_ cell fate.

Several nonmutually exclusive mechanisms may explain how a quantitative change in IRF4 can alter T_reg_ cell fate. IRF4 and its partners are not known to target the *FOXP3* locus^31^; thus, its effect on FOXP3 silencing and T_reg_ cell instability is probably indirect. IRF4 is essential for TCR-induced proliferation and survival of CD8^+^^32,33^ and CD4^+^ effector T cells^34,35^, whereas limiting IRF4 activity or deletion of IRF4 favors induced T_reg_ cell differentiation^36^. Thus, an OCR_dist_-dependent increase in IRF4 expression in (ex)T_reg_ cells may support their survival and/or proliferation, and the overall enhancement in T_reg_ cell stability mediated by OCR_dist_ deletion may be due to decreased persistence of (ex)T_reg_ cells. This interpretation is supported by the increased proliferation and accumulation of (ex)T_reg_ cells in cultures exposed to inflammatory cytokines. Alternatively, increased IRF4 expression may directly contribute to chromatin remodeling in T_reg_ cells. IRF4 is a well-established mediator of effector T_reg_ programs, which is cooperatively controlled with FOXP3 both transcriptionally and via protein–protein interaction^26,37^. Increased IRF4 abundance may supersede FOXP3 partnership, enabling alternative transcriptional programs by IRF4 recruitment to low-affinity binding sites^34^ or heterodimerization with other partners^25^. These aberrant IRF4 activities may drive epigenetic rewiring that ultimately unravels T_reg_ cell lineage identity. In this regard, IRF4 is reported to interact with chromatin-modifying complexes, NuRD, SWI/SNF and SATB1^31^ that can lead to genome-wide changes in gene expression and chromatin accessibility.

Prior deletional studies have demonstrated a role of IRF4 in sustaining FOXP3 expression in human T_reg_ cells^16^ and in effector T_reg_ differentiation in mice and humans^37,38^. Findings from this study further suggest that excessive IRF4 expression promotes T_reg_ cell destabilization. Therefore, IRF4 abundance and activities in T_reg_ cells need to be tightly controlled to support effector T_reg_ cell function without disrupting T_reg_ cell identity. In this regard, Bach2 and Blimp1 repress Irf4 activity after TCR activation in T_reg_ cells^36,39^. Our results further suggest that the amount of IRF4 expression in T_reg_ cells is epigenetically controlled by restricting chromatin accessibility at *IRF4* OCR_dist_. This intricate module is vulnerable to the influence of inflammatory cytokines, particularly IL-23. The pronounced enrichment of AP-1 binding sites in inflammatory cytokine-exposed T_reg_ cells implicated a role of this family of TFs in mediating the effect of proinflammatory cytokines to reshape the T_reg_ cell epigenome. The crucial importance of *IRF4* OCR_dist_ in governing T_reg_ cell lineage identity may be due to its control of IRF4 abundance and partnership with AP-1 and ETS TFs. Collectively, our data are consistent with a model in which cytokine-induced AP-1 upregulation initiates genome-wide chromatin alterations, including at *IRF4* OCR_dist_, leading to increased IRF4 expression. IRF4, in turn, partners with AP-1 to solidify chromatin changes and drive T_reg_ cell lineage decommitment. This model requires further experimental validation.

Despite long-standing evidence that a T_reg_ cell-specific epigenetic landscape is required for preserving T_reg_ cell identity and function^10^, the regulatory networks that maintain the T_reg_ cell epigenome remain poorly defined. In this study, we identified *IRF4* noncoding regions that promote opposing T_reg_ cell fates, underscoring the untapped potential in targeting the epigenetic interactions that underpin cell identity. Beyond uncovering OCR_prox_ and OCR_dist_ as a novel regulatory module, we spotlight the genetic disruption of OCR_dist_ as a strategy that can be incorporated into the design of engineered T_reg_ cell therapies to ensure greater safety and suppressive potency. The in vitro model of human T_reg_ cell destabilization introduced in this study also lays the groundwork for future studies to identify additional regulatory nodes and deepen our understanding of the gene regulatory networks in coordinating the preservation or dismantlement of T_reg_ cell identity.

## Methods

### Xenogeneic GvHD mouse model

NOD.Cg-Prkdc^scid^ Il2rg^tm1Wjl^/SzJ (NSG) mice were obtained from Jackson Laboratories and bred at a specific pathogen-free facility at UCSF. For GvHD induction, male and female mice were randomized into five experimental groups, irradiated (1.75 Gy) 24 h before retro-orbital intravenous (i.v.) injection of 4 × 10^6^ PBMCs with 4 × 10^6^ in vitro-expanded, PBMC-matched T_reg_ cells. Mice were monitored and weighed twice weekly by researchers who were blinded to the treatment conditions. All experiments used a mixture of male and female mice ranging from 12 to 25 weeks of age at injection, and mice were randomly assigned across conditions by a separate blinded researcher. Mice were housed in a 12 h dark–12 h light cycle maintained at 20 °C and 55% humidity. Mice were killed on reaching a predefined humane end point of exhibiting >15% weight loss or worsening body conditions. All mouse experiments were performed according to a UCSF Institutional Animal Care and Use Program (IACUP)-approved protocol (IACUC protocol number AN200668). Two mice were excluded from the analysis owing to premature death from irradiation toxicity, thus not contributing to the intended experimental end point for GvHD.

### Human T cell isolation

PBMCs from de-identified, healthy human donors were commercially obtained through Stemcell Technologies. CD4^+^ T cells were negatively selected via the EasySep Human CD4^+^ T Cell Enrichment Cocktail (Stemcell Technologies). CD4^+^CD25^+^CD127^lo/−^ T_reg_ cells and CD4^+^CD25^−^CD127^+^ T_conv_ cells were subsequently isolated via FACS using FACSAria II or FACSAria III (Becton Dickinson) cell sorters to >98% purity. Isolated cells were washed with culture media and plated at 1 × 10^6^ cells ml^−1^. Details on antibodies used are presented in Supplementary Table 1.

### Human T cell culture

Freshly isolated primary human T_reg_ and T_conv_ cells were stimulated every 9 days with either CD3/CD28 Dynabeads (Thermo Fisher Scientific) at a 1:1 cell:bead ratio or with CD3/CD28/CD2 Immunocult (Stemcell Technologies) at 25 μl ml^−1^. Cultures were maintained in Roswell Park Memorial Institute medium (RPMI)-1640 (Gibco) supplemented with 10% heat-inactivated fetal bovine serum (FBS), 1% penicillin–streptavidin (Gibco), 1% MEM nonessential amino acids (Gibco), 10 mM HEPES (Gibco) and 1 mM sodium pyruvate (Gibco). Recombinant human IL-2 (Roche) was supplied at 300 IU ml^−1^ IL-2 during the initial 9-day stimulation cycle and 100 IU ml^−1^ IL-2 thereafter. For in vitro T_reg_ destabilization, recombinant human cytokines were used at the following concentrations unless otherwise indicated: 25 ng ml^−1^ IL-6 (Peprotech), 20 ng ml^−1^ IL-1β (Peprotech), 20 ng ml^−1^ IL-23 (Peprotech), 20 ng ml^−1^ IL-12 (Peprotech) and/or 10 ng ml^−1^ IL-4 (Peprotech). Every 2–3 days, cells were adjusted to 1 × 10^6^ cells ml^−1^ in culture media supplemented with the indicated cytokines.

### Genome editing

CRISPR–Cas9 ribonucleoprotein (RNP) complexes were prepared by co-incubating chemically modified CRISPR sgRNAs (Synthego) and purified recombinant Cas9-NLS protein (UC Berkeley QB3 Macrolab) at a 1:1 molar ratio. 1 × 10^6^ T_reg_ or T_conv_ cells were electroporated with 20 μM precomplexed RNPs 64 h after initial CD3/CD28 stimulation using program EO-115 on a Nucleofector 4D (Lonza). For excision edits with paired sgRNAs, 20 μM of each precomplexed RNP was co-delivered simultaneously. After electroporation, cells were plated in media supplemented with the indicated cytokines and maintained as described above. At 4 days following CRISPR–Cas9 RNP delivery, genomic DNA was collected from the edited cells for PCR amplification of the target loci and subsequent assessment of editing efficiency. Guide RNA sequences used for the following edits are listed below:

- AAVS1: GGGCCACUAGGGACAGGAU
- *IRF4* OCR_prox_: UAUAGUAGGUAAGAUCUCCA and GCUCCUCAGCCAGACCGCAG
- *IRF4* OCR_dist_: CCACUGGGAAACAAACCUGC and UACGUUGGAUCCCAUCACCC
- *IRF4* OCR_dist_ fragment 1: CAGAGGUGGCAGCUCUGCGU and UGCAUGTCCUUCCCGCAGGG
- *IRF4* OCR_dist_ fragment 2: UGCAUGTCCUUCCCGCAGGG and GAUGUGAGUGUAACCUCUGC

### CRISPRa overexpression

Vector pZR112 which contains CRISPRa machinery and mCherry reporter under an SFFV promoter was obtained from the Marson Lab^40^. sgRNA vectors containing a scaffold with MS2 stem-loops under the U6 promoter and the p65-HSF1 domains with GFP reporter under the hPGK promoter was constructed. Each sgRNA was cloned in the BsmBI (Golden Gate) cloning sites and short overlapping oligos containing the sgRNA sequence. sgRNA sequences were designed using CRISPick^41^ targeting −300 to +0 bases from the transcription start site and are as follows:

- BATF: AGAGAGAAAAATACAUAUCU
- IRF4: GUCCAACCCCCGGCCCCCAC
- Nontargeting control: CUGAAAAAGGAAGGAGUUGA

### Lentivirus production and transduction

Lentivirus was produced in Lenti-X HEK293T cell (Takara cat. no. 632180) according to a previously published protocol^40^. In brief, 293T cells were transfected with lentivial vector plasmid, psPAX2 and pMD2.G packaging and envelope plasmids using Lipofectamine 3000 (Invitrogen). Lentivirus was concentrated 100× using Lenti-X Concentrator (Takara).

Primary human T_reg_ cells were transduced 36 h after stimulation (Immunocult, 25 μl ml^−1^) with 3% v/v of the CRISPRa-mCherry vector and 60 h after stimulation with 1.5% v/v of the sgRNA-GFP vector. On day 7, GFP and mCherry double-positive cells were isolated via FACS, which was approximately 50% of the population, and reseeded at 1 × 10^6^ cells ml^−1^. Cells were then restimulated on day 10 and split into IL-2 only or IL-2 + inflammatory cytokine cocktail conditions. Subsequently, cells were restimulated as described previously. Successful overexpression of BATF and IRF4 was evaluated by intracellular flow cytometry.

### Flow cytometry

Cells were collected at the indicated time points and stained for flow cytometry. For intracellular staining, cells were fixed and permeabilized with the Foxp3 Transcription Factor Staining Buffer Set (eBioscience). For intracellular cytokine staining, cells were treated with 0.5 μg ml^−1^ PMA (Abcam), 1 μg ml^−1^ Ionomycin (Sigma) and Brefeldin A (ThermoFisher) for 5 h before staining. Data were collected on Fortessa flow cytometers (Becton Dickinson) or Aurora spectral flow cytometers (Cytek) and analyzed via FlowJo software (TreeStar). Details on antibodies used are presented in Supplementary Table 1.

### TSDR methylation analysis

HELIOS^+^, FOXP3^+^HELIOS^−^ and FOXP3^−^HELIOS^−^ cells were isolated from in vitro destabilization cultures via FACS, washed, pelleted and frozen at −80 °C. Cell pellets were submitted to EpigenDX according to vendor instructions. Genomic DNA isolation, bisulfite conversion and targeted pyrosequencing of the human FOXP3 TSDR (EpigenDX assay #ADS783-FS2) were performed at EpigenDx.

### In vitro suppression assay

Cryopreserved PBMCs were thawed and labeled with CellTraceViolet (ThermoFisher) according to manufacturer instructions. For in vitro suppression assays, 7.5 × 10^4^ CTV-labeled responder PBMCs were co-cultured with expanded T_reg_ cells at the indicated PBMC:T_reg_ cell ratios and stimulated with 1 μg ml^−1^ soluble anti-CD3 (clone Hit3a, BD Biosciences) and 0.5 μg ml^−1^ soluble anti-CD28 (clone 28.2, Biolegend) for 3 days. Cultures were then collected and stained with Fixable Live/Dead Viability Dye eFluor780 (ThermoFisher) and anti-CD4 PE, anti-CD8A fluorescein isothiocyanate (FITC) and anti-CD25 APC before flow cytometry and assessment of responder PBMC proliferation. Details on antibodies used are presented in Supplementary Table 1.

### Single-cell multiome (ATAC + gene expression) sequencing

Nuclei were isolated from in vitro-expanded T_reg_ and T_conv_ cells from three donors at the indicated time points before preparation of single-cell multiome sequencing libraries with the Chromium Next GEM Single Cell Multiome ATAC + Gene Expression kit (10x Genomics), according to manufacturer instructions. IL-2-only T_reg_ and T_conv_ cells from the three donors were each pooled to generate control samples. Sequencing was performed on a NovaSeq 6000 (Illumina) and data were processed with Bcl2fastq and Cell Ranger ARC 2.0.2 (10x Genomics). Data were subsequently analyzed using Seurat v4 and Signac v1.10 (refs. ^42,43^).

### Single-cell data preprocessing

The SoupX package^44^ was used to estimate and remove ambient RNA contamination in each sample. Low-quality cells were filtered by removing outliers in the transcriptomic data for the total transcript counts, number of distinct features and fraction of transcripts mapping to mitochondrial and ribosomal genes. Multiplets were removed using DoubletFinder. Similarly, outliers in the ATAC data were removed by examining the total fragment counts, number of distinct features and ratio of mononucleosomal (147–294 bp) to nucleosome-free (<147 bp) fragments. In addition, outlier cells were removed after scoring for the ratio of fragments centered at transcriptional start sites and the ratio of fragments in blacklisted regions, as defined by the ENCODE project, with additional filtering based on the total count:feature ratio for each modality. After quality filtering and doublet removal, 20,226 genes across 59,421 cells were retained from eight samples among two time points (day 16 and day 25). The genomic coordinates of overlapping peaks from each individual sample were merged to define a common ATAC peak set for peak quantification across samples.

Transcriptomic data were normalized via SCTransform in Seurat, with regression of cell-cycle genes (Seurat’s cc.genes) and mitochondrial genes. ATAC data were normalized via term-frequency inverse-document-frequency (TF-IDF), and dimensional reduction was performed on the TF-IDF matrix via singular value decomposition, as part of the standard Signac workflow. Batch correction was performed on both the RNA and ATAC modalities via the Harmony package grouped by sample^45^. A reference set for cell type and cell state annotation was constructed from a mixture of day 25 T_reg_ cell control, T_conv_ cell control and cytokine-treated T_reg_ cells, downsampled to equalize cell type and state representation. A multimodal UMAP embedding was generated through the WNN method with the FindMultiModalNeighbors function in Seurat. Clusters were identified on the WNN UMAP using the FindClusters function with the Smart Local Moving (SLM) algorithm (3) at resolution 0.8. Clusters containing cells with high ribosomal reads, high mitochondrial reads and/or disproportionate ATAC reads were removed, and the UMAP was recomputed at resolution 0.5. Individual samples were mapped to the reference and then merged to create the final UMAP.

### Single-cell OCR profiles and motif analysis

The model-based analysis of ChIP-seq (MACS2) package^46^ was used to identify peaks within OCRs. For comparisons of peak profiles, peaks present in at least 10% of the cells within the cluster were considered open in that cluster. Assessment of differential open chromatin accessibility was done with the FindMarkers function in Seurat using the Wilcoxon rank-sum test to identify peaks present in at least 10% of cells with a log_2_ fold-change threshold of 0.5. Bonferroni-adjusted *P* values were used to determine significance at a false discovery rate (FDR) <0.05. To identify differentially active TF motifs between exT_reg_ and stable T_reg_ cells (Fig. 3b), TF activity was inferred via the chromVAR package^47^ using the JASPAR 2020 database^48^. Differential TF motif activity was calculated with the FindMarkers function at an FDR <0.05 comparing cells in these two groups of clusters. Peak-expression linkage analyses were performed using the LinkPeaks function in Signac using default parameters based on the scMultiome dataset.

### Bulk ATAC-seq data processing

Paired-end bulk ATAC-seq reads were trimmed for adapter contamination and low-quality bases using Cutadapt (v3.5), retaining reads with a minimum length of 20 bp and a quality score cutoff of 20. Trimmed reads were aligned to the GRCh38/hg38 human reference genome using Bowtie2 (v2.4.5) with paired-end ATAC-seq parameters (--very-sensitive -X 2000 --no-mixed --no-discordant). Alignments were sorted and indexed with SAMtools (v1.13). Reads mapping to the mitochondrial genome were excluded, and only properly paired, primary alignments with mapping quality ≥30 were retained (samtools view -q 30 -f 2 -F 2828, excluding unmapped, mate-unmapped, secondary, supplementary and quality control (QC)-fail reads). PCR duplicate reads were then identified and removed using Picard MarkDuplicates (v2.27.4) for downstream analysis. One sample was excluded at this stage for failing to pass QC metrics.

### Bulk ATAC-seq peak calling and quantification

Chromatin accessibility peaks were called on each of the 22 bulk ATAC-seq samples (three to four donors × six conditions) using MACS2 (v2.2.9.1) with parameters: -f BAMPE -g hs --nomodel --keep-dup all -q 0.05. A fixed-width consensus peak set was generated from the MACS2 summit calls. Summits from all 22 samples were pooled and extended by 250 bp, producing 501-bp summit-centered candidate intervals. Intervals on noncanonical chromosomes were removed, intervals overlapping the ENCODE hg38 blacklist (ENCFF356LFX) were excluded and the remaining overlapping intervals were resolved by greedy score-based selection (iterative overlap peak merging^49^) using the corresponding MACS2 summit −log_10_(*q*-value) column. This yielded 104,749 nonoverlapping 501-bp consensus peaks. Per-sample quality control, including library complexity, fragment-size distribution and TSS enrichment, was performed with ataqv (v1.3.1) using GENCODE v45 basic-annotation TSSs. Fraction of reads in peaks (FRiP) was computed at the fragment level for each sample as the proportion of canonical-chromosome paired-end fragments overlapping the fixed-width consensus peak set by ≥ 1 bp. The corresponding FRiP values and QC metrics can be found in the analysis code^50^. Per-sample read counts over the fixed-width consensus peak set were quantified from de-duplicated BAM files using featureCounts (Subread v2.0.3) with parameters -p --countReadPairs and SAF-formatted peak annotations. For visualization, PCA was performed on log_2_-transformed counts per million (CPM) values over the 104,749 fixed-width consensus peaks. For downstream motif accessibility analysis, low-abundance peaks were excluded by requiring CPM ≥1 in at least three samples, yielding 92,157 retained peaks.

### TF motif scanning

Individual TF binding sites within the *IRF4* OCR_dist_ (chr6: 431,864–432,583; hg38/GRCh38) were identified using FIMO v5.5.9 (MEME Suite) with default parameters (*P* value threshold <1 × 10^−4^) and the vertebrate JASPAR 2026 core nonredundant motif database. FIMO hits were then retained at a *q*-value (FDR) ≤0.05. The resulting 90 motif occurrences were classified into TF families on the basis of JASPAR structural annotations. Within each TF family, hits were clustered around the highest-scoring same-family hit: any same-family hit whose motif midpoint lay within 7 bp of that anchor was merged with it, yielding 33 family-level binding sites. FIMO log-likelihood scores were mapped to lollipop stem length as a relative measure of predicted binding-site strength, and −log(*P* value) was mapped to circle area. The lollipop plot in Fig. 6a shows the 32 merged sites with lead FIMO score ≥8.0. Sites matching published position weight matrices for composite AICE, EICE and ISRE binding sites^51–53^ within the *IRF4* OCR_dist_ shown in Fig. 7a were identified using HOMER (v4.11) at a log-rank matching (HOMER score) threshold of 8.0.

### TF family motif chromatin accessibility analysis

Per-sample chromatin accessibility deviation *z*-scores were computed with pychromVAR (v0.0.4), a Python implementation of chromVAR^47^ from the filtered, fixed-width consensus ATAC-seq peak count matrix across 22 samples (three to four donors × six conditions: IL-2 only, +IL-6, +IL-1β, + IL-23, +all 3 cytokines and T_conv_). GC bias was computed using the hg38 reference genome, and background peak sets matched for GC content and average accessibility were generated using 2,000 background iterations. Motif scanning was performed against the JASPAR2026 CORE vertebrate nonredundant TF motif collection obtained through pyJASPAR (v4.0.0). In total 1,019 motifs were scanned, and motif matches were called using a motif matching threshold of *P* < 5 × 10^−5^.

JASPAR motifs were grouped into TF families according to TFClass annotations and curated by excluding multifamily heterodimer motifs and retaining motifs or motif families with a known T cell function, an association with T cell function through related family members and detectable T cell mRNA expression in Human Protein Atlas data. The AP-1 family included JUN, FOS, B-ATF and MAF families and was further partitioned into the AP-1 TRE binding and CRE-binding subsets. The TRE motif follows the consensus TGA(C/G)TCA and the CRE motif follows the consensus TGACGTCA. Each JASPAR matrix was assigned deterministically to either TRE or CRE on the basis of its consensus. This process annotated 751 of 1,019 JASPAR motifs into 53 TF families for downstream analysis.

Family-level chromVAR annotations were generated by collapsing motif matches within each family: a peak was assigned to a family if it contained at least one motif assigned to that family. chromVAR deviations were then recomputed on this peak-by-family annotation matrix, yielding one family-level chromVAR *z*-score per sample for each curated motif family.

Differential family-level accessibility was assessed with limma v3.62.2 (ref. ^54^). A linear model was fit with the design ~0 + treatment + donor, with treatment modeled as a no-intercept factor and donor as a blocking covariate to account for inter-individual variation. Treatment effects were tested as five prespecified contrasts, each comparing one condition with IL-2-only treatment (combo, IL-23, IL-1β, IL-6 and T_conv_ versus IL-2). Variances were moderated with empirical Bayes shrinkage without a mean–variance trend (limma eBayes with trend = FALSE), as appropriate for chromVAR *z*-scores. *P* values were adjusted for multiple testing (Benjamini–Hochberg) within each contrast across the 53 family-level tests. Figure 6b shows a curated subset of 26 of these 53 TF families; results for all 53 families are provided as source data.

In parallel, individual motif-level chromVAR *z*-scores were analyzed for all 1,019 JASPAR motifs using the same donor-blocked limma model and the same five IL-2-reference contrasts. Motif-level *P* values were Benjamini–Hochberg adjusted within each contrast across all 1,019 motif tests. Extended Data Fig. 8 displays limma delta *z*-scores for all individual motifs, grouped by curated TF-family annotation.

### Chromatin interaction analysis

Three-dimensional chromatin interactions at the IRF4 locus were assessed using promoter-capture Hi-C data from primary human CD4^+^ T cells^28^. Precomputed interaction scores were used to evaluate chromatin contacts between OCR_dist_-containing restriction fragments and promoter-baited fragments across the IRF4 locus. Interaction significance was determined by CHiCAGO scores^55^, with scores ≥5 considered significant. Contacts were examined in naive CD4^+^ T cells (nCD4) and activated CD4^+^ T cells (aCD4) to assess activation-dependent chromatin looping at OCR_dist_. OCR_dist_-anchored contacts were classified by whether they contacted the IRF4 promoter, EXOC2, DUSP22 or other neighboring loci.

### Statistics

Statistical analyses of experiment results were performed using GraphPad Prism 11.0.2. Specific tests used and sample sizes are described in the figure legends for Figs. 1, 3–8 and Extended Data Figs. 1–3, 7. In most cases, one-way analysis of variance (ANOVA) with multiple comparison tests was used for dataset containing three or more groups with one variable. Two-way ANOVA with a multiple comparison test was used for datasets with two independent variables. In cases of matched donors among various groups, repeated-measure one-way ANOVA or repeated-measure two-way ANOVA was used. In these repeated-measure comparison, mixed-effects analyses were used when some values were missing. Data distribution was assumed to be normal, but this was not formally tested owing to the inaccuracy of the test for small sample sizes (<100). For computational analyses, differential chromatin accessibility in single-cell and bulk ATAC-seq data was assessed using the Wald test (DESeq2) with Benjamini–Hochberg correction. Differential accessibility and TF motif activity in single-cell data were assessed using the Wilcoxon rank-sum test (Seurat FindMarkers) with Bonferroni correction. The correlation between OCR_dist_ and OCR_prox_ accessibility was assessed using the Pearson correlation coefficient. Promoter-capture Hi-C interaction significance was determined by CHiCAGO scores (threshold ≥5). All statistical tests were two-sided. No sample size calculations were performed to inform the study design. Group sizes were determined on the basis of prior experiences and generally exceeded the numbers used previously^16,20^.

### Reporting summary

Further information on research design is available in the Nature Portfolio Reporting Summary linked to this article.

## Online content

Any methods, additional references, Nature Portfolio reporting summaries, source data, extended data, supplementary information, acknowledgements, peer review information; details of author contributions and competing interests; and statements of data and code availability are available at https://doi.org/10.1038/s41590-026-02655-8.

## Supplementary information

Supplementary Information Supplementary Table 1.

Reporting Summary

Peer Review File

## Source data

Source Data Fig. 1 Statistical source data.

Source Data Fig. 2 Statistical source data.

Source Data Fig. 3 Statistical source data.

Source Data Fig. 4 Statistical source data.

Source Data Fig. 5 Statistical source data.

Source Data Fig. 6 Statistical source data.

Source Data Fig. 7 Statistical source data.

Source Data Fig. 8 Statistical source data.

Source Data Extended Data Fig. 1 Statistical source data.

Source Data Extended Data Fig. 2 Statistical source data.

Source Data Extended Data Fig. 3 Statistical source data.

Source Data Extended Data Fig. 5 Statistical source data.

Source Data Extended Data Fig. 6 Statistical source data.

Source Data Extended Data Fig. 7 Statistical source data.

Source Data Extended Data Fig. 8 Statistical source data.

## Data Availability

The scMulti-omic data described in this paper have been deposited in the National Center for Biotechnology Information (NCBI) Gene Expression Omnibus (GEO) database (accession number GSE281099). The bulk ATAC-seq data described in this paper have been deposited in the National Center for Biotechnology Information (NCBI) Gene Expression Omnibus (GEO) database (accession number GSE331192). Raw flow data can be obtained on request to the corresponding authors. Source data are provided with this paper.

## Extended data

**Extended Data Table 1:** Concentrations of IL-6, IL-1β, and IL-23 reported in patients with chronic inflammation

	IL-6 (ng/ml)	IL-1*β* (ng/ml)	IL-23 (ng/ml)
Synovium, RA	Up to 20^56^ Up to 35^57^	Up to 8^57^	5.19±1.8^58^
Synovium, JIA	2.6 – 3.0 ± 0.5 - 1.3^59^	0.01-0.03 ± 0.5 - 1.3^59^	
CSF, Neuroinflammation	up to 1^60^	up to 0.15^60^	
CSF, Bacterial meningitis	up to 6^61^		
Plasma, Septic shock	Up to 2,400; Mean ~200^62^		
Ascites, Cancer	Up to >100; Mean: ~50^19^ 0.4-80.5^63^		
Serum, kidney transplant			Up to 1.7^9^

## Code availability

The single-cell multi-omic (scATAC-seq and scRNA-seq) and bulk ATAC-seq data analyses were performed using open-source algorithms as described in Methods. Codes used for analysis are available via Zenodo at https://doi.org/10.5281/zenodo.21765753 (ref. ^50^).
