# Supplementary material for: Deletion of a distal IRF4 element prevents inflammation-induced reprogramming of human regulatory T cell fate
Source: Nat Immunol. 2026 Sep 21;27(10):2143–55. doi: 10.1038/s41590-026-02655-8 (PMC13630568; doi:10.1038/s41590-026-02655-8)
Supplement: Supplementary file 1 [file 41590_2026_2655_MOESM1_ESM.pdf]

nature immunology

Supplementary information

https://doi.org/10.1038/s41590-026-02655-8

Deletion of a distal *IRF4* element prevents inflammation-induced reprogramming of human regulatory T cell fate

In the format provided by the authors and unedited

Table S1. List of Antibodies for Flow Cytometry

Antibodies for Treg cell isolation (FACS)				
staining reagent	conjugate	clone	vendor	cat no.
anti-CD4	FITC	RPA-T4	Biolegend	300538
anti-CD25	APC	4E3	ThermoFisher	17-0257-42
anti-CD127	PE	hIL-7R-M21	BD Biosciences	557938
Antibodies and reagents for analytical flow cytometry				
staining reagent	conjugate	clone	vendor	
Fixable Live/Dead Viability Dye	eFluor780		eBioscience	65-0865
Fixable Viability Dye	Zombie UV		Biolegend	423108
anti-CD4	PE	RPA-T4	BD Biosciences	555347
anti-CD8A	FITC	SK1	BD Biosciences	570817
Anti-CD25	APC	2A3	BD Biosciences	340938
anti-CD25	AF700	BC96	Biolegend	302622
anti-CD25	BV711	2A3	BD Biosciences	563159
anti-ICOS	PE-Cy7	C398.4a	Biolegend	313520
anti-ICOS	BUV805	DX29	BD Biosciences	748903
anti-TIGIT	PerCP-eFluor710	MBSA43	ThermoFisher	46-9500-42
anti-TIGIT	BV605	15153G	Biolegend	372712
anti-CD226	BV785	11A8	Biolegend	338322
anti-CD27	BV510	M-271	Biolegend	302836
anti-FOXP3	AF647	206D	Biolegend	320114
anti-HELIOS	FITC	22F6	Biolegend	137214
anti-HELIOS	PE-Cy7	22F6	Biolegend	137236
anti-CTLA4	PE	14D3	ThermoFisher	12-1529-42
anti-CTLA4	BUV737	14D3	ThermoFisher	367-1529-42
anti-CD40LG	BV421	24-31	Biolegend	310824
anti-IRF4	AF700	3E4	ThermoFisher	56-9858-82
anti-KI-67	BUV563	SolA15	ThermoFisher	365-5698-82
anti-IL-17A	PE	BL168	Biolegend	512306
anti-IFNg	BV785	4S.B3	Biolegend	502542
anti-IL-4	BV421	MP4-25D2	Biolegend	500826
anti-TNFa	FITC	Mab11	Biolegend	502906
anti-BATF	PerCP-eFluor710	MBM7C7	ThermoFisher	46-9860-42
