# Supplementary material for: Deletion of a distal IRF4 element prevents inflammation-induced reprogramming of human regulatory T cell fate
Source: Nat Immunol. 2026 Sep 21;27(10):2143–55. doi: 10.1038/s41590-026-02655-8 (PMC13630568; doi:10.1038/s41590-026-02655-8)
Supplement: Supplementary file 2 [file 41590_2026_2655_MOESM2_ESM.pdf]

nature portfolio

Corresponding author(s): Qizhi Tang, Patrick Ho

Last updated by author(s): Aug 10, 2026

Reporting Summary

Nature Portfolio wishes to improve the reproducibility of the work that we publish. This form provides structure for consistency and transparency in reporting. For further information on Nature Portfolio policies, see our Editorial Policies and the Editorial Policy Checklist.

Statistics

For all statistical analyses, confirm that the following items are present in the figure legend, table legend, main text, or Methods section.

n/a	Confirmed	
☐	☒	The exact sample size (*n*) for each experimental group/condition, given as a discrete number and unit of measurement
☐	☒	A statement on whether measurements were taken from distinct samples or whether the same sample was measured repeatedly
☐	☒	The statistical test(s) used AND whether they are one- or two-sided<br>*Only common tests should be described solely by name; describe more complex techniques in the Methods section.*
☐	☒	A description of all covariates tested
☐	☒	A description of any assumptions or corrections, such as tests of normality and adjustment for multiple comparisons
☐	☒	A full description of the statistical parameters including central tendency (e.g. means) or other basic estimates (e.g. regression coefficient) AND variation (e.g. standard deviation) or associated estimates of uncertainty (e.g. confidence intervals)
☒	☐	For null hypothesis testing, the test statistic (e.g. *F*, *t*, *r*) with confidence intervals, effect sizes, degrees of freedom and *P* value noted<br>*Give P values as exact values whenever suitable.*
☒	☐	For Bayesian analysis, information on the choice of priors and Markov chain Monte Carlo settings
☒	☐	For hierarchical and complex designs, identification of the appropriate level for tests and full reporting of outcomes
☒	☐	Estimates of effect sizes (e.g. Cohen's *d*, Pearson's *r*), indicating how they were calculated

Our web collection on statistics for biologists contains articles on many of the points above.

Software and code

Policy information about availability of computer code

Data collection	FACSDiva v9.0 (BD) | flow cytometry data acquisition<br>SpectroFlo v3.2.1 (Cytek) | spectral flow cytometry data acquisition
Data analysis	FlowJo v10.8.1 (BD) | flow cytometry data analysis<br>Excel v16.89.1 (Mac) | analysis of bulk data<br>Prism v11.0.2 (GraphPad) | graph generation and statistical analysis<br>CellRanger ARC v2.0.0 | demultiplexing and sequence alignment of 10X Genomics single-cell multiome (ATAC and GEX) sequencing data with hg38 reference genome<br>Seurat v4.3 | GEX data pre-processing, normalization, dimensional reduction, cell type identification, differention gene expression analyses, and data visualization<br>Signac v1.10 | ATAC data pre-processing, normalization, and dimensional reduction, and data visualization<br>SoupX v1.6.2 | ambient RNA removal during single-cell GEX data pre-processing<br>MACS2 v2.2.9.1 | peak calling for ATAC data analyses<br>DESeq2 v1.36.0 | bulk ATAC-seq analysis<br>HOMER v4.11 | transcription factor enrichment analysis<br>JASPAR 2026 vertebrate and FIMO | transcription factor motif identification<br>UCSC 100-way vertebrate alignment | phylogenetic conservation analysis<br>pychromVAR v0.0.4 | transcription factor motif accessibility analysis

For manuscripts utilizing custom algorithms or software that are central to the research but not yet described in published literature, software must be made available to editors and reviewers. We strongly encourage code deposition in a community repository (e.g. GitHub). See the Nature Portfolio guidelines for submitting code & software for further information.

Data

Policy information about availability of data

All manuscripts must include a data availability statement. This statement should provide the following information, where applicable:

- Accession codes, unique identifiers, or web links for publicly available datasets
- A description of any restrictions on data availability
- For clinical datasets or third party data, please ensure that the statement adheres to our policy

The scMulti-omic data described in the manuscript have been deposited in the National Center for Biotechnology Information (NCBI) Gene Expression Omnibus (GEO) database. The accession number is GSE281099. The dataset is public.

The bulk ATACseq data described in the manuscript have been deposited in the National Center for Biotechnology Information (NCBI) Gene Expression Omnibus (GEO) database. The accession number is GSE331192 The dataset is public..

All other data associated with this manuscript are included in the manuscript and extended information.

Research involving human participants, their data, or biological material

Policy information about studies with human participants or human data. See also policy information about sex, gender (identity/presentation), and sexual orientation and race, ethnicity and racism.

Reporting on sex and gender	All primary human T cells used in this study were isolated from de-identified leukapaks purchased from StemCell Technologies. Therefore, this is not a human subject study.
Reporting on race, ethnicity, or other socially relevant groupings	Donors were deidentified. No demographic data are available
Population characteristics	*Describe the covariate-relevant population characteristics of the human research participants (e.g. age, genotypic information, past and current diagnosis and treatment categories). If you filled out the behavioural & social sciences study design questions and have nothing to add here, write "See above."*
Recruitment	*Describe how participants were recruited. Outline any potential self-selection bias or other biases that may be present and how these are likely to impact results.*
Ethics oversight	*Identify the organization(s) that approved the study protocol.*

Note that full information on the approval of the study protocol must also be provided in the manuscript.

Field-specific reporting

Please select the one below that is the best fit for your research. If you are not sure, read the appropriate sections before making your selection.

☒ Life sciences ☐ Behavioural & social sciences ☐ Ecological, evolutionary & environmental sciences

For a reference copy of the document with all sections, see nature.com/documents/nr-reporting-summary-flat.pdf

Life sciences study design

All studies must disclose on these points even when the disclosure is negative.

Sample size	No sample size calculations were performed to inform the study design. Group sizes were determined based on prior experiences and generally exceeded the numbers used previously.
Data exclusions	In the in vivo experiment reported in Figure 8, 2 mice (one each from the AAVS1-CTRL and IRF4 OCRdist-INFL groups) were excluded due to premature death from irradiation toxicity thus did not contribute to the intended experimental endpoint for GvHD.<br><br>From the bulk ATACseq data set, one sample in the IL-6 condition was excluded due to not passing sequencing QC metrics.
Replication	Data were generated using T cells from 3 to 22 unrelated human T cell donors for each experiment, as indicated in figure legends. The xenogeneic GVHD experiment used PBMC from 2 unrelated donors each infused into 6 to 8 mice.
Randomization	For the in vivo xenogeneic GvHD study in Figure 8, mice were weighed on Day -1 before irradiation and randomized into equal bins according to sex and weight. Each bin was then randomly assigned to each experimental treatment group using RandoMice v1.1.7 software tool.
Blinding	For the in vivo xenogeneic GvHD study in Figure 8, researchers responsible for cell injection and data collection were blinded to the treatment groups.

Reporting for specific materials, systems and methods

We require information from authors about some types of materials, experimental systems and methods used in many studies. Here, indicate whether each material, system or method listed is relevant to your study. If you are not sure if a list item applies to your research, read the appropriate section before selecting a response.

Materials & experimental systems

n/a	Involved in the study
☐	☒ Antibodies
☐	☒ Eukaryotic cell lines
☒	☐ Palaeontology and archaeology
☐	☒ Animals and other organisms
☒	☐ Clinical data
☒	☐ Dual use research of concern
☒	☐ Plants

Methods

n/a	Involved in the study
☒	☐ ChIP-seq
☐	☒ Flow cytometry
☒	☐ MRI-based neuroimaging

Antibodies

Antibodies used	All antibody used are from reputable commercial vendors. Detailed information of these antibodies are summarized in Supplementary Table 1.
Validation	The specificity of the antibodies used were validated by vendors. All antibodies were titered to optimize detection while minimizing background prior to their use in experiments.

Eukaryotic cell lines

Policy information about cell lines and Sex and Gender in Research

Cell line source(s)	Lenti-X HEK293t cell (Takara cat. #632180)
Authentication	Validated by Vendor
Mycoplasma contamination	Vendor provided certification that the cells were mycoplasma free.
Commonly misidentified lines (See ICLAC register)	*Name any commonly misidentified cell lines used in the study and provide a rationale for their use.*

Animals and other research organisms

Policy information about studies involving animals; ARRIVE guidelines recommended for reporting animal research, and Sex and Gender in Research

Laboratory animals	NOD.CgPrkdcscid Il2rgtm1Wjl/SzJ (NSG) mice (male and female) were purchased from JAX Labs. They were bred at UCSF and used in the in vivo experiment in Figure 8.
Wild animals	No wild animals were used.
Reporting on sex	Both male and female mice were used and randomized to ensure equal distribution of the two sexes in all the experimental groups.
Field-collected samples	None.
Ethics oversight	All mouse experiments were performed according to a UCSF Institutional Animal Care and Use Program (IACUP)-approved protocol (IACUC protocol no. AN200668).

Note that full information on the approval of the study protocol must also be provided in the manuscript.

Plants

Seed stocks: None

Novel plant genotypes: *Describe the methods by which all novel plant genotypes were produced. This includes those generated by transgenic approaches, gene editing, chemical/radiation-based mutagenesis and hybridization. For transgenic lines, describe the transformation method, the number of independent lines analyzed and the generation upon which experiments were performed. For gene-edited lines, describe the editor used, the endogenous sequence targeted for editing, the targeting guide RNA sequence (if applicable) and how the editor was applied.*

Authentication: *Describe any authentication procedures for each seed stock used or novel genotype generated. Describe any experiments used to assess the effect of a mutation and, where applicable, how potential secondary effects (e.g. second site T-DNA insertions, mosiacism, off-target gene editing) were examined.*

Flow Cytometry

Plots

Confirm that:

☒ The axis labels state the marker and fluorochrome used (e.g. CD4-FITC).

☒ The axis scales are clearly visible. Include numbers along axes only for bottom left plot of group (a 'group' is an analysis of identical markers).

☒ All plots are contour plots with outliers or pseudocolor plots.

☒ A numerical value for number of cells or percentage (with statistics) is provided.

Methodology

Sample preparation: For live/dead staining, cells were washed with PBS, then resuspended in Fixable Viability Dye in PBS.

For surface staining, samples were then washed with FACS buffer (PBS + 2% FBS) and stained with flurophore-conjugated antibodies in FACS buffer for 30 min at 4°C. Samples were washed with FACS buffer prior to fixation with the FOXP3 FIX/Perm kit (eBiosciences) for 45 min at 4°C.

For intracellular staining, samples were washed with Perm buffer (FOXP3 FIX/Permeabilization kit) and stained fluorophore-conjugated antibodies in Perm buffer for 30 min at 4°C. Stained samples were washed with FACS and resuspended in FACS buffer for data acquisition.

Instrument: BD Fortessa X-20, Cytek Aurora (5-laser)

Software: FACSDiva v9.0 (BD Biosciences), SpectroFlo v3.2.1 (Cytek), FlowJo v.10.8.1 (BD Biosciences)

Cell population abundance: PBMCs were isolated from leukopaks and pre-enriched via magnetic negative selection for CD4 T cells (Stemcell Technologies cat. no. 17952). For some donors, samples were additionally enriched with positive selection for CD25+ cells (Stemcell Technologies cat. no. 18063). The CD4-enriched fraction was subsequently stained with anti-CD4_FITC, anti-CD25_APC, and anti-CD127_PE. The CD4+CD25+CD127lo/- Tregs and CD4+CD25-CD127+ Tconvs were collected. The fraction of CD4+CD25+CD127lo/- Tregs within the pre-enriched CD4 T-cell population ranged from 2.5-7.0%, depending on the human donor. Due to limiting Treg cell number recovered and large number of Tregs needed for experiments, all collected Tregs were cultured as described. The stimulated cells were stained for FOXP3 and HELIOS expression on 7 days post-FACS isolation. Expression of FOXP3 and HELIOS in sorted Tregs was assessed relative to donor-matched Tconvs to gauge purity of the original FACS isolation. The validity of using FOXP3+HELIOS+ markers to identify lineage-commited Tregs was confirmed by the demethylaotion status of the Treg-specific demethylated region (TSDR) in these cells shown in Figure 1d.

Gating strategy: Samples were gated on lymphocytes (SSC-A vs. FSC-A) and singlets (FSC-H vs. FSC-A), and dead cells were excluded based on positive Fixable Viability Dye staining. Cell populations of interest were analyzed according to expression of defining markers (e.g., FOXP3+ HELIOS+ for stable Tregs).

☒ Tick this box to confirm that a figure exemplifying the gating strategy is provided in the Supplementary Information.
