# Supplementary material for: Deletion of a distal IRF4 element prevents inflammation-induced reprogramming of human regulatory T cell fate
Source: Nat Immunol. 2026 Sep 21;27(10):2143–55. doi: 10.1038/s41590-026-02655-8 (PMC13630568; doi:10.1038/s41590-026-02655-8)
Supplement: Supplementary file 3 [file 41590_2026_2655_MOESM3_ESM.pdf]

nature portfolio

# Peer Review File

# Deletion of a Distal IRF4 Element Prevents Inflammation-induced Reprogramming of Human Regulatory T-cell Fate

Corresponding Author: Dr QIZHI TANG

**This file contains all editorial decision letters in order by version, followed by all author rebuttals in order by version.**

Version 0:

Reviewer comments:

Reviewer #1

(Remarks to the Author)
The study presents a potentially novel finding regarding the role of the distal IRF4 element (OCRdist) in Treg destabilization. This finding is intriguing given prior studies on IRF4's role in Treg stability and Treg effector activity [PMID 32989329, 32125291]. However, the work remains preliminary and has significant limitations, particularly regarding in vivo relevance and mechanistic insight.

In vivo validation, such as data from human autoimmune conditions, would enhance the manuscript's impact. Restricting the study to Treg adoptive cell therapy limits its broader applicability. The role of IL-6/IL-1β/IL-23 in Treg instability is already well-established, diminishing the novelty of these findings. Furthermore, it is unclear how the tested conditions are physiologically relevant (e.g., concentration).

The effect and mechanism of IRF4 OCRdist on Treg stability remains unclear and is constrained by significant gaps. What specific roles do the individual cytokines play, and how do they influence IRF4 OCRdist? What binds to this OCR? Is IRF4 OCRdist independent of previously identified Treg stabilization mechanisms such as TSDR, CNS2? Additionally, since IRF4 has been shown to promote FOXP3 expression, how it contributes to Treg destabilization in this context requires further clarification. Is it possible that this OCR controls other loci than IRF4 (DUSP22 is in the proximity and affect TCR signaling [PMID36765305,38225265))?
The reliance on in vitro models raises concerns about artifacts, and interpretation of the experiments is open to alternative interpretations. The 2^15-fold expansion amplifies even minor contaminants to potentially significant levels. The small effect size of IRF4 OCRdist knockout (figure 5), the confinement of observed effects to FOXP3-HELIOS- cells, combined with the finding that OCRdist is open in Tconv and closed in Tregs, raises the question of whether OCRdist amplifies or stabilizes a small population of Tconv/exTregs rather than destabilizing Tregs as a whole. Additional experiments would greatly strengthen the manuscript. For example, analyzing FOXP3-low cells, measuring absolute cell counts in vitro, and employing single-cell RNA sequencing combined with TCR sequencing as fate-mapping tools would address several open questions. Furthermore, a mouse model with IRF4 OCRdist deletion, combined with Treg fate mapping tools in autoimmune models, would provide valuable insights. It is also worth exploring whether IRF4 OCRdist is present in mice and functions similarly.

Finally, the manuscript appears incomplete. Methodological details are insufficient, statistical analyses—particularly for genomics—are minimal, and several key figure panels (1d, 5c, and 6a) are missing.

Reviewer #3

(Remarks to the Author)
In their study, Ho et al. develop an in vitro system to model the loss of Treg stability driven by inflammatory cytokine exposure that is a major barrier to the successful implementation of adoptive Treg cell therapy to promote tolerance in autoimmunity and transplantation. They demonstrate that repeated stimulation of purified human Treg in the presence of an inflammatory cytokine cocktail leads to loss of Foxp3 and Helios expression associated with remethylation of the Treg-specific Foxp3 TSDR. Destabilized Treg were functionally less suppressive, and instead acquired the ability to produce pro-inflammatory cytokines. Single cell multi-omic analyses identified transcriptional and epigenetic changes associated with de-stabilized Treg, including a shift in the relative accessibility of two open chromatin regions in the IRF4 locus from an

intronic proximal OCR (OCRprox) to a distal OCR (OCRdist) that is normally closed in Treg cells but open in conventional T cells. They further show that the OCRdist controls TCR-mediated upregulation of IRF4, and most importantly that CRISPR-mediated deletion of OCRdist promotes Treg stability during expansion in the presence of inflammatory cytokines, and enhances regulatory activity in a xenograft model of GVHD.

Overall, this is a well-performed study with many strengths. The identification of an open chromatin region that regulates Treg stability in the face of inflammatory cytokine treatment is highly significant and directly translatable in efforts to develop adoptive Treg therapy. The experiments are well-performed and the data are statistically robust and convincing. Data presentation and writing are clear and concise. Weaknesses are detailed as follows:

1. Although this manuscript is highly significant translationally, a weakness is the lack of mechanistic experiments to better understand how OCRdist controls Treg stability, or how this OCR is targeted by the inflammatory cytokines present during Treg activation/expansion.

2. (Minor) Functional experiments on OCRdist-deleted cells focus only on cell destabilized with the IL-6, IL-1, IL-23 combination. Do other inflammatory cytokines that induce Treg destabilization (albeit less efficiently) as outlined in Fig 1/Fig S1 also promote opening of OCRdist?

3. (Minor) The 'peak linkages' data in Fig 4C is not well-explained. Was this determined from the multi-omics data set? How? Is this based on prior chromatin-confirmation studies? More details are needed to properly contextualize those results.

Decision Letter:

2nd Jan 2025

Dear Tang,

Thank you for providing a point-by-point response to the referees' comments on your manuscript entitled, "Deletion of a Distal IRF4 Element Prevents Inflammation-induced Reprogramming of Human Regulatory T-cell Fate". Given that referee #2X has gone AWOL, we felt that further delays would be not not fair to you, the authors. Hence, we therefore invite you to revise your manuscript taking into account the reviewer comments that I have already provided to you earlier.

As noted previously, while referees #1 and #3 find your work of considerable potential interest, they have raised a number of concerns that must be addressed. In light of these comments, we cannot accept the current manuscript for publication, but would be very interested in considering a revised version that addresses these serious concerns along the lines proposed in your point-by-point rebuttal.

We invite you to submit a substantially revised manuscript, however please bear in mind that we will be reluctant to approach the referees again in the absence of major revisions.

Specifically, the revision should include new experiments to address:

(1) examine how individual inflammatory cytokines influence the chromatin accessibility status of IRF4 OCRdist regulatory region

(2) perform analysis to identify candidate TFs that influence chromatin accessibility of OCRdist or support cis-regulatory interactions

(3) perform bioinformatic analysis of publicly available Hi-C data to determine whether IRF4 OCRdist may have regulatory roles for other loci

(4) perform analysis to determine if a homologous regulatory region is shared by the mouse Irf4 locus and human IRF4 OCRdist

(5) perform analysis with IL-4 cytokine stimulation to evaluate whether OCRdist might be a more general feature of Treg instability in other inflammatory cytokine contexts

Please include the additional textual clarifications as indicated in your response letter.

When you revise your manuscript, please take into account all reviewer and editor comments, please highlight all changes in the manuscript text file in Microsoft Word format.

We are committed to providing a fair and constructive peer-review process. Do not hesitate to contact us if there are specific requests from the reviewers that you believe are technically impossible or unlikely to yield a meaningful outcome.

When revising your manuscript:

* Include a “Response to referees” document detailing, point-by-point, how you addressed each referee comment. If no action was taken to address a point, you must provide a compelling argument. This response will be sent back to the referees along with the revised manuscript.

* If you have not done so already please begin to revise your manuscript so that it conforms to our Article format instructions at http://www.nature.com/ni/authors/index.html. Refer also to any guidelines provided in this letter.

* Include a revised version of any required reporting checklist. It will be available to referees (and, potentially, statisticians) to aid in their evaluation if the manuscript goes back for peer review. A revised checklist is essential for re-review of the paper.

The Reporting Summary can be found here:
https://www.nature.com/documents/nr-reporting-summary.pdf

When submitting the revised version of your manuscript, please pay close attention to our href="https://www.nature.com/nature-portfolio/editorial-policies/image-integrity">Digital Image Integrity Guidelines.</a> and to the following points below:

-- that unprocessed scans are clearly labelled and match the gels and western blots presented in figures.
-- that control panels for gels and western blots are appropriately described as loading on sample processing controls
-- all images in the paper are checked for duplication of panels and for splicing of gel lanes.

Extended Data figures and tables are online-only (appearing in the online PDF and full-text HTML version of the paper), peer-reviewed display items that provide essential background to the Article but are not included in the printed version of the paper due to space constraints or being of interest only to a few specialists. A maximum of ten Extended Data display items (figures and tables) is typically permitted. When re-submitting your manuscript, please ensure that any supplementary figures and tables that are more critical to the manuscript’s conclusions are converted to Extended data to increase these data’s visibility.

Finally, please ensure that you retain unprocessed data and metadata files after publication, ideally archiving data in perpetuity, as these may be requested during the peer review and production process or after publication if any issues arise.

You may use the link below to submit your revised manuscript and related files:

Link Redacted

**Note:** This URL links to your confidential home page and associated information about manuscripts you may have submitted, or that you are reviewing for us. If you wish to forward this email to co-authors, please delete the link to your homepage.

If you wish to submit a suitably revised manuscript we would hope to receive it within 6 months. If you cannot send it within this time, please let us know. We will be happy to consider your revision so long as nothing similar has been accepted for publication at Nature Immunology or published elsewhere.

Nature Immunology is committed to improving transparency in authorship. As part of our efforts in this direction, we are now requesting that all authors identified as ‘corresponding author’ on published papers create and link their Open Researcher and Contributor Identifier (ORCID) with their account on the Manuscript Tracking System (MTS), prior to acceptance. ORCID helps the scientific community achieve unambiguous attribution of all scholarly contributions. You can create and link your ORCID from the home page of the MTS by clicking on ‘Modify my Springer Nature account’. For more information please visit please visit <a href="http://www.springernature.com/orcid">www.springernature.com/orcid</a>.

Please do not hesitate to contact me if you have any questions or would like to discuss the required revisions further.

Thank you for the opportunity to review your work.

Kind regards & best wishes for the New Year!

Laurie

Laurie A. Dempsey, Ph.D.
Senior Editor
Nature Immunology
l.dempsey@us.nature.com
ORCID: 0000-0002-3304-796X

Referee expertise:

Referee #1: Tissue Treg cells

Referee #3: Treg cell functionality

Reviewers' Comments:

Reviewer #1 (Remarks to the Author):

The study presents a potentially novel finding regarding the role of the distal IRF4 element (OCRdist) in Treg destabilization. This finding is intriguing given prior studies on IRF4's role in Treg stability and Treg effector activity [PMID 32989329, 32125291]. However, the work remains preliminary and has significant limitations, particularly regarding in vivo relevance and mechanistic insight.

In vivo validation, such as data from human autoimmune conditions, would enhance the manuscript's impact. Restricting the study to Treg adoptive cell therapy limits its broader applicability. The role of IL-6/IL-1β/IL-23 in Treg instability is already well-established, diminishing the novelty of these findings. Furthermore, it is unclear how the tested conditions are physiologically relevant (e.g., concentration).

The effect and mechanism of IRF4 OCRdist on Treg stability remains unclear and is constrained by significant gaps. What specific roles do the individual cytokines play, and how do they influence IRF4 OCRdist? What binds to this OCR? Is IRF4 OCRdist independent of previously identified Treg stabilization mechanisms such as TSDR, CNS2? Additionally, since IRF4 has been shown to promote FOXP3 expression, how it contributes to Treg destabilization in this context requires further clarification. Is it possible that this OCR controls other loci than IRF4 (DUSP22 is in the proximity and affect TCR signaling [PMID36765305,38225265))?
The reliance on in vitro models raises concerns about artifacts, and interpretation of the experiments is open to alternative interpretations. The 2^15-fold expansion amplifies even minor contaminants to potentially significant levels. The small effect size of IRF4 OCRdist knockout (figure 5), the confinement of observed effects to FOXP3-HELIOS- cells, combined with the finding that OCRdist is open in Tconv and closed in Tregs, raises the question of whether OCRdist amplifies or stabilizes a small population of Tconv/exTregs rather than destabilizing Tregs as a whole. Additional experiments would greatly strengthen the manuscript. For example, analyzing FOXP3-low cells, measuring absolute cell counts in vitro, and employing single-cell RNA sequencing combined with TCR sequencing as fate-mapping tools would address several open questions. Furthermore, a mouse model with IRF4 OCRdist deletion, combined with Treg fate mapping tools in autoimmune models, would provide valuable insights. It is also worth exploring whether IRF4 OCRdist is present in mice and functions similarly.

Finally, the manuscript appears incomplete. Methodological details are insufficient, statistical analyses—particularly for genomics—are minimal, and several key figure panels (1d, 5c, and 6a) are missing.

Reviewer #3 (Remarks to the Author):

In their study, Ho et al. develop an in vitro system to model the loss of Treg stability driven by inflammatory cytokine exposure that is a major barrier to the successful implementation of adoptive Treg cell therapy to promote tolerance in autoimmunity and transplantation. They demonstrate that repeated stimulation of purified human Treg in the presence of an inflammatory cytokine cocktail leads to loss of Foxp3 and Helios expression associated with remethylation of the Treg-specific Foxp3 TSDR. Destabilized Treg were functionally less suppressive, and instead acquired the ability to produce pro-inflammatory cytokines. Single cell multi-omic analyses identified transcriptional and epigenetic changes associated with de-stabilized Treg, including a shift in the relative accessibility of two open chromatin regions in the IRF4 locus from an intronic proximal OCR (OCRprox) to a distal OCR (OCRdist) that is normally closed in Treg cells but open in conventional T cells. They further show that the OCRdist controls TCR-mediated upregulation of IRF4, and most importantly that CRISPR-mediated deletion of OCRdist promotes Treg stability during expansion in the presence of inflammatory cytokines, and enhances regulatory activity in a xenograft model of GVHD.

Overall, this is a well-performed study with many strengths. The identification of an open chromatin region that regulates Treg stability in the face of inflammatory cytokine treatment is highly significant and directly translatable in efforts to develop adoptive Treg therapy. The experiments are well-performed and the data are statistically robust and convincing. Data presentation and writing are clear and concise. Weaknesses are detailed as follows:

1. Although this manuscript is highly significant translationally, a weakness is the lack of mechanistic experiments to better understand how OCRdist controls Treg stability, or how this OCR is targeted by the inflammatory cytokines present during Treg activation/expansion.

2. (Minor) Functional experiments on OCRdist-deleted cells focus only on cell destabilized with the IL-6, IL-1, IL-23 combination. Do other inflammatory cytokines that induce Treg destabilization (albeit less efficiently) as outlined in Fig 1/Fig S1 also promote opening of OCRdist?

3. (Minor) The 'peak linkages' data in Fig 4C is not well-explained. Was this determined from the multi-omics data set? How? Is this based on prior chromatin-confirmation studies? More details are needed to properly contextualize those results.

Version 1:

Reviewer comments:

Reviewer #1

(Remarks to the Author)
The manuscript is much improved and clearer, with more in-depth mechanistic insights and important new analyses and experiments, including gain-of-function studies. Well done. I recommend it for publication.

Reviewer #3

(Remarks to the Author)
The authors have substantially revised this manuscript in response to the initial critiques, and I have not further comments. This is an important and novel contribution to the field.

Decision Letter:

Our ref: NI-A38848A

6th Jul 2026

Dear Tang,

Thank you for submitting your revised manuscript "Deletion of a Distal IRF4 Element Prevents Inflammation-induced Reprogramming of Human Regulatory T-cell Fate" (NI-A38848A). It has now been seen by the original referees and their comments are below. The reviewers find that the paper has improved in revision, and therefore we'll be happy in principle to publish it in Nature Immunology, pending minor revisions to satisfy the referees' final requests and to comply with our editorial and formatting guidelines.

We will now perform detailed checks on your paper and will send you a checklist detailing our editorial and formatting requirements in a couple of weeks. Please do not upload the final materials and make any revisions until you receive this additional information from us.

If you had not uploaded a Word file for the current version of the manuscript, we will need one before beginning the editing process; please email that to immunology@us.nature.com at your earliest convenience.

Additionally, would you and your colleagues be interested in writing up a Research Briefing on your study? These pieces are a 'behind the study' descriptions from the authors viewpoint and would be published alongside the primary research article. Examples can be found at https://www.nature.com/ni/reviews-and-analysis?type=research-briefing&year=2026. If so, please let me know as I can contact the editorial team and put them in touch with you.

Thank you again for your interest in Nature Immunology Please do not hesitate to contact me if you have any questions.

Best regards,

Laurie

Laurie A. Dempsey, Ph.D.
Senior Editor
Nature Immunology
l.dempsey@us.nature.com
ORCID: 0000-0002-3304-796X

Reviewer #1 (Remarks to the Author):

The manuscript is much improved and clearer, with more in-depth mechanistic insights and important new analyses and experiments, including gain-of-function studies. Well done. I recommend it for publication.

Reviewer #3 (Remarks to the Author):

The authors have substantially revised this manuscript in response to the initial critiques, and I have not further comments. This is an important and novel contribution to the field.

*INTERNAL USE ONLY* AIP modules required: ["Life Science"]

**Open Access** This Peer Review File is licensed under a Creative Commons Attribution 4.0 International License, which permits use, sharing, adaptation, distribution and reproduction in any medium or format, as long as you give appropriate credit to the original author(s) and the source, provide a link to the Creative Commons license, and indicate if changes were made.
In cases where reviewers are anonymous, credit should be given to 'Anonymous Referee' and the source.
The images or other third party material in this Peer Review File are included in the article's Creative Commons license, unless indicated otherwise in a credit line to the material. If material is not included in the article's Creative Commons license and your intended use is not permitted by statutory regulation or exceeds the permitted use, you will need to obtain permission directly from the copyright holder.
To view a copy of this license, visit https://creativecommons.org/licenses/by/4.0/

We thank the reviewers for their critical and constructive feedback. We have performed copious additional experiments and analyses in response to the reviewers' comments to improve its mechanistic clarity. While there are still many more directions to pursue based on the findings of this study, we believe the revised manuscript has sufficient new advances and insights that would be valuable to the research community. In our point-by-point response to reviewers' comments, we have included revisions made to the manuscript in *dark blue italicized* font along with new and modified figure panels.

**Point-by-point response to reviewers**

**Reviewer 1 (comments to authors)**

The study presents a potentially novel finding regarding the role of the distal IRF4 element (OCRdist) in Treg destabilization. This finding is intriguing given prior studies on IRF4's role in Treg stability and Treg effector activity [PMID 32989329, 32125291]. However, the work remains preliminary and has significant limitations, particularly regarding in vivo relevance and mechanistic insight.

**R1, #1:** In vivo validation, such as data from human autoimmune conditions, would enhance the manuscript's impact.

Evaluating the chromatin accessibility status of IRF4 $OCR_{dist}$ in autoimmune disease patients is an interesting idea. We considered this but were concerned that such exploration might not be as informative as hoped. Our single-cell ATACseq data show that the opening of the IRF4 $OCR_{dist}$ occurred in exTregs that have lost lineage identity; thus, the cells of interest would not be among FACS-purified $CD4^{+}CD25^{+}CD127^{lo/-}$ Tregs. It is feasible to isolate Tregs from autoimmune patients and challenge the cells in the *ex vivo* destabilization model presented in this study. Such experiments may address the question of whether Tregs from autoimmune patients have altered tendency to become destabilized but will not address the *in vivo* function of *IRF4* $OCR_{dist}$ in human Treg stability that the reviewer is asking for. It may be possible to track Tregs in patients versus controls over time using TCR clonotype and monitor Treg stability and IRF4 $OCR_{dist}$ using multiomic analysis. This requires access to patient and control samples as well as dedicated technology to profile TCR and ATACseq that is not present accessible to us. We think this is best reserved in a future study dedicated to this issue. We have added the following to the first paragraph of the discussion (page 8):

> *This in vitro model of human Treg instability is yet to be tested in vivo. Tracking Treg TCR clonotypes [7] and epigenome over time in autoimmune versus normal controls may shed light on whether chronic inflammation contributes to human Treg lineage instability in vivo.*

Restricting the study to Treg adoptive cell therapy limits its broader applicability.

We agree and thank the reviewer for bringing up this point. We have rewritten the abstract and the introduction to focus on Treg biology. These sections are easy to locate in the revised document therefore are not pasted here.

**R1, #2:** The role of IL-6/IL-1β/IL-23 in Treg instability is already well-established, diminishing the novelty of these findings.

Although inflammatory cytokines including IL-6, IL-1β, and IL-23 have been reported to influence Treg stability, most prior studies were on mouse Tregs. Our choice of using this combination was based on unbiased comparisons of Th1, Th2, and Th17-driving cytokines, singly and in combinations, which demonstrated a stronger and more consistent Treg-destabilizing effect across many normal human donors (Fig. 1 and Extended Data Fig. 1). We are not aware of any prior report of human Treg destabilization using a combination of IL-6, IL-1β, and IL-23. It is also important to note that the human Tregs used in this study exhibited a high degree of lineage-stability during repeated *in vitro* stimulation and prolonged culture. This is in contrast to several prior reports that human Tregs lost lineage identity "spontaneously" during *in vitro* culture without the addition of inflammatory cytokines (PMIDs: 16917003, 19283780, 21593401, 25099355). Additionally, as discussed in our further responses below, we have distinguished some of the effects of the individual cytokines. We have revised the texts associated with Figure 1 and Extended Data Figures 1 and 2 to make it clearer our approach and rationale for selecting this cytokine combination to challenge human Tregs (page 2).

> *Previous studies on the impact of inflammatory cytokines on human Treg lineage stability have implicated a negative role of IL-12 [14, 15], whereas the effects of IL-6 have been more controversial [16, 17, 18, 19]. We thus started with unbiased comparisons of Th1, Th2, and Th17-driving cytokines, singly and in combinations (Extended Data Fig. 1c). We found that the Th17-promoting combination of IL-6, IL-1β, and IL-23 was most consistent at inducing FOXP3 and HELIOS silencing (Extended Data Fig. 1c).*

**R1, #3:** Furthermore, it is unclear how the tested conditions are physiologically relevant (e.g., concentration).

This is a fair question. Most inflammatory cytokines function in an autocrine or paracrine fashion within the local tissue environment where they are produced, making it difficult to determine their working concentrations *in vivo*. The best estimates we can think of are in tissue fluids such as synovium and cerebrospinal fluid (CSF) in patients with localized inflammation, and in plasma in patients with systemic inflammation. We have added an Extended Data Table 1 to the manuscript (pasted below) that summarizes a non-exhaustive literature survey of the concentrations of IL-6, IL-1β, and IL-23 reported in these settings. The survey shows that the concentrations we have used in our in vitro study have in vivo correlates under pathological conditions. Since the primary objective of our study was to investigate pathways that regulate human Treg lineage stability, we chose to conduct most of the experiments with higher concentrations of the inflammatory cytokines that provided a greater likelihood of capturing cells in the process of destabilization. The following texts has been added to the revised manuscript (page 2).

> *The fraction of FOXP3$^{-}$HELIOS$^{-}$ cells scaled with the concentration of exogenously added IL-6, IL-1β, and IL-23 across a 50-fold range (Fig. 1c), within the range reported in inflamed tissues in humans (Extended Data Table 1).*

> *Extended Data Table 1. Concentrations of IL-6, IL-1B, and IL-23 reported in patients with chronic inflammation*

| | **IL-6 (ng/ml)** | **IL-1β (ng/ml)** | **IL-23 (ng/ml)** |
|---|---|---|---|
| Synovium, RA | Up to 20 [56]<br>Up to 35 [57] | Up to 8 [57] | 5.19±1.8 [58] |
| Synovium, JIA | 2.6 – 3.0<br>± 0.5 - 1.3 [59] | 0.01-0.03 ± 0.5 - 1.3 [59] | |

| | | | |
|---|---|---|---|
| CSF, Neuroinflammation | up to 1 [60] | up to 0.15 [60] | |
| CSF, Bacterial meningitis | up to 6 [61] | | |
| Plasma, Septic shock | Up to 2,400;<br>Mean ~200 [62] | | |
| Ascites, Cancer | Up to >100;<br>Mean: ~50 [19]<br>0.4-80.5 [63] | | |
| Serum, kidney transplant | | | Up to 1.7 [9] |

**R1, #4:** The effect and mechanism of IRF4 OCRdist on Treg stability remains unclear and is constrained by significant gaps. What specific roles do the individual cytokines play, and how do they influence IRF4 OCRdist?

We agree that it would be helpful to know how individual inflammatory cytokine influences the chromatin accessibility at *IRF4* $OCR_{dist}$. We have performed new experiments by exposing human Tregs from 3-4 donors to either IL-1β, IL-6, or IL-23 individually, or in combination, during 3 rounds of repeated *in vitro* stimulation with anti-CD3 and anti-CD28 for 25 days. Flow cytometry and bulk ATAC-seq were performed on cells collected on Day 25. All individual cytokines led to less destabilization (higher % of $HELIOS^+$) compared to the cytokine combination. Among the individual cytokines, IL-23 induced the most destabilization, consistent with the results in Extended Data Figure 1 collected 5 years ago. Bulk ATACseq analysis shows that most of the changes in accessibility at the $OCR_{dist}$ were driven by IL-23. Furthermore, IL-23 also accounted for the majority of the change in genome-wide chromatin accessibility while IL-1β induced less changes and IL-6 had a minimal impact. We have added the following texts (page 5) and added new Fig 3d-f panels.

> *To determine the relative contribution of individual cytokines to the unraveling of the Treg epigenome, we purified Tregs from a separate cohort of donors and exposed the cells to IL-1β, IL-6, and IL-23 individually or in combination during 3 rounds of in vitro stimulation. Tconvs and Tregs cultured in IL-2 only without proinflammatory cytokines were included as controls. Cells were collected on day 25 for bulk ATAC-seq analyses. Principal component analysis based on genome-wide ATAC peaks revealed that Tregs exposed to combination cytokines clustered closely with those exposed to IL-23 only, whereas Tregs exposed to IL-1β or IL-6 clustered with Tregs cultured in IL-2 only. Both of these clusters were separated from the Tconv cluster (Fig. 3d). This result demonstrated that IL-23 accounted for the majority of chromatin accessibility changes in the combination cytokine condition. Tregs exposed to the combination of cytokines or IL-23 only showed closure of IRF4 $OCR_{prox}$ and opening of IRF4 $OCR_{dist}$, while IL-1β and IL-6 had a minimal effect (Fig. 3e). These data also confirmed an inverse correlation between accessibility at $OCR_{prox}$ and $OCR_{dist}$ among all treatment conditions (Fig. 3f). These results suggested that IL-23 had the most destabilization effect on human Tregs and the chromatin accessibility changes at IRF4 locus reflected a genome-wide reprogramming.*

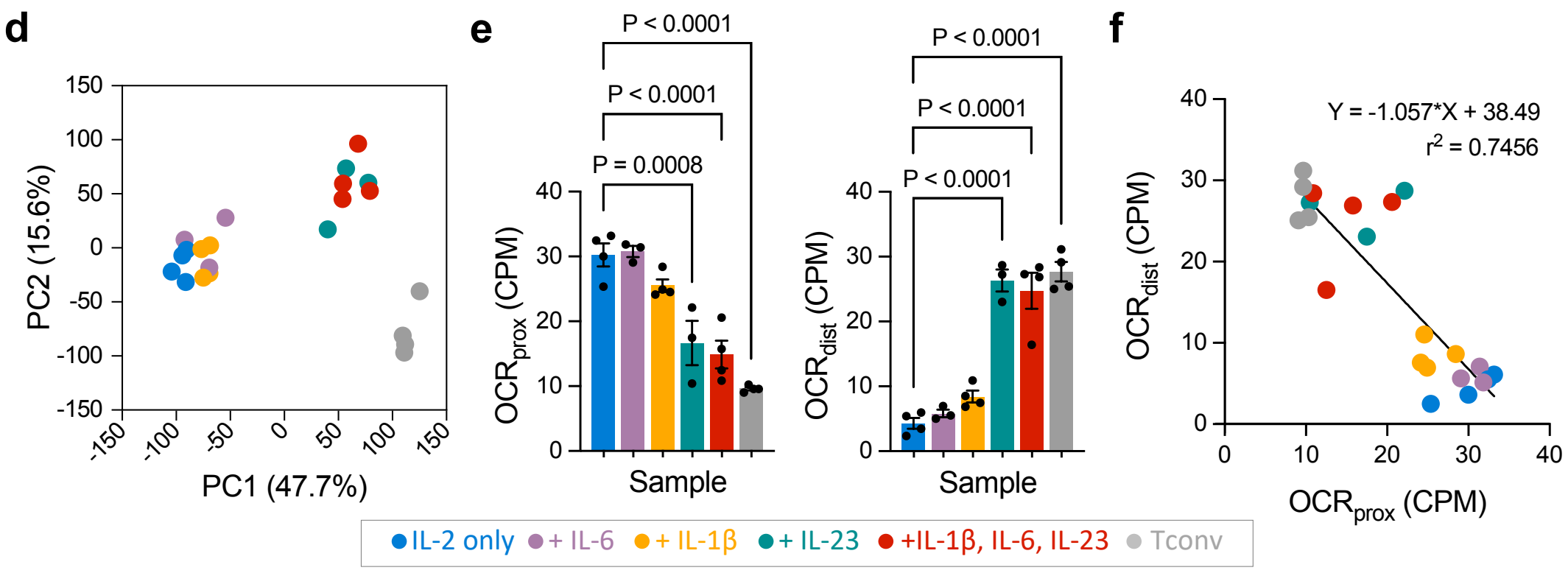


*__Fig. 3.__ … __(d)__ Bulk ATACseq was performed on Tregs treated with IL-2 only or IL-2 and one or more cytokines as indicated after 3 rounds of TCR stimulation; the PCA plot incorporating all ATAC peaks is displayed. __(e)__ Counts per million (CPM) at the ATAC peaks for $OCR_{dist}$ and $OCR_{prox}$ (n=3-4 donors per condition), statistical significance calculated via one-way ANOVA with Tukey's post test. __(f)__ Correlation of $OCR_{dist}$ and $OCR_{prox}$ accessibility among all samples, statistical significance calculated with simple linear regression model.*

**R1, #5:** What binds to this OCR?

This is another important question. During the revision, we focused on identifying transcription factors that drove Treg destabilization by interacting with *IRF4* $OCR_{dist}$. We have performed a series of new analyses and new experiments summarized below.

1. <u>Predicted TF binding to *IRF4* $OCR_{dist}$:</u> We scanned *IRF4* $OCR_{dist}$ for TF binding sites using FIMO (MEME Suite) and the JASPAR 2026 core vertebrates non-redundant motif database. We added the following texts (page 6) and new Fig. 6a to the revised manuscript.

   *$OCR_{dist}$ contained 33 TF motifs (Fig. 6a) targeted by AP-1, ETS, STAT, RUNX3, IRF, and members of several zinc-finger family TFs. The predicted AP-1, ETS and STAT binding sites were near the center of the ATAC-seq peak (~280bp, ~360bp, and ~400bp from N-terminus respectively), which suggested that occupancy by these TFs might dictate $OCR_{dist}$ chromatin accessibility.*

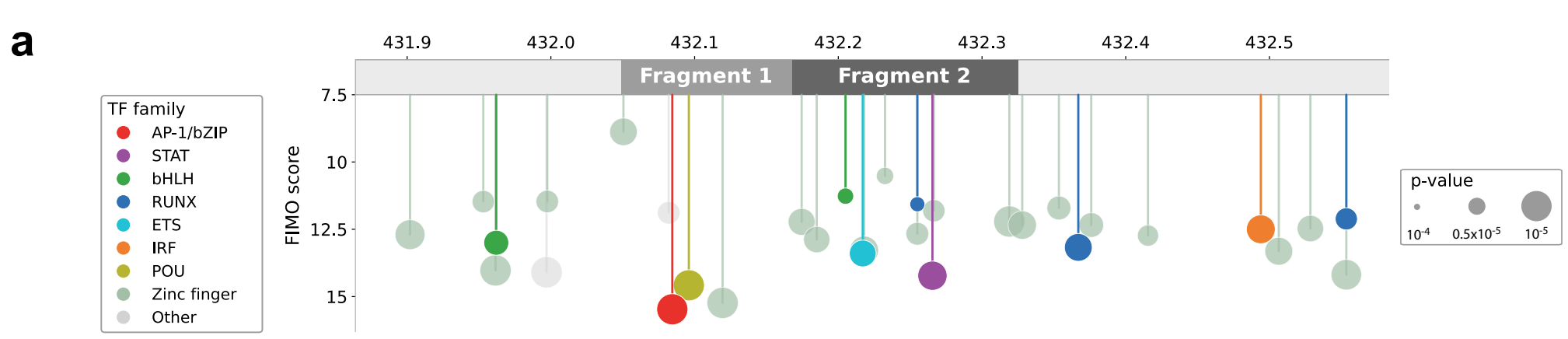


*__Fig. 6. (a)__ The $OCR_{dist}$ region was scanned for transcription factor binding sites found in the JASPAR 2026 database using FIMO (p-val < $1e^{-4}$). The 90 identified sites were classified by family and merged into 33 family-level binding sites, which are displayed. FIMO score is indicated by stem length and p-value is indicated by bubble size.*

2. <u>Genome-wide changes in TF site accessibility in cytokine-exposed Tregs:</u> To gain insights on what TFs are responsible increased chromatin accessibility, we next analyzed the new bulk ATACseq data for genome-wide TF accessibility changes. The analyses revealed significant increase in canonical AP-1, ETS, STAT and RUNX sites in cytokine-challenged Tregs when compared to Tregs cultured in IL-2 only. Notably, this analysis also revealed key differences

between the individual cytokines, contributing interesting insights to their role in Treg destabilization. Consistent with our group's previous reports that IL-6 enhanced Treg expansion without destabilization, IL-6 alone induced in minimal chromatin changes, while IL-1β unexpectedly decreased accessibility at ETS sites. Still, we found that most of the changes observed, including significant changes in canonical AP-1 (TRE site), RUNX, and STAT accessibility were due to IL-23. We added the following texts (page 6) and new Fig 6c to the revised manuscript.

> *Analysis of genome-wide TF motif accessibility revealed that the canonical AP-1 motif, TPA response element (TRE), was most significantly enriched in Tregs exposed to inflammatory cytokines compared to stable Tregs cultured with IL-2 alone but was not enriched in Tconvs (Fig. 6b). In contrast, RUNX, STAT, and ETS motifs were enriched in accessible chromatin in Tconvs and cytokines-exposed Tregs, suggesting opening these TF sites may be a late event during Treg destabilization, or a consequence rather than an initiator of Treg lineage loss. IL-23 alone altered TF site accessibly similarly to that seen in combination cytokine-treated Tregs. In contrast, IL-1β alone significantly increased AP-1 accessibility but significantly decreased accessibility of ETS sites, whereas IL-6 alone minimally changed accessibility to any TF sites profiled (Fig. 6b). Notably, GATA TF sites remained inaccessible in cytokine-treated Tregs, distinct from Tconvs, providing further evidence of destabilization rather than expansion of contaminant Tconv cells.*

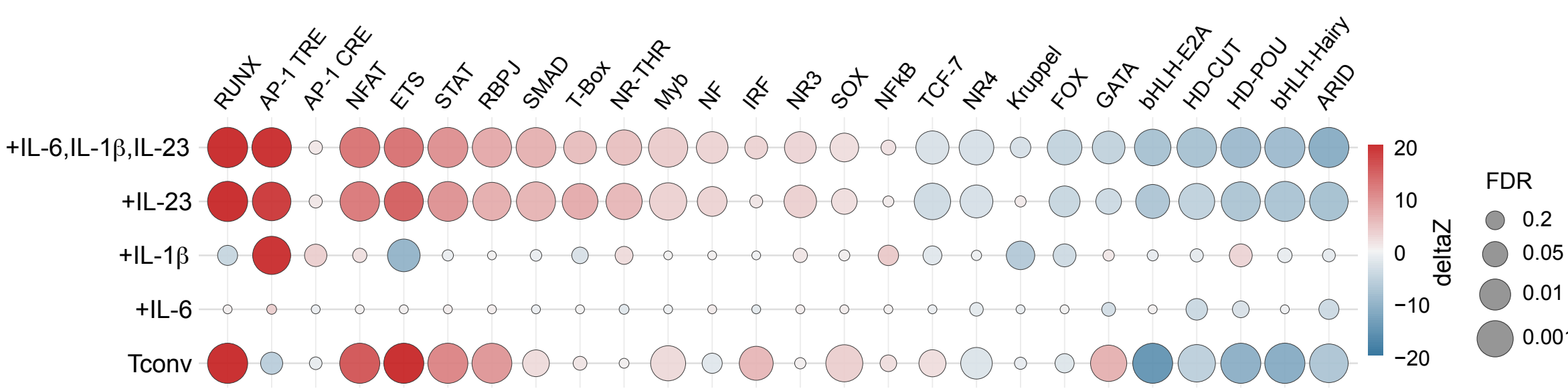


> ***Fig. 6. … (b)*** *Tregs were treated with IL-2 plus the indicated cytokines for 3 rounds of stimulation, and bulk ATACseq was performed. TF family motifs were curated from JASPAR 2026, and per-sample chromVAR z-scores were computed for the union peak set of each TF family. Significance was calculated using limma with Benjamini-Hochberg multiple testing correction. All comparisons are relative to the IL-2-only condition.*

3. <u>Define core region of the *IRF4* OCR$_{dist}$:</u> We designed additional sgRNAs to excise smaller regions of *IRF4* OCR$_{dist}$, and determined the effect on protecting Treg lineage identity during in vitro cytokine challenge. These new data have been added to the manuscript along with the following texts (page 6).

   *These results suggested that the central region in IRF4 ORC$_{dist}$ containing the AP-1, ETS, and STAT motifs might be important for driving Treg destabilization. We then designed additional sgRNAs to excise fragment 1 containing the AP-1 motif or fragment 2 containing ETS and STAT motifs (Fig. 6b) and subjected the edited Tregs to repeated stimulation in the presence IL-6, IL-1β, and IL-23. Deleting either of these fragments individually in Tregs conferred protection to a similar extent as the deletion of the entire OCR$_{dist}$ (Fig. 6c, 6d), suggesting that both AP-1 and ETS or*

*STAT binding sites of OCR$_{dist}$ are necessary for cytokine-induced Treg destabilization.*

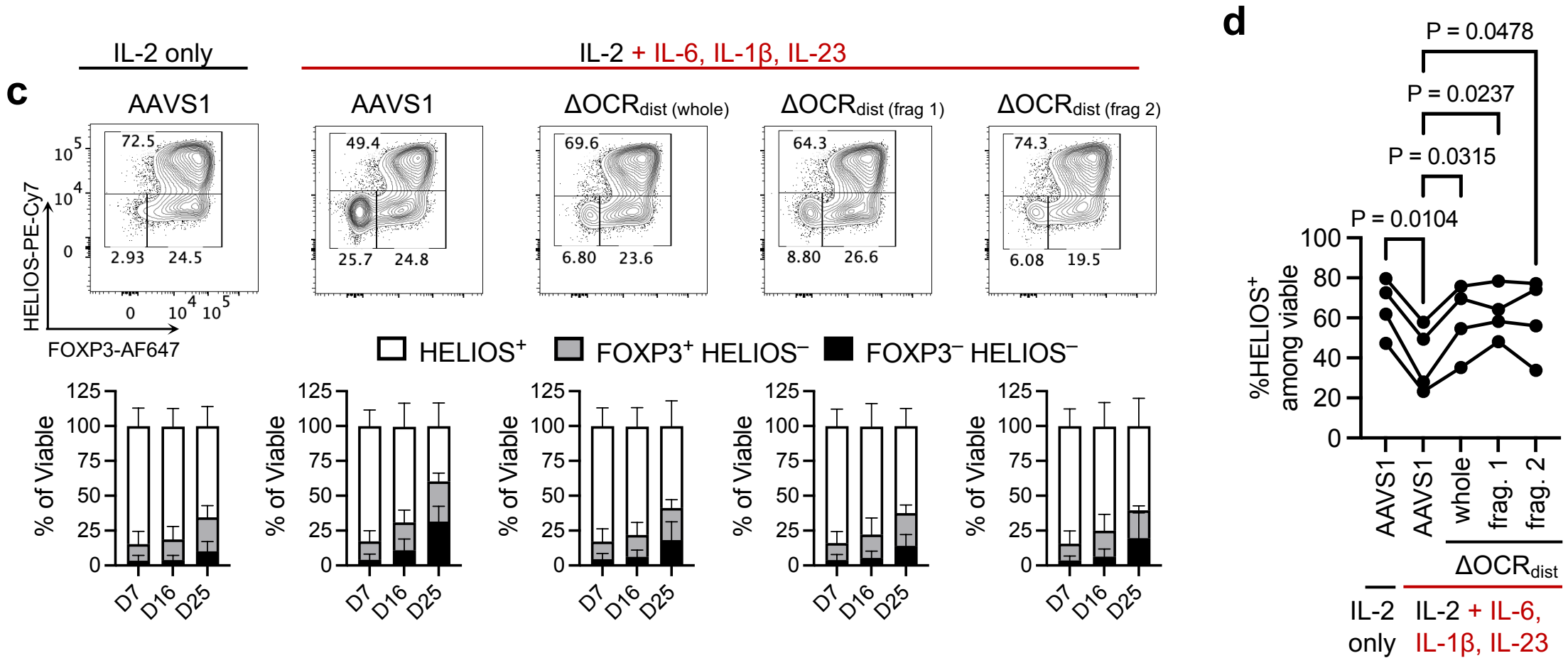


***Fig. 6. … (c)*** *Primary human Tregs were electroporated with CRISPR/Cas9 ribonucleoproteins (RNPs) designed to target AAVS1 or to excise either the whole OCR$_{dist}$ or portions of OCR$_{dist}$ (fragment 1, fragment 2, see Fig. 6) Tregs were subsequently challenged with repeated anti-CD3/CD28 stimulation and sustained IL-6, IL-1β, and IL-23 exposure to induce destabilization. Representative expression profiles for FOXP3 and HELIOS (top) and the summarized average distribution amongst HELIOS$^+$, FOXP3$^+$HELIOS$^-$, and FOXP3$^-$HELIOS$^-$ populations (bottom) at day 25 are shown.* ***(d)*** *Donor-matched comparison of the fraction of viable cells that remained HELIOS$^+$ by day 25 is summarized*

4. OCR$_{dist}$ enabled a feedforward mechanism for sustaining high IRF4 expression: Inflammatory cytokines induced genome-wide changes of chromatin accessibility. Yet, deletion only the IRF4 OCR$_{dist}$ had profound effect on Treg stability. We observed that the center ofIRF4 OCR$_{dist}$ contained IRF4-AP-1 and IRF4-ETS composite binding motifs, suggesting that IRF4 may enhance its own expression by cooperating with AP-1 and ETS TFs. We have added a new Fig. 7a and the following texts (page 6-7) to the manuscript:

   *Our data thus far showed that proinflammatory cytokines increased accessibility to AP-1, ETS, STAT binding sites and altered chromatin landscape in Tregs. Moreover, IRF4 OCR$_{dist}$ accessibility, particularly the region containing AP-1, ETS, and STAT-binding sites, was necessary for Treg destabilization, likely through increasing IRF4 expression. This raised the question of whether IRF4 was responsible for reprograming the Treg cell fate. Notably, IRF accessibility was not significantly altered by cytokine exposure (Fig. 6b). While IRF4 can function as a homodimer by binding to interferon-stimulated response elements (ISRE), it can also be recruited to AP-1–IRF composite elements (AICEs) and ETS–IRF composite elements (EICEs) (Fig. 7a). We analyzed OCR$_{dist}$ and identified one AICE, one ISRE, and five EICE sites (Fig. 7b), suggesting IRF4 may enhance its own expression via partnership with AP-1 and ETS TFs at OCR$_{dist}$. This constituted a feedforward mechanism to reinforce OCR$_{dist}$ accessibility and sustain high IRF4 expression, implying that the opening of IRF4 OCR$_{dist}$ might be a point of no return in Treg lineage decommitment.*

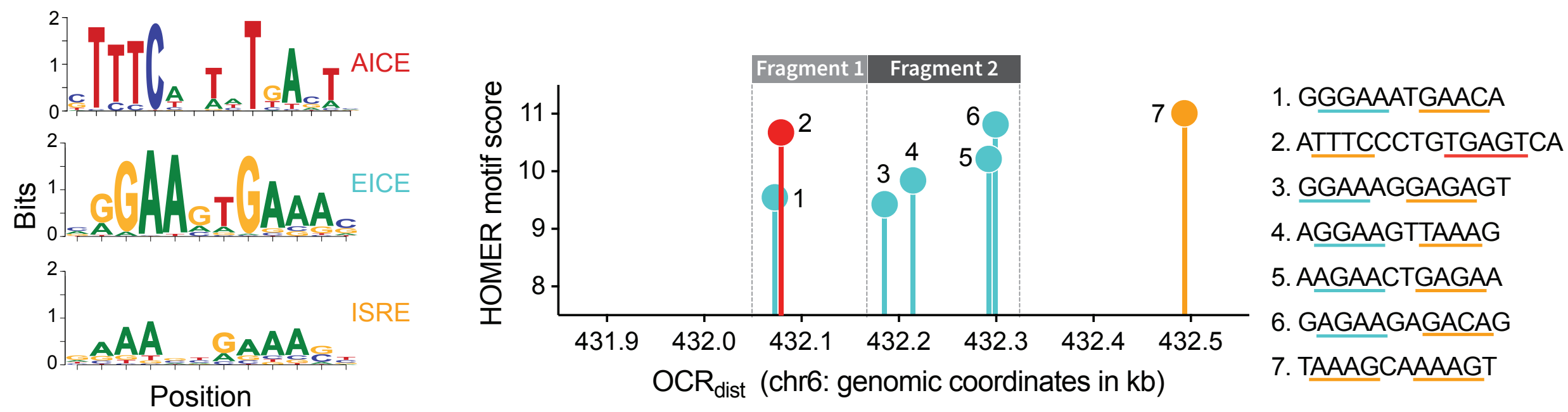


***Fig. 7. (a)** Motifs for AP-1-IRF4 composite element (AICE), ETS-IRF composite element (EICE), and Interferon-stimulated response element (ISRE). (**b**) Summary of AICE, EICE, and ISCE elements found within IRF4 OCRdist using HOMER.*

5. Cooperation between IRF4 and AP-1 in driving Treg destabilization: IRF4 and AP-1 are both increased in cytokine-exposed Tregs. We thus hypothesized that IRF4 and AP-1 together cooperatively drive Treg destabilization. We overexpressed IRF4 and BATF (an AP-1 family member) singly or in combination using CRISPRa and observed consistent Treg destabilization when both were overexpressed. New texts (page 7) along with new Fig 7b detailing these results have been added to the revised manuscript.

*To test our hypothesis that increased abundance of AP-1 and IRF4 in cytokine-exposed Tregs drives Tregs destabilization, we used a CRISPRa system to overexpress IRF4 and the AP-1 factor BATF alone or in combination and assessed the impact on Treg stability during repeated stimulation in the absence of inflammatory cytokines. We chose BATF because it is known to partner with IRF4 to form a high-affinity binding complex at AICE sites [28, 29]. Tregs dually transduced with lentiviral vectors containing CRISPRa and sgRNA targeting IRF4 and/or BATF were FACS purified and validated for overexpression by flow cytometry (Fig. 7c). At the end of the fourth stimulation cycle, the proportion of HELIOS$^+$ cells was unchanged by BATF overexpression alone, variably decreased by IRF4 overexpression, and consistently and significantly decreased by dual overexpression of IRF4 and BATF (Fig. 7d, 7e). Thus, the effect of cytokine-driven Treg destabilization could be partially recapitulated via overexpression of both IRF4 and BATF, supporting a model of IRF4 and AP-1 partnership as one driver of Treg destabilization.*

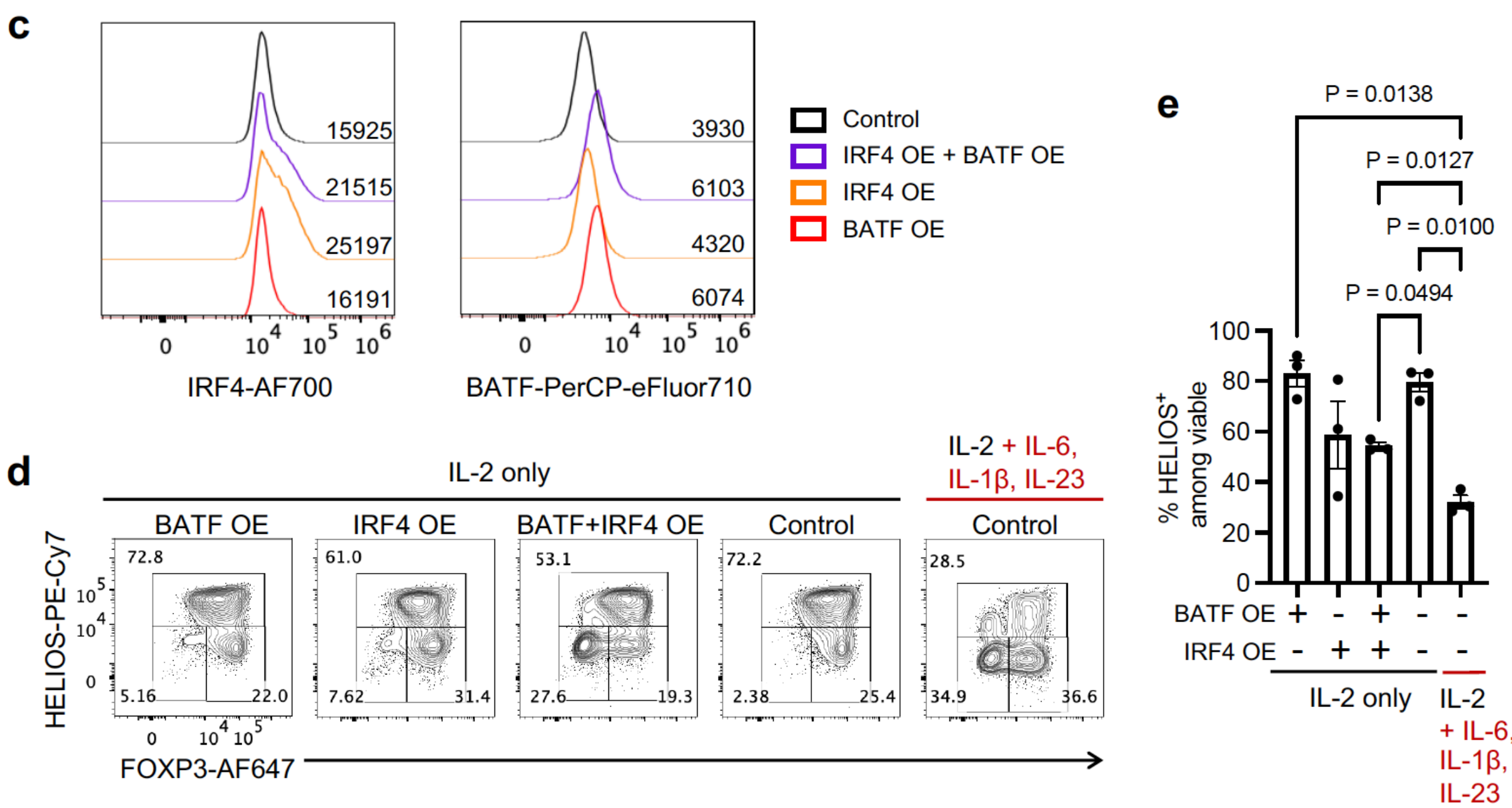


*Fig. 7. … (c, d, e) Primary human Tregs were treated with lentivirus containing CRISPRa machinery and an sgRNA targeting either BATF, IRF4, a combination of these two sgRNAs, or a non-targeting control sgRNA. Transduced cells were sorted and Tregs were subsequently challenged with repeated anti-CD3/CD28 stimulation with IL-2 only (except for positive control treated with IL-6, IL-1β, and IL-23). Representative histograms with geometric mean fluorescence intensity for BATF, IRF4 on day 24* ***(c)*** *and representative plots with gated proportions for FOXP3/HELIOS on day 38* ***(d)*** *are shown.* ***(e)*** *Donor-matched comparison of the fraction of viable cells that remained HELIOS+ by day 38 is summarized. Statistical significance was calculated via paired one-way ANOVA with Tukey post test.*

**R1, #6:** Is IRF4 OCRdist independent of previously identified Treg stabilization mechanisms such as TSDR, CNS2? Additionally, since IRF4 has been shown to promote FOXP3 expression, how it contributes to Treg destabilization in this context requires further clarification.

Our study suggests that high expression of IRF4 is detrimental to Treg lineage stability. IRF4 is not known to directly bind to CNS2 or other regulatory regions at the *FOXP3* locus, thus it likely functions indirectly to alter CNS2 methylation. These molecular mechanisms remain to be investigated. The role of IRF4 in FOXP3 expression and in effector Treg differentiation have mostly been shown in the context of IRF4 deletion. Our results do not dispute these prior conclusions but further suggest that quantitative changes in IRF4, perhaps beyond homeostatic levels, drive divergent Treg cell fate. We have revised the discussion to clarify these points (page 8):

> *Several non-mutually exclusive mechanisms may explain how a quantitative change in IRF4 can alter Treg cell fate. IRF4 and its partners are not known to target the FOXP3 locus [31], thus its effect on FOXP3 silencing and Treg instability is likely indirectly regulated. IRF4 is essential for TCR-induced proliferation and survival of CD8$^{+}$ [32, 33] and CD4$^{+}$ effector T cells [34, 35], whereas limiting IRF4 activity or deletion of IRF4 favor iTreg induction [36]. Thus, an OCR$_{dist}$-dependent increase of IRF4 expression in (ex)Tregs may support the survival and/or proliferation of (ex)Tregs, and the overall enhancement in Treg stability mediated by OCR$_{dist}$ deletion may be due to decreased persistence of (ex)Tregs. This interpretation is supported by the increased proliferation and accumulation of (ex)Tregs in Treg*

*cultures exposed to inflammatory cytokines. Alternatively, increased IRF4 expression may directly contribute to chromatin remodeling in Tregs. IRF4 is a well-established mediator of effector Treg programs, which is cooperatively controlled with FOXP3 both transcriptionally and via protein-protein interaction [26, 37]. Increased IRF4 abundance may supersede FOXP3 partnership, enabling alternative transcriptional programs by IRF4 recruitment to low-affinity binding sites [34] or heterodimerization with other partners [25]. These aberrant IRF4 activities may drive epigenetic rewiring that ultimately unravels Treg lineage identity. In this regard, IRF4 is reported to interact with chromatin-modifying complexes, NuRD, SWI/SNF, and SATB1 [31] that can lead to genome-wide changes gene expression and chromatin accessibility.*

*Prior deletional studies have demonstrated a role of IRF4 in sustaining FOXP3 expression in human Tregs [16] and in effector Treg differentiation in mice and humans [37, 38]. Findings from this study further suggest that excessive IRF4 expression promotes Treg destabilization. Therefore, IRF4 abundance and activities in Tregs need to be tightly controlled to support effector Treg function without disrupting Treg identity. In this regard, Bach2 and Blimp1 repress IRF4 activity after TCR activation in Tregs [36, 39]. Our results further suggest that the amount of IRF4 expression in Tregs is epigenetically controlled by restricting chromatin accessibility at IRF4 $OCR_{dist}$. This intricate module is vulnerable to the influence of inflammatory cytokines, particularly IL-23. The pronounced enrichment of AP-1 binding sites in inflammatory cytokine-exposed Tregs implicated a role of this family of TFs in mediating the effect of proinflammatory cytokines to reshape the Treg epigenome. The crucial importance of IRF4 $OCR_{dist}$ in governing Treg lineage identity may be due to its control of IRF4 abundance and partnership with AP-1 and ETS TFs. Collectively, our data are consistent with a model in which cytokine-induced AP-1 upregulation initiates genome-wide chromatin alterations, including at IRF4 $OCR_{dist}$, leading to increased IRF4 expression. IRF4, in turn, partners with AP-1 to solidify chromatin changes and drive Treg lineage decommitment. This model requires further experimental validation.*

**R1, #7:** Is it possible that this OCR controls other loci than IRF4 (DUSP22 is in the proximity and affect TCR signaling [PMID36765305,38225265])?

The peak-gene expression linkage analysis in the original Fig 4c was performed with our scMultiome data. It showed strongest correlation between *IRF4* $OCR_{dist}$ accessibility with IRF4 expression and also a weaker correlation with *DUSP22* expression (not shown in original Fig 4c). We have now performed additional analysis to include surrounding loci including *DUSP22*, and *EXOC2* using published human Tconv promoter-capture Hi-C that supported this conclusion. We have added this data to the new Extended Data Fig. 5 and associated texts (page 5) to show this expanded analysis.

*Analysis of published promoter-capture Hi-C data in activated T cells [28] also revealed a strong physical association between $OCR_{dist}$ and the IRF4 promoter, a weaker association with the promoter of DUSP22, and no association with EXOC2 (Extended Data Fig. 5a). IRF4, but not EXOC2 or DUSP22, expression was positively correlated with $OCR_{dist}$ accessibility, further supporting a regulatory role of $OCR_{dist}$ in IRF4 expression (Extended Data Fig. 5b).*

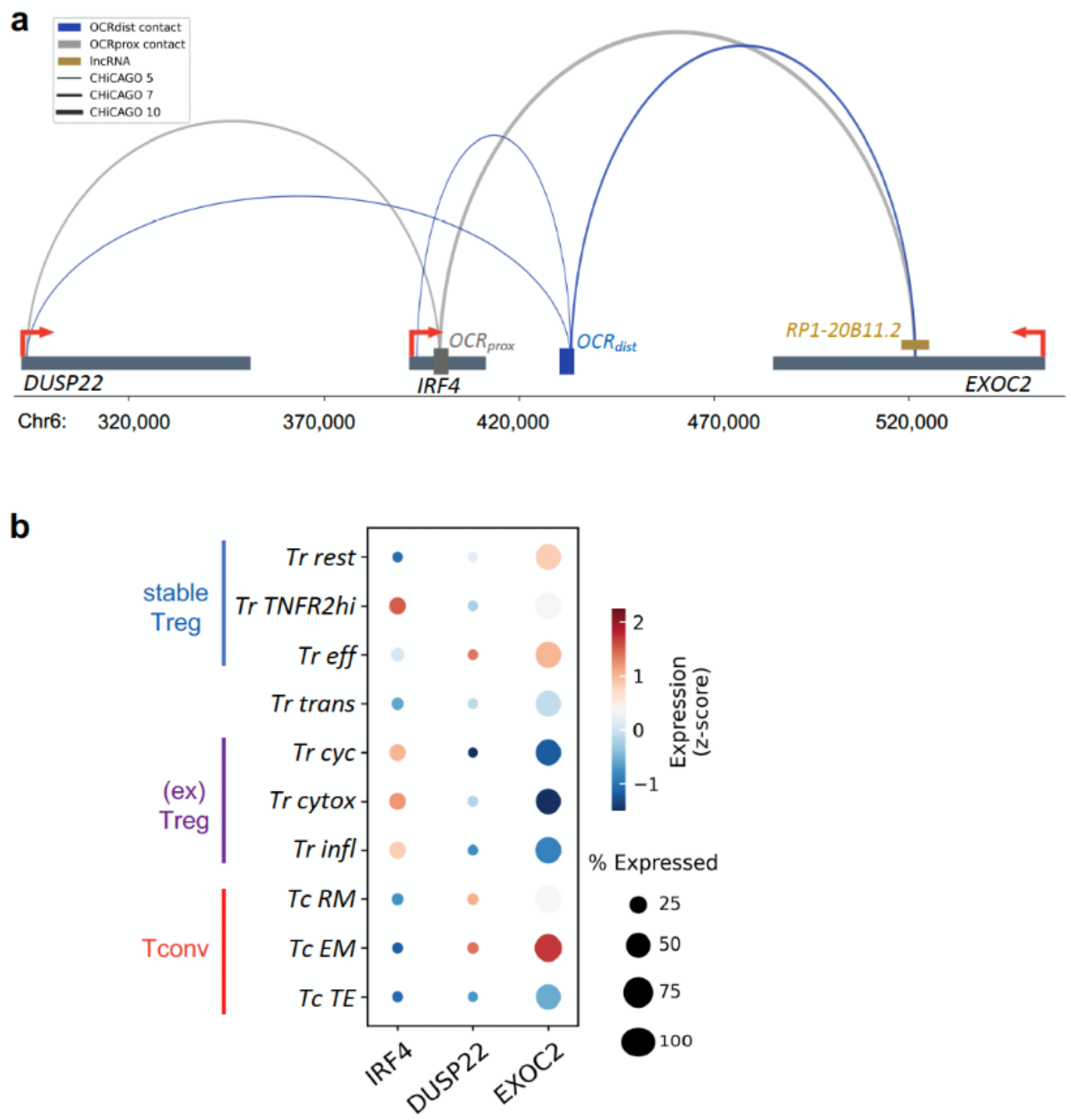


***Extended Data Fig. 5. OCR$_{dist}$ is linked to IRF4 at the chromatin level. (a)*** *Analysis of promoter capture Hi-C data from ref. 43 in the vicinity of IRF4. Linkage significance was measured by CHICAGO score, peak height and thickness represent higher CHICAGO score.* ***(b)*** *Expression of IRF4 and neighboring genes DUSP22 and EXOC2 in the clusters defined in Fig. 2 in the scRNAseq dataset.*

**R1, #8:** The reliance on in vitro models raises concerns about artifacts, and interpretation of the experiments is open to alternative interpretations. The 2^15-fold expansion amplifies even minor contaminants to potentially significant levels.

We understand the reviewer's concern. We had similar concerns during our research. Throughout the investigation, we have scrutinized the data for this confounding issue. We think the likelihood of a low frequency of Tconv impurities contributing to the emergence of the FOXP3$^{-}$HELIOS$^{-}$ population in cytokine-challenged Tregs is low based on the following observations:

1. Tregs cultured under control conditions are remarkably stable throughout repeated stimulations over the 25-day period (Fig. 1). This shows our Treg isolation protocol yields highly pure Tregs. Our Treg isolation protocol involved MACS pre-enrichment for CD4$^{+}$ or CD25$^{+}$ cells from PBMC, followed by FACS purification. During FACS, we conservatively gate on the CD25$^{+}$CD127$^{-/low}$ population, which is well-separated from Tconv cells, especially after the CD25 enrichment. We have added an example of our Tregs FACS gating (Extended Data Fig. 1a).

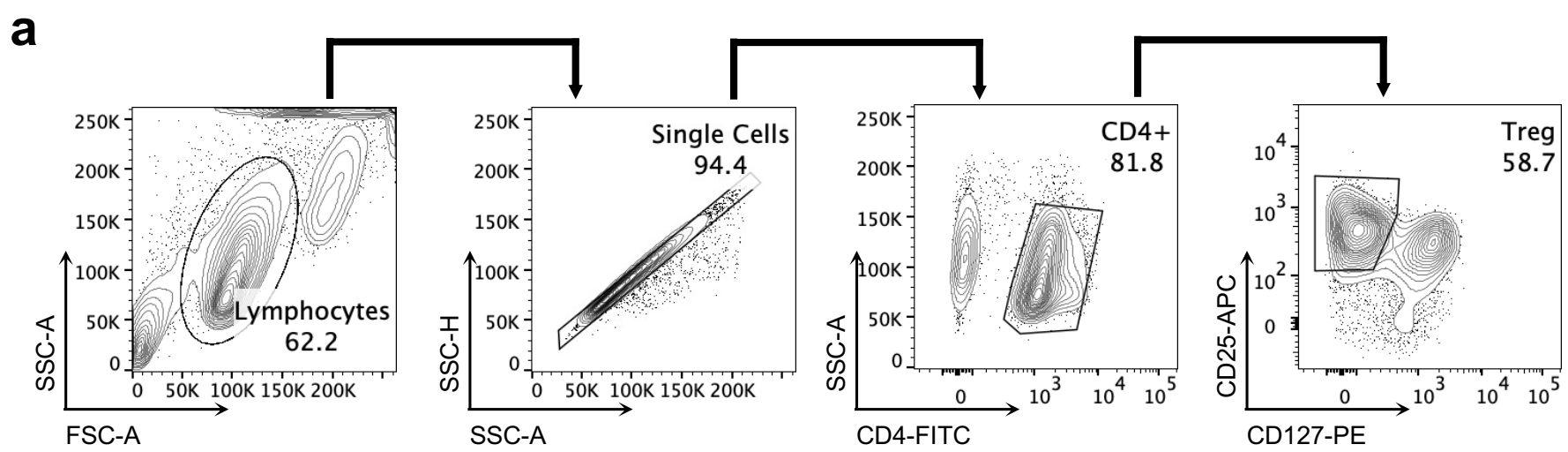


***Extended Data Fig. 1a:*** *An example of FACS gating strategy for CD4⁺CD25⁺CD127⁻/low Tregs from CD25 pre-enriched human PBMC.*

2. FOXP3⁻HELIOS⁻ cells from IL-6, IL-1B, IL-23–treated cultures exhibit a phenotype that is intermediate between stable Tregs and Tconv cells. For example, FOXP3⁻HELIOS⁻ cells have ~30% TSDR demethylation, significantly higher than Tconvs (Fig. 1d and Extended Data Fig. 3). If the FOXP3⁻HELIOS⁻ population in the IL-6, IL-1β, IL-23–challenged cultures were the result of Tconv contamination, we would expect TSDR demethylation to be identical to that of Tconvs since *in vitro* culture does not induce TSDR demethylation in Tconvs.
3. single-cell Multiome UMAP (based on ATAC and GEX data) showed minimal overlap between (ex)Tregs and Tconvs (revised Fig 2a and 3a, combined below).

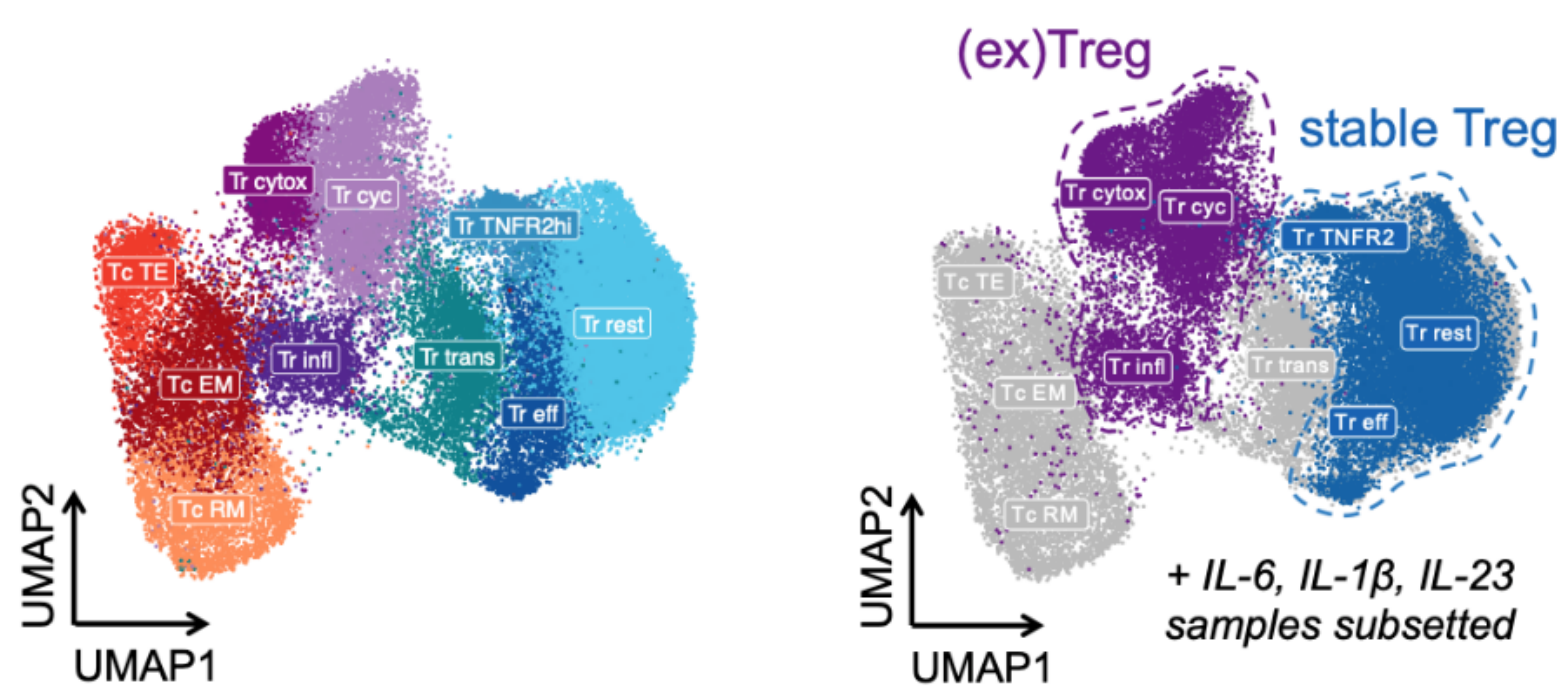


Fig. 2a. Unsupervised clustering on a weighted nearest neighbor (WNN) UMAP of 10x scMultiome data.

Fig. 3a. stable Treg and (ex)Treg clusters from IL-6, IL-1β, and IL-23 treated samples

4. Expression levels of Treg signature markers, e.g. CD25, CTLA4, ICOS, TIGIT, etc., decreased in the FOXP3⁻HELIOS⁻ population, but remained significantly higher than Tconvs, suggesting an intermediate cell state. Similarly, the proportion of CD40L-expressing cells increased within the FOXP3⁻HELIOS⁻ population but did not reach the percentage nor the expression level observed in Tconvs (original Fig. 2a, Extended Data Fig. 3). To further clarifying this point, we have added statistical analyses to revised Extended Fig 3b and re-plotted the flow data in revised Extended Data Fig. 3c to focus on the FOXP3$^{low/–}$ subpopulation within the HELIOS⁻ fraction of cells and compare phenotypic markers profile to Tconvs.

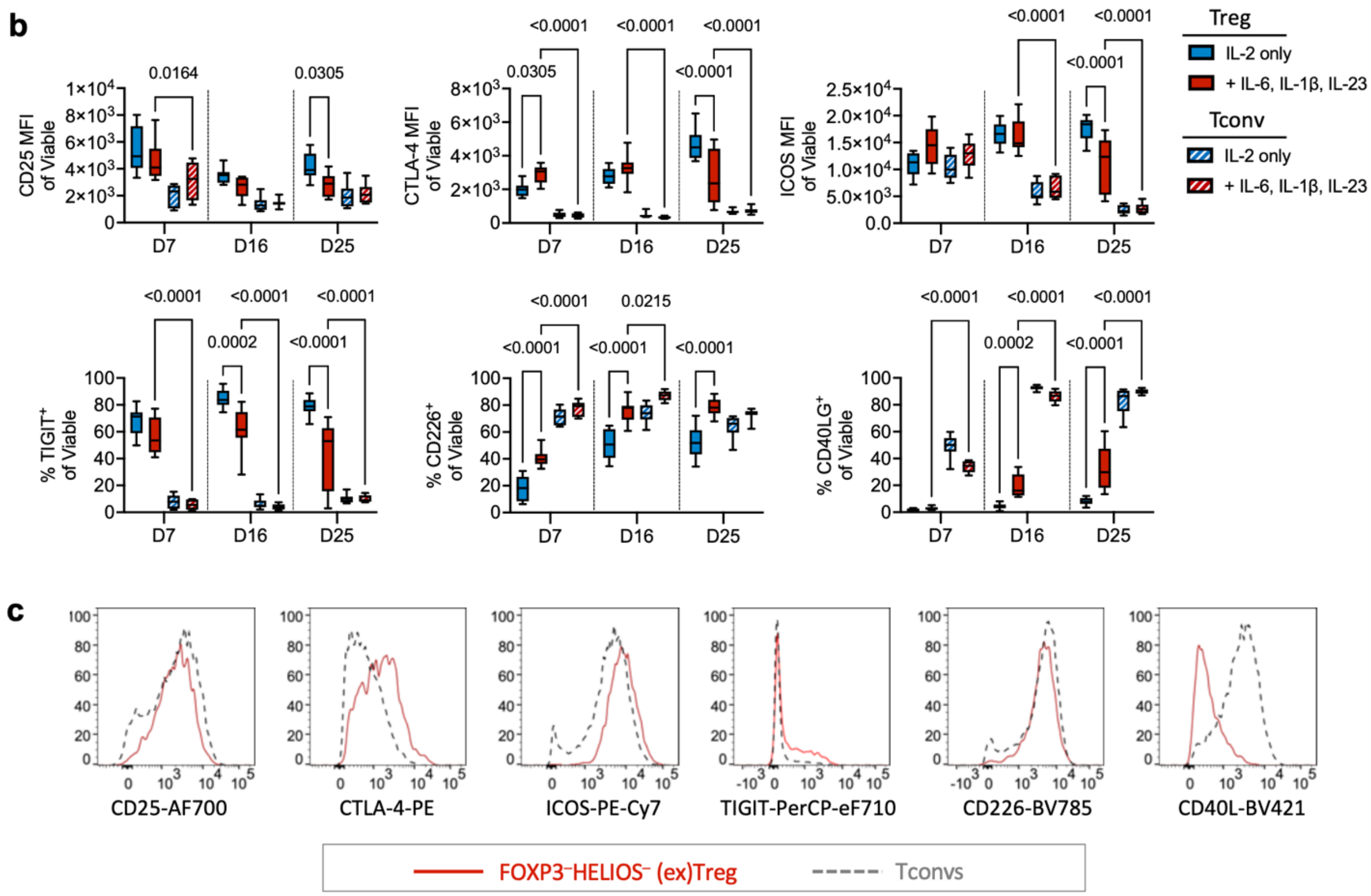


***Extended Data Fig. 3. Inflammatory cytokine exposure induces dysregulated expression of Treg phenotypic markers (corresponding to Figure 1). … (b)*** *Average geometric mean of fluorescence intensity (MFI; CD25, CTLA-4, ICOS) or percent positive (TIGIT, CD226, CD40LG) of common Treg phenotypic markers is summarized at days 7, 16, and 25 for each condition.* ***(c)*** *Histograms for phenotypic markers shown in (a), from the gated FOXP3⁻HELIOS⁻ population within the Treg (solid red line) and Tconv (dashed black line) samples cultured in the presence of IL-2 and IL-6, IL-1β, and IL-23.*

5. Bulk ATAC-seq analysis showed that Tregs treated with inflammatory cytokines, particularly IL-23, clustered away from both stable Tregs and Tconvs (revised Fig 3d). Analysis of specific TF families shows that the increased accessibility at AP-1 sites was unique to exTregs after inflammatory cytokine treatment and not shared with Tconv (Fig 6c). On the other hand, Tconv uniquely showed high accessibility at GATA TF sites, which is not seen in cytokine-challenged Tregs.

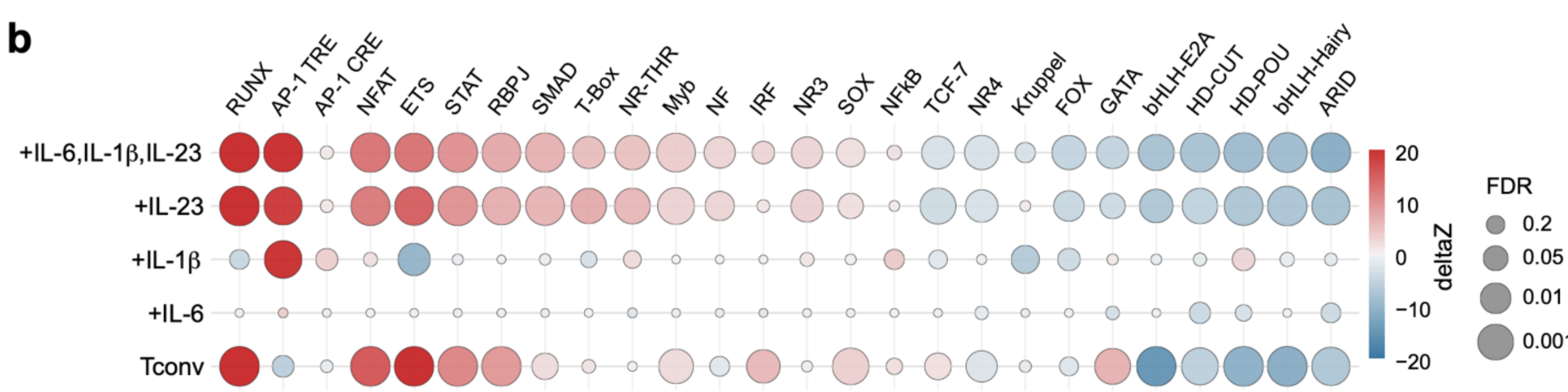


***Fig. 6. …(b)*** *Tregs were treated with IL-2 plus the indicated cytokines for 3 rounds of stimulation, and bulk ATACseq was performed. TF family motifs were curated from JASPAR 2026, and per-sample chromVAR z-scores were computed for the union peak set of each TF family. Significance was calculated using limma with Benjamini-Hochberg multiple testing correction. All comparisons are relative to the IL-2-only condition.*

Together, these data gave us confidence that the FOXP3⁻HELIOS⁻ cells were indeed destabilized Tregs, not from minor contaminants of Tconvs that grew out during extended in vitro culture.

The small effect size of IRF4 OCRdist knockout (figure 5), the confinement of observed effects to FOXP3-HELIOS- cells, combined with the finding that OCRdist is open in Tconv and closed in Tregs, raises the question of whether OCRdist amplifies or stabilizes a small population of Tconv/exTregs rather than destabilizing Tregs as a whole. Additional experiments would greatly strengthen the manuscript. For example, analyzing FOXP3-low cells, measuring absolute cell counts in vitro and employing single-cell RNA sequencing combined with TCR sequencing as fate-mapping tools would address several open questions.

By small effect size in Fig 5, we believe the reviewer is referring to the representative flow plots in the original Fig. 5e (revised to Fig 4e) showing lower expression of IRF4 in $FOXP3^{-}HELIOS^{-}$ cells. Since flow histograms are displayed on a log scale, it can be difficult to discern changes. Fig. 4f provides a summary of the results in Fig. 4e, which show a significant and strong reduction of IRF4 expression. We have revised Fig. 5e (revised Fig 4e) to overlay of histograms, instead of stacked histograms, to make it easier to appreciate the difference in IRF4 expression.

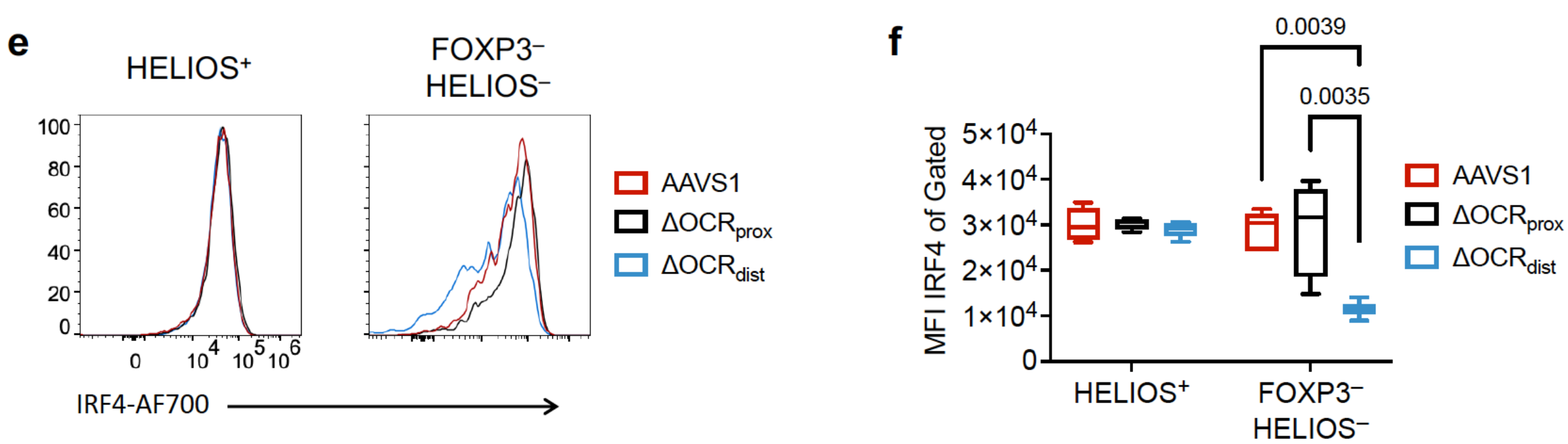


***Fig. 4. …(e)*** *CRISPR/Cas9 ribonucleoprotein complexes were delivered via electroporation to excise $OCR_{prox}$ and $OCR_{dist}$ prior to inflammatory cytokine exposure with IL-6, IL-1β, and IL-23. Representative histograms for IRF4 expression in $HELIOS^{+}$ (stable) and $FOXP3^{-}$ $HELIOS^{-}$ (destabilized) sub-populations from AAVS1-edited, $ΔOCR_{prox}$, and $ΔOCR_{dist}$ Tregs at Day 20 of in vitro culture.* ***(f)*** *Geometric mean fluorescence intensity of IRF4 expression (n = 6 normal human donors) for the samples in (e) is summarized. Statistical significance was calculated via one-way ANOVA with post Tukey test.*

We would also like to point out that the impact of IRF4 $OCR_{dist}$ deletion is not limited to $FOXP3^{-}$ $HELIOS^{-}$ cells. Our data in original Fig. 6 (revised to Fig 5) show that IRF4 $OCR_{dist}$ deletion also increases FOXP3 expression in $HELIOS^{+}$ (still stable Treg) cells (new Fig. 5c, 5d). Thus, there is evidence that removal of IRF4 $OCR_{dist}$ may contribute to both the (1) IRF4 expression of already destabilized cells and (2) elevated FOXP3 expression and increased stability of still stable Tregs.

The idea of using TCR to track Tregs during *in vitro* culture is attractive. The technology to simultaneously profile single-cell gene expression (GEX), chromatin accessibility (ATAC), and TCR repertoire was not offered by 10X at the time we conducted our single-cell multiomic experiments (still not a standard product to the best of our knowledge). We will need all three modalities to define Treg stability of individual clones over time. We thus did not pursue this direction as part of this study.

**R1, #9:** Furthermore, a mouse model with IRF4 OCRdist deletion, combined with Treg fate mapping tools in autoimmune models, would provide valuable insights. It is also worth exploring whether IRF4 OCRdist is present in mice and functions similarly.

We have performed computational analysis on the alignment between the human *IRF4* $OCR_{dist}$ and other vertebrates. The analysis revealed a high degree of conservation among primates and variable with other species (Rebuttal Fig. 1). Specifically, the similarity between mouse and human in this region is 42.9%, which is calculated based on both similarity of aligned sequences and gaps in alignment.

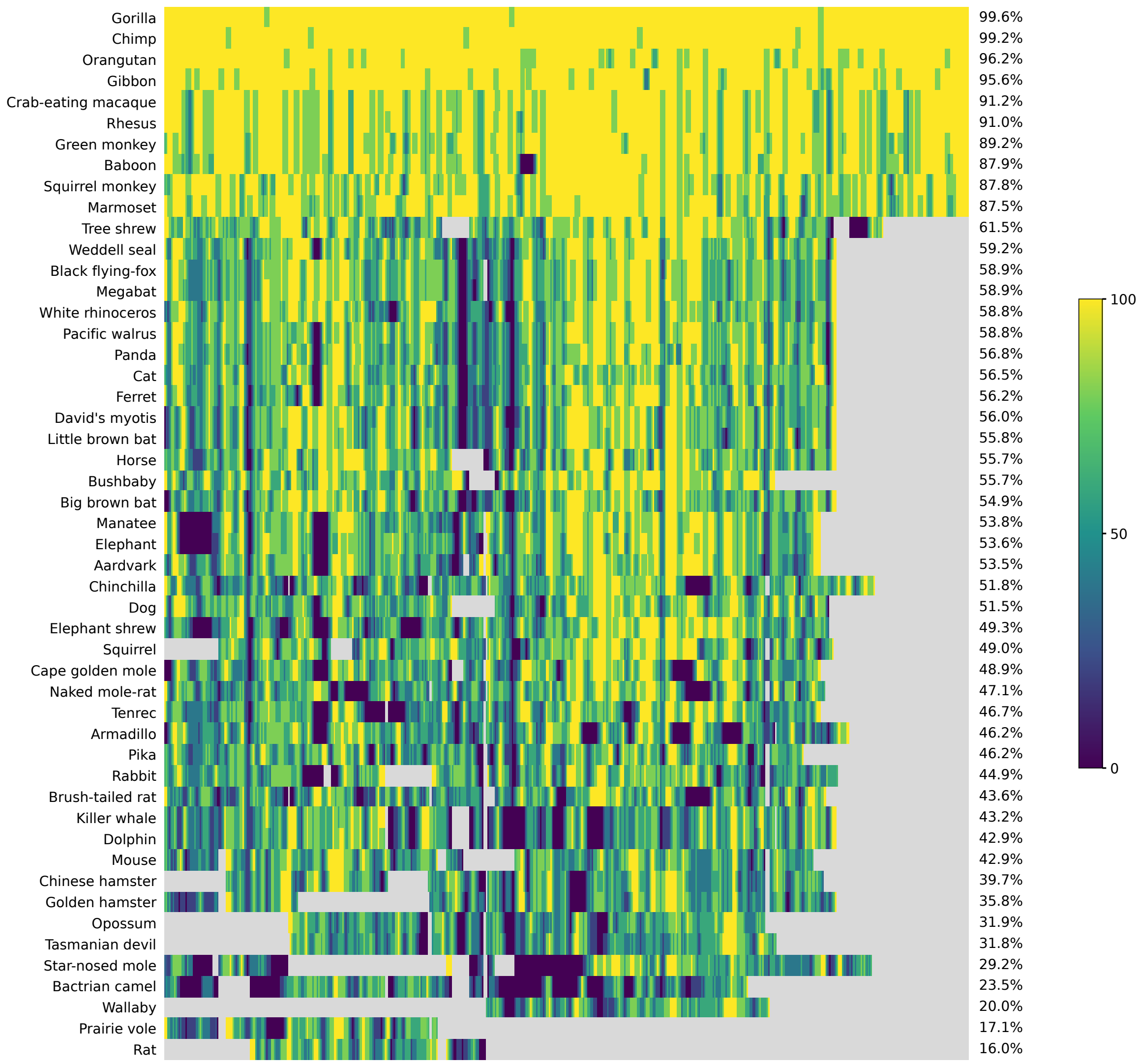


**Rebuttal Fig. 1. Heatmap showing similarity of *IRF4* $OCR_{dist}$ among vertebrate animals relative to *Homo sapiens.*** Similarity scores (right) were calculated using UCSC 100-way vertebrate alignment and shown as heatmap over the $OCR_{dist}$ region. The light grey space indicates gaps in alignments.

Our results with human Tregs suggest that the AICE element is likely important. A closer look of the alignment at the AICE site shows imperfect match with the AICE motif in the mouse sequence (Rebuttal Fig. 2). It is hard to predict if mouse has an analogous *Irf4* $OCR_{dist}$ based on these analyses. Published reports have highlighted key differences in Tregs between mice and humans, including in the expression of genes important Treg function (PMID: 39196959, 41237776). We

hope the reviewer agrees that whether *IRF4* $OCR_{dist}$ regulates mouse Treg lineage stability is a separate line of research best reserved for future studies.

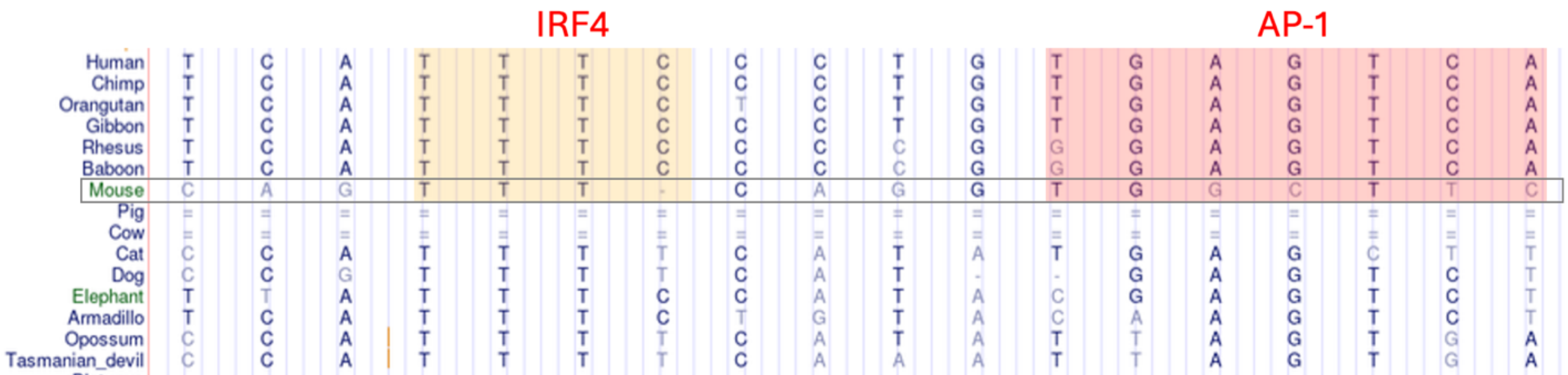


**Rebuttal Fig. 2**. **Alignment of AICE site in *IRF4* $OCR_{dist}$** showing divergence between primates and mouse.

**R1, #10:** Finally, the manuscript appears incomplete. Methodological details are insufficient, statistical analyses—particularly for genomics—are minimal, and several key figure panels (1d, 5c, and 6a) are missing.

We thank the reviewer for the feedback on the need for methodological details. We have enlisted a bioinformatician (new co-author V.N.) to review all analyses, statistical methods, and languages for genomics data including previous scRNA+ATAC-seq and new bulk ATAC-seq data. We have provided enhanced details for all experimental and bioinformatic analyses throughout the entire methods section. Due to the extensive changes and additions, they are not pasted in this document, but are all highlighted in the revised manuscript.

**Reviewer 3 (comments to authors)**

In their study, Ho et al. develop an in vitro system to model the loss of Treg stability driven by inflammatory cytokine exposure that is a major barrier to the successful implementation of adoptive Treg cell therapy to promote tolerance in autoimmunity and transplantation. They demonstrate that repeated stimulation of purified human Treg in the presence of an inflammatory cytokine cocktail leads to loss of Foxp3 and Helios expression associated with remethylation of the Treg-specific Foxp3 TSDR. Destabilized Treg were functionally less suppressive and instead acquired the ability to produce pro-inflammatory cytokines. Single cell multi-omic analyses identified transcriptional and epigenetic changes associated with de-stabilized Treg, including a shift in the relative accessibility of two open chromatin regions in the IRF4 locus from an intronic proximal OCR (OCRprox) to a distal OCR (OCRdist) that is normally closed in Treg cells but open in conventional T cells. They further show that the OCRdist controls TCR-mediated upregulation of IRF4, and most importantly that CRISPR-mediated deletion of OCRdist promotes Treg stability during expansion in the presence of inflammatory cytokines and enhances regulatory activity in a xenograft model of GVHD.

Overall, this is a well-performed study with many strengths. The identification of an open chromatin region that regulates Treg stability in the face of inflammatory cytokine treatment is highly significant and directly translatable in efforts to develop adoptive Treg therapy. The experiments are well-performed and the data are statistically robust and convincing. Data presentation and writing are clear and concise. Weaknesses are detailed as follows:

**R3, #1:** Although this manuscript is highly significant translationally, a weakness is the lack of mechanistic experiments to better understand how OCRdist controls Treg stability, or how this OCR is targeted by the inflammatory cytokines present during Treg activation/expansion.

**Response to R3, #1:**

We agree that a greater mechanistic understanding of how $OCR_{dist}$ is involved in Treg stability is important. We hypothesize that TCR stimulation in the presence of inflammatory cytokines altered the abundance and/or activities of TFs and other DNA-binding proteins, leading to alteration of chromatin accessibility in destabilizing Tregs. During the revision, we performed additional experiments to gain deeper understanding of specific TFs and their contribution to Treg destabilization. We have added the following new data:

1. *IRF4* $OCR_{dist}$ phylogenetic conservation (new Fig. 6c).
2. Predicted TF binding sites in *IRF4* $OCR_{dist}$ highlighting the presence of AP-1, ETS, and STAT TF binding motifs (Fig. 6b), and predicted AICE, EICE, and ISRE (IRF4-partner composite binding sites) inside *IRF4* $OCR_{dist}$ (Fig. 7a, 7b).
3. Genome-wide TF motif analysis showing increase accessibility in AP-1, ETS, and STAT binding sites induced by IL-1b, IL-6, IL-23 individually and in combination (Fig. 6c).
4. Narrowed the regions of IRF4 $OCR_{dist}$ required for Treg destabilization showing deletion to a 276bp region containing AP-1, ETS, and STAT binding motif (Fig. 6d, 6e).
5. Identified a potential feedforward mechanism for sustaining IRF4 expression in exTregs
6. Over-expression of both IRF4 and BATF (an AP-1 family member) induced Treg destabilization in the absence of inflammatory cytokines (Fig. 7c, 7d, 7e).

Based on these new data, we have extensively revised the discussion to provide an integrated model. Since most of these changes have been included in the response to Reviewer 1 above and easy to locate in the revised manuscript, we are not pasting them here to avoid excessively cluttering this response document.

**Q3, #2:** (Minor) Functional experiments on OCRdist-deleted cells focus only on cell destabilized with the IL-6, IL-1, IL-23 combination. Do other inflammatory cytokines that induce Treg destabilization (albeit less efficiently) as outlined in Fig 1/Fig S1 also promote opening of OCRdist?

We agree that it would be helpful to know how individual inflammatory cytokines influence chromatin accessibility status of IRF4 $OCR_{dist}$. We have performed new experiments to address this point that is also raised by R1. Please refer to response to R1#4 above (Rebuttal page 3).

**Q3, #3:** (Minor) The 'peak linkages' data in Fig 4C is not well-explained. Was this determined from the multi-omics data set? How? Is this based on prior chromatin-confirmation studies? More details are needed to properly contextualize those results.

We apologize for the omission of the method in our original submission. We have added the details to the Methods section (page 13).

> *Peak-expression linkage analyses were performed using the LinkPeaks function in Signac using default parameters based on the scMultiome dataset.*

During the revision, we have now performed additional analysis to include surrounding loci (e.g., *DUSP22, EXOC2*) using our own single-cell multiome dataset and using a published human Tconv promoter-capture Hi-C dataset. Please see response to R1#7 above (Rebuttal page 9).
